# Phenome-wide association of physical activity with morbidity and mortality risk in China: A prospective cohort study

**DOI:** 10.1016/j.xinn.2025.100886

**Published:** 2025-03-20

**Authors:** Yalei Ke, Yuxuan Zhao, Derrick A. Bennett, Neil Wright, Pek Kei Im, Dianjianyi Sun, Pei Pei, Yiping Chen, Ling Yang, Daniel Avery, Feng Ning, Junshi Chen, Zhengming Chen, Jun Lv, Liming Li, Huaidong Du, Canqing Yu, China Kadoorie Biobank Collaborative Group

**Affiliations:** 1 Department of Epidemiology and Biostatistics, School of Public Health, Peking University Health Science Center, Beijing 100191, China; 2 Clinical Trial Service Unit & Epidemiological Studies Unit (CTSU), Nuffield Department of Population Health, University of Oxford, Oxford OX3 7LF, UK; 3 Peking University Center for Public Health and Epidemic Preparedness & Response, Beijing 100191, China; 4 Key Laboratory of Epidemiology of Major Diseases (Peking University), Ministry of Education, Beijing 100191, China; 5 Qingdao Municipal Center for Disease Control and Prevention, Qingdao, Shandong 266033, China; 6 China National Center for Food Safety Risk Assessment, Beijing 100022, China; 7 State Key Laboratory of Vascular Homeostasis and Remodeling, Peking University, Beijing 100191, China

**Keywords:** physical activity, phenome-wide, morbidity, mortality, China

## Abstract

Research in high-income countries has established the health benefits of physical activity (PA), but evidence from low- and middle-income countries, including China, where PA patterns vary from those in high-income countries, remains limited. Moreover, previous research, mainly focused on specific diseases, failing to fully capture the health impacts of PA. We investigated the associations of PA with 425 distinct diseases and 53 causes of death using data from 511,088 participants aged 30–79 years in the China Kadoorie Biobank. Baseline PA was assessed using a questionnaire between 2004 and 2008, and usual PA levels were estimated using the resurvey data in 2013–2014. Cox regression was employed to estimate the associations between PA and outcomes, adjusting for potential confounders. During a median follow-up time of 12 years, 722,183 incident events and 39,320 deaths were recorded across 18 chapters of the International Classification of Diseases, 10th Revision (ICD-10). Total PA was significantly and inversely associated with incidence risks of 14 ICD-10 chapters, specifically 65 diseases and 19 causes of death, with the highest quintile group of PA showing a 14% lower disease incidence and 40% lower all-cause mortality compared with the lowest group. Of these diseases, 54 were not highlighted in World Health Organization PA guidelines. Dose-response analyses revealed L-shaped associations for most PA types, except moderate-to-vigorous intensity PA, which showed a U-shaped relationship. In this population, physical inactivity accounted for 12.8% of PA-related deaths. The findings underscore the broad health benefits of PA across a variety of body systems and the significant disease burden due to inactivity in China, highlighting the urgent need for PA promotion.

## Public summary

• Higher levels of physical activity (PA) link to lower risks of 65 distinct diseases and 19 causes of death.
• Various types and intensities of PA are associated with a lower risk of a wide range of diseases.
• Increasing PA prevents 12% of PA-related deaths.
• There is a critical need to promote PA in populations facing rising rates of non-communicable diseases.

## Graphical abstract

**Figure:**
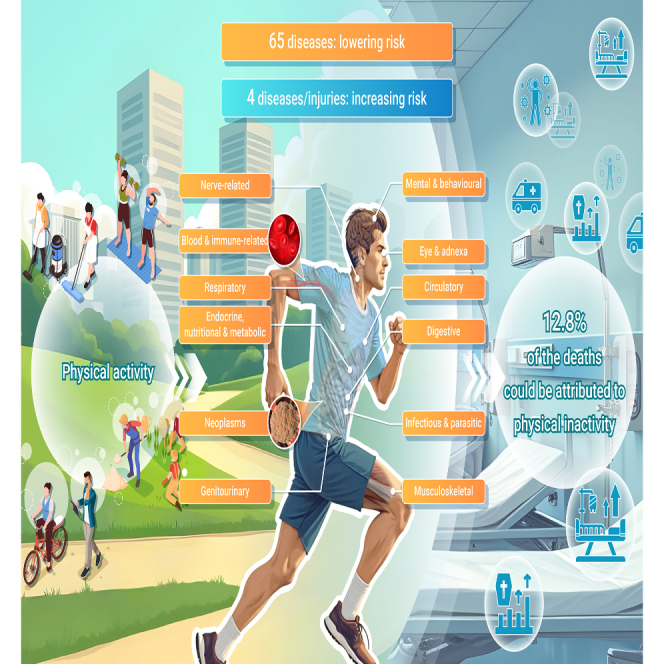

## Introduction

Physical inactivity is associated with the onset of a wide range of chronic diseases and premature deaths, accounting for approximately 3.2 million deaths annually^1^ and imposing a significant disease burden globally, particularly in low- and middle-income countries (LMICs).^2^ Despite this, substantial gaps remain in understanding the full spectrum of health outcomes associated with physical activity (PA), particularly across diverse settings and populations.

Epidemiological studies from Western populations have provided abundant and consistent evidence on the association of PA with health outcomes, mostly focusing on mortality, cardiovascular disease (CVD), and cancer.^3^^,^^4^^,^^5^ Based on these studies, about 22 specific diseases have been identified in the World Health Organization (WHO) PA guidelines as critical or important for decision-making.^6^ These studies have provided valuable insights but are often constrained by methodological differences, selective endpoints, and limited external validity to populations with different PA patterns.^3^ Additionally, such studies are inherently subject to publication bias, as they often target specific diseases.

In LMICs like China, where levels and domains of PA differ from Western contexts, the relationship between PA and health outcomes warrants further exploration. Specifically, leisure-time PA, which is the primary focus of many studies in Western populations, constitutes only ∼10% of total PA in China.^7^^,^^8^ Although a few studies have examined PA in Asian populations, many suffer from limitations such as insufficient sample sizes, restricted geographical coverage, and narrow focus on specific health outcomes.^8^^,^^9^^,^^10^

To fill this gap, we utilized data from the China Kadoorie Biobank (CKB), a large-scale prospective cohort study of ∼0.5 million adults with a 12-year follow-up, to conduct a phenome-wide association study, examining the associations of PA with 425 distinct diseases and 53 specific causes of death. Unlike prior studies that often focus on pre-selected hypotheses, this holistic approach provides a more nuanced understanding of the broader public health implications of PA.

## Materials and methods

### Study population

The CKB study recruited 512,724 participants aged 30–79 years in 10 diverse (5 urban and 5 rural) areas across China at the baseline from 2004 to 2008. At local study assessment clinics, trained health workers administered a laptop-based questionnaire covering sociodemographic characteristics, lifestyle factors, and medical history, and undertook physical measurements. Details of the CKB study design and methods have been reported previously.^11^^,^^12^ Subsequently, two resurveys were conducted in 2008 and 2013–2014, using similar procedures among ∼5% of randomly selected surviving participants. Ethical approvals were obtained by the Ethical Review Committee of the China National Centre for Disease Control and Prevention (Beijing, China) and the Oxford Tropical Research Ethics Committee, University of Oxford (UK). All participants provided written informed consent before participating in the study.

In this study, we excluded participants who were lost to follow-up shortly after baseline (*n* = 1), died or were lost to follow-up before reaching age 35 years (*n* = 9), had both self-reported PA and sedentary leisure time equal to zero (*n* = 81), reported spending more than 20 h daily on all waking activities (*n* = 814), gave implausible or conflicting answers to occupational and commuting-related questions (e.g., reported not working but had nonzero commuting-related PA, *n* = 729), or with missing data on other covariates (i.e., body mass index, BMI, *n* = 2), leaving 511,088 participants for the primary analysis (Figure S1).

### Assessment of PA

Details of the assessment of PA have been previously reported.^13^^,^^14^ Briefly, the CKB PA questionnaire was adapted from validated questionnaires from previous studies,^15^^,^^16^ including questions on the intensity, frequency, and duration of four domains of PA (occupation, commuting, housework, and leisure-time exercise) during the past year (material S2). Based on the updated 2024 Compendium of Physical Activity^17^ (see Table S1 for detailed assignments), metabolic equivalent of task (MET) was used to assess the intensity level of different types of activities, and the MET of each activity was multiplied by the frequency and duration of PA to calculate PA in MET-hours per day (MET-h/day). The total PA level was calculated as the summation of all the MET-h spent on all types of activities. Domain-specific PA levels were calculated by summing all the MET-h/day spent in occupational PA (OPA) (i.e., all PA performed during paid employment; therefore, the analysis of OPA was restricted to participants with paid employment) and non-occupational PA (NOPA) (i.e., commuting, housework, and leisure-time PA). In addition, total physical activities were also classified into two different subtypes according to intensity levels, i.e., low-intensity PA (LIPA) (<3.0 METs), and moderate-to-vigorous intensity PA (MVPA) (≥3.0 METs).

### Assessment of covariates

Covariate information on sociodemographic characteristics (age, sex, education, annual household income, and occupation), lifestyle factors (smoking status, alcohol consumption, fresh fruit consumption, and sedentary leisure time), self-reported health status, personal medical history (diagnosed cancer, ischemic heart disease [IHD], stroke or transient ischemic attack, hypertension, diabetes, emphysema or bronchitis, asthma, tuberculosis, peptic ulcer, gallstones or cholecystitis, chronic hepatitis or cirrhosis, chronic kidney diseases, rheumatoid arthritis, psychasthenia, and psychosomatic disorder), and family history (heart attack, stroke, cancer, and diabetes) was collected at baseline using a laptop-based electronic questionnaire. Baseline physical measurements included body weight, height, and blood pressure. All participants provided a 10 mL random blood sample for an immediate on-site test of plasma glucose. BMI was calculated as measured weight in kilograms divided by height in square meters. Prevalent hypertension was defined as systolic blood pressure ≥140 mmHg, diastolic blood pressure ≥90 mmHg, self-reported doctor-diagnosed hypertension, or self-reported use of antihypertensive drugs at baseline. Diabetes was defined as fasting blood glucose ≥7.0 mmol/L, random blood glucose ≥11.1 mmol/L, or self-reported doctor-diagnosed diabetes.

### Follow-up for morbidity and mortality

The CKB study closely monitors the outcome events of all the participants, including morbidity, mortality, as well as migration and loss to follow-up, using electronic records. Mortality data were collected periodically by local death registries, residential records, and the national health insurance system. Information on morbidity was collected through linkage, using the participant’s unique personal identification number, with disease registries (for cancer, stroke, coronary heart disease, and diabetes) and the national health insurance system (for any hospitalization episode). For participants not linked to those systems, active follow-ups were conducted via direct contact or reports from family members. The same linkage processes and standardized follow-up protocol were applied across all regions, ensuring completeness and consistency in data collection. All events were coded following the International Classification of Diseases, 10th Revision (ICD-10). By the censoring date of December 31, 2018, 56,550 (11.0%) participants had died, 320,490 (62.5%) were ever hospitalized, but only 4,013 (<1.0%) were lost to follow-up.

### Outcome measures

To enable a “phenome-wide” investigation, we reviewed disease events coded by the first three characters of ICD-10 codes, consolidating when appropriate to compile a concise list of distinct diseases based on knowledge about the disease characteristics (Table S2). Several ICD-10 chapters considered irrelevant to the study population (e.g., perinatal-origin diseases [chapter XVI] and congenital conditions [XVII]) were excluded. Statistical analyses were conducted for specific outcomes with ≥100 incident events or deaths to capture a wide range of specific conditions while maintaining statistical precision. Only the first event was considered for individuals experiencing multiple hospitalizations for the same disease. Within each ICD-10 chapter, outcomes with <100 events of a specific disease were combined as "other diseases" of the individual chapter for exploratory analysis.

### Statistical analysis

Participants were categorized into five groups based on the quintile boundaries of each type of PA (total, domain-specific, and intensity-specific) in all participants. There is evidence that the average prevalence rate of physical inactivity in China is 31.0% (30.2%–31.8%).^18^ Therefore, those with total PA levels below the 31st percentile of this population were defined as physically inactive. Means and percentages of baseline characteristics were calculated across total PA quintile groups for a descriptive purpose.

Hazard ratios (HRs) and 95% confidence interval (95% CI) for various diseases associated with total, domain-specific, and intensity-specific PA levels were estimated using Cox proportional hazards regression models. The assumption of proportional hazards was verified using the Schoenfeld residuals. Multivariable analyses were stratified by age-at-risk (5-year groups from 35 to 85 years), sex, and 10 study areas, where appropriate, and adjusted for education (three groups: primary school or below, middle or high school, and technical school/college or above), drinking status (7 groups: never or not weekly, ex-regular, weekly but not daily, current < 15 g/day, current 15–29 g/day, current 30–59 g/day, current ≥ 60 g/day) and smoking status (5 groups: never or occasional, ex-regular, current <15, current 15–24, current ≥25 cigarettes equivalent per day). Additional mutual adjustments for the other domain or type were made in the analyses involving domain- and intensity-specific PA. To control reverse causality, people with prior disease records at baseline were excluded from relevant analyses (e.g., excluding those with prior IHD or stroke in analyses of circulatory diseases). As PA was an exposure variable with five categories, all HRs were calculated using the floating-absolute-risk method to facilitate comparisons between groups.^19^ Subgroup analyses were conducted by baseline age (<65 and ≥65 years), sex, and area (urban and rural), and likelihood ratio tests examined potential effect modification. Participants were censored upon death, lost to follow-up, or December 31, 2018, whichever came first.

Several sensitivity analyses were conducted to test the robustness of the results: (1) additional adjustments for further covariates, including household income (<2,500, 2,500–4,999, 5,000–9,999, 10,000–19,999, 20,000–34,999, ≥35,000 CNY/year), occupation (farmers or workers, other employee, household, or unemployed), consumption frequency of fresh fruits (0, 0.5, 2.0, 5.0, 7.0 days/week), sedentary leisure time, BMI (<18.5, 18.5–23.9, 24–27.9, ≥28.0 kg/m^2^), and family history of CVD, cancer, and diabetes (presence or absence); (2) further exclusion of the first 3 years of follow-up; (3) excluding participants with poor self-reported health or previous major chronic diseases (including self-reported IHD, stroke, transient ischemic attack, cancer, and diabetes) at baseline; (4) fitting a Fine-Gray proportional subdistribution hazards regression model^20^ to account for the competing risks of death; and (5) excluding those who had a disease in the top 50 disability weights as defined by the Global Burden of Disease Study in 2013^21^ (as listed in Table S3) from baseline to the onset of the specific disease.

Diseases showing a significant association with PA in the current analysis were separated into two categories: those "diseases listed in WHO PA guidelines" included diseases associated with PA suggested by the WHO,^6^ which were further categorized into critical (i.e., an outcome that is critical to decision-making) and important (i.e., an outcome that is important but not critical to decision-making) outcomes, including several cancers, type 2 diabetes, hypertension, CVD, anxiety, depression, dementia, sleep disorders, and bone health; while others were categorized as "CKB PA-associated diseases." Detailed information about outcome classifications is included in Table S4.

Restricted cubic splines with three knots were used to graphically estimate the non-linear associations of PA with aggregated WHO and CKB PA-related diseases. To gauge the impacts of measurement errors, short-term within-person variations, and long-term changes in PA levels, we calculated regression dilution ratios (RDRs) using MacMahon’s method^22^ in the sub-cohort of 24,957 participants attending the second resurvey of CKB, which provided a more extended time frame, reflecting participants' usual PA levels over a more extended period compared with the first resurvey. The regression coefficient in restricted cubic splines (natural logarithm of HRs) was then multiplied by 1/RDR to derive HRs (and associated 95% CI) for per-standard deviation (SD) MET-h/day usual PA in relation with previous-mentioned aggregated diseases. Details of the calculation process are shown in Table S5.

To assess the burden of physical inactivity, we estimated the total number of hospitalizations and median days spent in hospital for CKB PA-associated diseases after adjusting for age, sex, and 10 study areas using negative binomial regression and Gamma regression due to their distribution, respectively, for both physically inactive and active participants. Additionally, we analyzed the overall and sex-specific survival of physically inactive groups vs. physically active ones using Kaplan-Meier curves. Incidence and mortality rates (per 100,000 person-years) were calculated as weighted means, stratified by age (in 5-year groups), sex, and 10 study areas. The total incidence and mortality rates were the summation of disease-specific rates. Population attributable risk percent (PAR%) was calculated to estimate the proportion of incident cases or deaths during follow-up that could have been prevented if all participants were physically active, assuming a causal relation. PAR was estimated by *p* × (HR – 1)/HR, where *p* is the prevalence of physical inactivity as defined above,^18^ and HR is the risk of cause-specific morbidity or mortality associated with physical inactivity.

Competing-risk analysis was performed using SAS (version 9.4, SAS Institute, Cary, NC), and all other analyses were conducted with R (version 4.3.1, R Foundation for Statistical Computing, Vienna, Austria). Statistical tests were two-sided, and statistical significance (at the 5% level) was assessed using Benjamini-Hochberg false discovery rate (FDR)^23^ adjusted *p* values across all phenome-wide outcomes. Unless otherwise specified, we emphasize only statistically significant associations after FDR adjustment.

## Results

### Baseline characteristics and follow-up results

Among the 511,088 participants, the mean (SD) age was 52.0 (10.7) years at baseline, with 59.0% women and 44.2% residing in urban areas. The mean (SD) PA level was 20.5 (13.4) MET-h/day. Compared with lower PA level individuals, those with higher PA levels were more likely to be rural residents, be agricultural or factory workers, spend less time in sedentary leisure activities, have lower BMI, and self-reported better health or a lower prevalence of previous chronic diseases (Table 1).

**Table 1:** Baseline characteristics by total physical activity quintile groups

Characteristics	Total physical activity quintile groups (MET-h/day)					Overall
	≤8.59	8.60–13.99	14.00–21.24	21.25–31.89	≥31.90	
	Q_1_	Q_2_	Q_3_	Q_4_	Q_5_	
Number of participants	101,896	102,497	102,242	102,200	102,253	511,088
Physical activity, mean MET-h/day (SD)	5.6 (2.4)	11.3 (1.5)	17.3 (2.1)	26.2 (3.1)	41.9 (8.4)	20.5 (13.4)
Sedentary leisure time, mean h/day (SD)	3.4 (1.8)	3.3 (1.5)	3.0 (1.4)	2.8 (1.4)	2.6 (1.3)	3.0 (1.5)
Sociodemographic characteristics						
Mean age, years (SD)	58.5 (10.8)	54.9 (10.6)	50.7 (10.1)	48.6 (9.2)	47.3 (8.3)	52.0 (10.7)
Women, %	54.4	68.7	61.7	59.1	51.1	59.0
Urban, %	54.5	49.4	48.2	37.7	31.2	44.2
Education >6 years, %	47.1	49.3	56.4	49.1	44.4	49.3
Agricultural or factory workers, %	21.0	38.7	58.9	76.3	83.8	55.8
Household income >20,000 yuan per year, %	36.1	41.2	48.1	41.9	46.6	42.8
Lifestyle risk factors						
Current smokers, %	31.2	22.2	27.4	30.1	36.1	29.4
Current alcohol drinkers, %	13.4	11.8	15.6	15.4	18.0	14.8
Fresh fruit intake <4 days/week, %	71.5	69.0	66.5	73.6	78.5	71.8
Body mass index, mean kg/m^2^ (SD)	24.0 (3.6)	24.0 (3.5)	23.7 (3.3)	23.4 (3.2)	23.3 (3.1)	23.7 (3.4)
Medical history, %						
Poor self-reported health	15.4	10.9	9.1	8.9	7.3	10.3
Previous major chronic disease	27.3	20.0	14.3	11.2	9.5	16.4
Ischemic heart disease	6.1	4.8	2.4	1.1	0.7	3.0
Stroke or transient ischemic attack	4.6	2.1	1.0	0.6	0.4	1.7
Diabetes	10.0	7.8	5.2	3.6	2.9	5.9
Cancer	1.1	0.7	0.4	0.2	0.1	0.5

All *p* for trend <0.001.

MET-h/day, metabolic equivalent of task per hour per day; SD, standard deviation.

**Figure 1:**
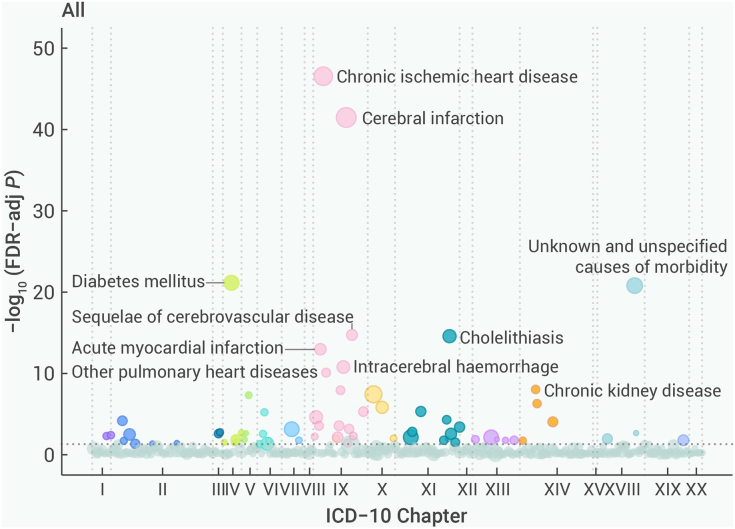
Wide landscapes of diseases associated with the highest quintile group of physical activity after FDR adjustment by ICD-10 chapters The number of diseases with FDR significant associations with physical activity is 69. The y axis represents the negative log_10_ of the phenome-wide *p* value after FDR adjustment. The horizontal gray dashed line indicates the cutoff for 0.05. The size of the point is proportional to the number of cases. The models were stratified by age at risk (5-year groups), sex, and 10 study areas, and were adjusted for education, drinking status, and smoking status. ICD-10, International Classification of Diseases, 10th Revision; FDR, false discovery rate.

During a median [IQR] follow-up of 12.1 [11.1–13.1] years (5,989,090 person-years), 333,940 (65.3%) experienced at least one reported hospitalization or death. A total of 722,183 incident events and 39,320 mortality events occurred across 18 ICD-10 chapters, involving 425 distinct diseases and 53 causes of death, each with at least 100 cases (Figure 1; Tables S4 and S10).

### Chapter-specific morbidity

Total PA was significantly associated with lower risks of 14 chapters, with HRs (95% CI) ranging from 0.61 (0.57–0.66) for mental and behavioral diseases to 0.95 (0.92–0.97) for musculoskeletal diseases in the highest quintile (Q_5_) of total PA, compared with the lowest quintile (Q_1_). Overall, individuals in Q_5_ of total PA had a 14% (0.86, 0.85–0.87) lower risk of developing any disease than those in Q_1_ (Figure S2).

### Disease-specific morbidity

Across the 425 distinct diseases examined, Q_5_ of total PA was significantly associated with 124 outcomes (Tables S10 and S11). Sixty-nine significant associations after the FDR adjustment are presented in Figure 2. Notably, 65 outcomes had a lower risk, with HRs ranging from 0.24 (0.13–0.46) for elevated blood glucose level (R73) to 0.91 (0.87–0.95) for gastritis and duodenitis (K29). In contrast, four diseases or injuries had significant positive associations with total PA (i.e., varicose veins [I83, I85, I86], inguinal hernia [K40], other bursopathies [M71], and injury of unspecified body region [T14]). The HRs for all disease-specific morbidities under each ICD-10 chapter examined are shown in Figures S3–S20.

**Figure 2:**
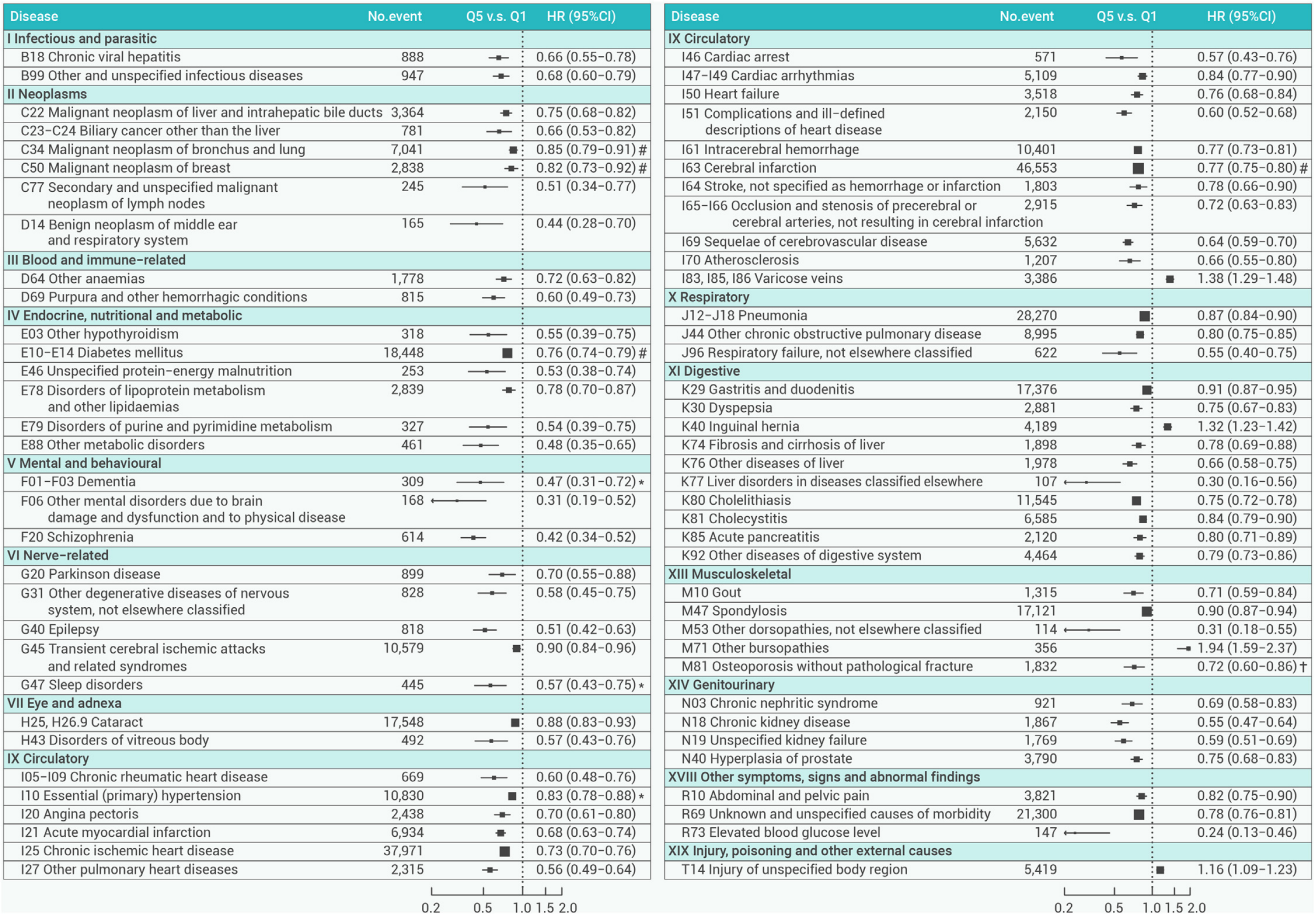
Adjusted HRs for specific diseases showing significant associations after FDR adjustment with physical activity by ICD-10 chapters The x axis is on a log scale. The black squares correspond to the HR values, and the size of the squares is inversely proportional to the standard error; the black horizontal lines represent the 95% confidence interval. HRs were stratified by age at risk (5-year groups), sex, and 10 study areas, and were adjusted for education, drinking status, and smoking status. The individual diseases listed exhibited statistically significant associations between the highest quintile group and the lowest after FDR adjustment in overall analyses. #, an outcome that is critical to decision-making defined by WHO for those aged ≥18 years; ∗, an outcome that is important, but not critical to decision-making defined by WHO for those aged ≥18 years; †, an outcome that is critical to decision-making defined by WHO for those aged ≥65 years. HR, hazard ratio; CI, confidence interval; WHO, World Health Organization; ICD-10, International Classification of Diseases, 10th Revision; FDR, false discovery rate.

The results of the sensitivity analysis, including further adjustment, excluding the first 3 years of follow-up, excluding individuals with poor self-reported health or pre-existing health conditions at baseline, fitting a competing-risk model, and excluding participants who had a disease with a high disability weight prior to the onset of the specific disease, remained robust. Although the associations for a few diseases were not statistically significant, the direction of the associations remained consistent, as shown in Tables S23–S27.

### Morbidity associated with domain- and intensity-specific PA

For domain-specific PA, both OPA and NOPA were inversely related to most diseases or showed directional consistency with total PA. OPA was associated with a lower risk of 19 outcomes, and NOPA was associated with a lower risk of 20 outcomes after FDR adjustment. OPA and NOPA were associated with lower risk for 8 outcomes (Figures S21 and S22; Tables S13 and S14). For intensity-specific PA, although MVPA offered more substantial risk reduction for a broader range of diseases, LIPA mitigated some of the risks associated with MVPA, such as inguinal hernias (K40) and injury of unspecified body region (T14) (Figures S23 and S24; Tables S15 and S16).

### Disease-specific morbidity in subgroups

**Figure 3:**
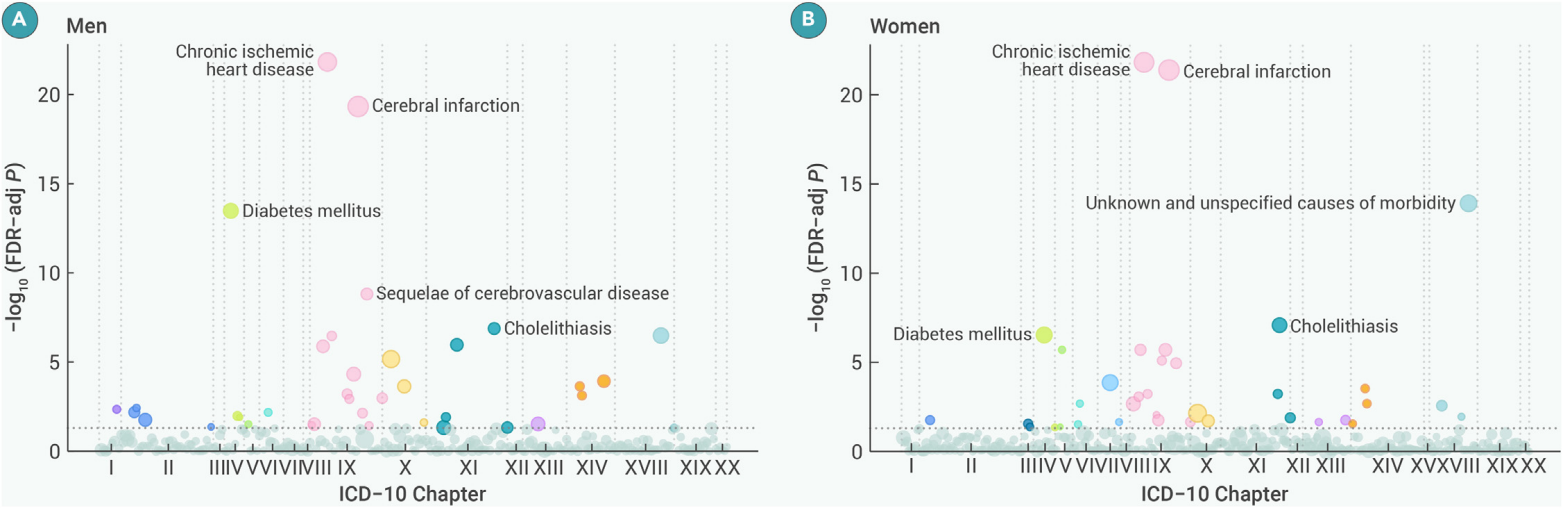
Wide landscapes of diseases associated with the highest quintile group of physical activity after FDR adjustment by ICD-10 chapters in sex subgroups The number of diseases with a significant FDR association with physical activity is 37 and 36 for men and women, respectively. The y axis represents the negative log_10_ of the phenome-wide *p* value after FDR adjustment. The horizontal gray dashed line indicates the cutoff for 0.05. The size of the point is proportional to the number of cases. The models were stratified by age at risk (5-year groups), sex, and 10 study areas, where appropriate, and were adjusted for education, drinking status, and smoking status. ICD-10, International Classification of Diseases, 10th Revision; FDR, false discovery rate.

**Figure 4:**
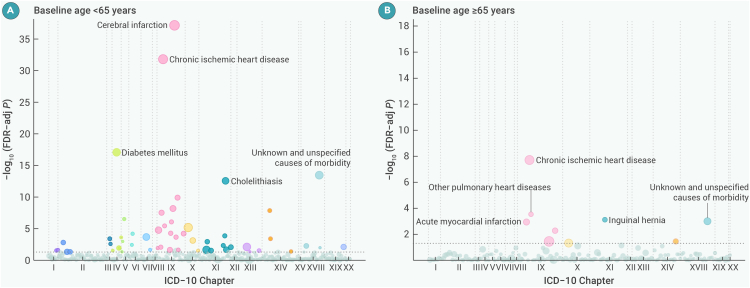
Wide landscapes of diseases associated with the highest quintile group of physical activity after FDR adjustment by ICD-10 chapters in age subgroups The number of diseases with a significant FDR association with physical activity is 55 and 9 for the groups aged <65 and ≥65 years, respectively. The y axis represents the negative log_10_ of the phenome-wide *p* value after FDR adjustment. The horizontal gray dashed line indicates the cutoff for 0.05. The size of the point is proportional to the number of cases. The models were stratified by age at risk (5-year groups), sex and, 10 study areas, where appropriate, and were adjusted for education, drinking status, and smoking status. ICD-10, International Classification of Diseases, 10th Revision; FDR, false discovery rate.

In subgroup analyses, diseases significantly associated with PA were similar in both sexes, but PA was associated with a lower risk of some neoplasms only in men (Figures 3 and S25; Tables S17 and S18). The number of diseases inversely associated with total PA was higher in those aged <65 years than in those aged ≥65 years (Figures 4 and S26; Tables S19 and S20). There was an interaction between urban and rural areas on the association between total PA and the risk of most diseases of the circulatory, respiratory, digestive, musculoskeletal, and genitourinary systems (Figures S27 and S28; Tables S21 and S22). Further analyses by different domains showed that NOPA was significantly inversely associated with 22 diseases in urban areas, compared with only 4 in rural areas (Table S7); conversely, OPA was significantly associated with a lower risk of 19 diseases in rural areas (Table S8).

### Diseases listed in WHO PA guidelines vs. CKB PA-associated diseases

**Figure 5:**
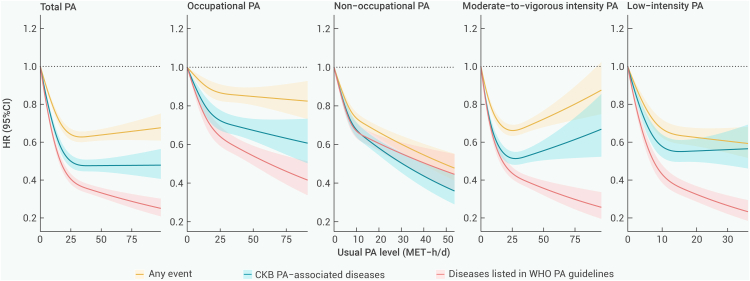
Associations of selected PA-related diseases with total and domain-specific PA levels Restricted cubic splines with three knots were used to graphically estimate the associations of PA with aggregated diseases. Solid lines represent HRs, and the shaded areas represent 95% CIs. All *p* values for nonlinearity ≤0.001. HRs were stratified by age at risk (5-year groups), sex, and 10 study areas, and were adjusted for education, drinking status, and smoking status. In the occupational and non-occupational, moderate-to-vigorous intensity and low-intensity PA analyses, additional mutual adjustments were made. PA, physical activity; WHO, World Health Organization; CKB, China Kadoorie Biobank; HR, hazard ratio; CI, confidence interval; MET-h/day, metabolic equivalent of task per hour per day.

Out of the 65 outcomes negatively associated with total PA, 11 were deemed critical or important outcomes associated with PA for the adult population, as outlined in the WHO guidelines,^6^ which were defined as "Diseases listed in WHO PA guidelines" in this study (Figures 2 and S2). For all aggregated diseases, there was an inverse trend of decreasing at a slower rate with higher levels of PA. The trend of the risk reduction from NOPA was more significant than that from OPA, and the RCS curve between MVPA and any event risk was U-shaped (Figure 5).

### PA-associated mortality

Overall, the Q_5_ of total PA was associated with a 40% (0.60, 0.58–0.62) lower risk of all-cause mortality (Figure S29). Total PA was significantly associated with lower risks of 19 causes of death from 8 chapters after FDR adjustment (Table S6), with HRs ranging from 0.26 (0.14–0.49) for chronic rheumatic heart disease (I05–I09) to 0.80 (0.73–0.88) for malignant neoplasm of bronchus and lung (C34) (Figure S29; Table S12). Except for malignant neoplasm of the colon (C18), unspecified chronic bronchitis (J42), other sudden death, cause unknown (R96), and other ill-defined and unspecified causes of mortality (R99), the other 15 mortality outcomes also exhibited significant associations, albeit generally modest, after FDR adjustment in the morbidity analyses (Table S4).

### Attributable risks and disease burden of physical inactivity

**Figure 6:**
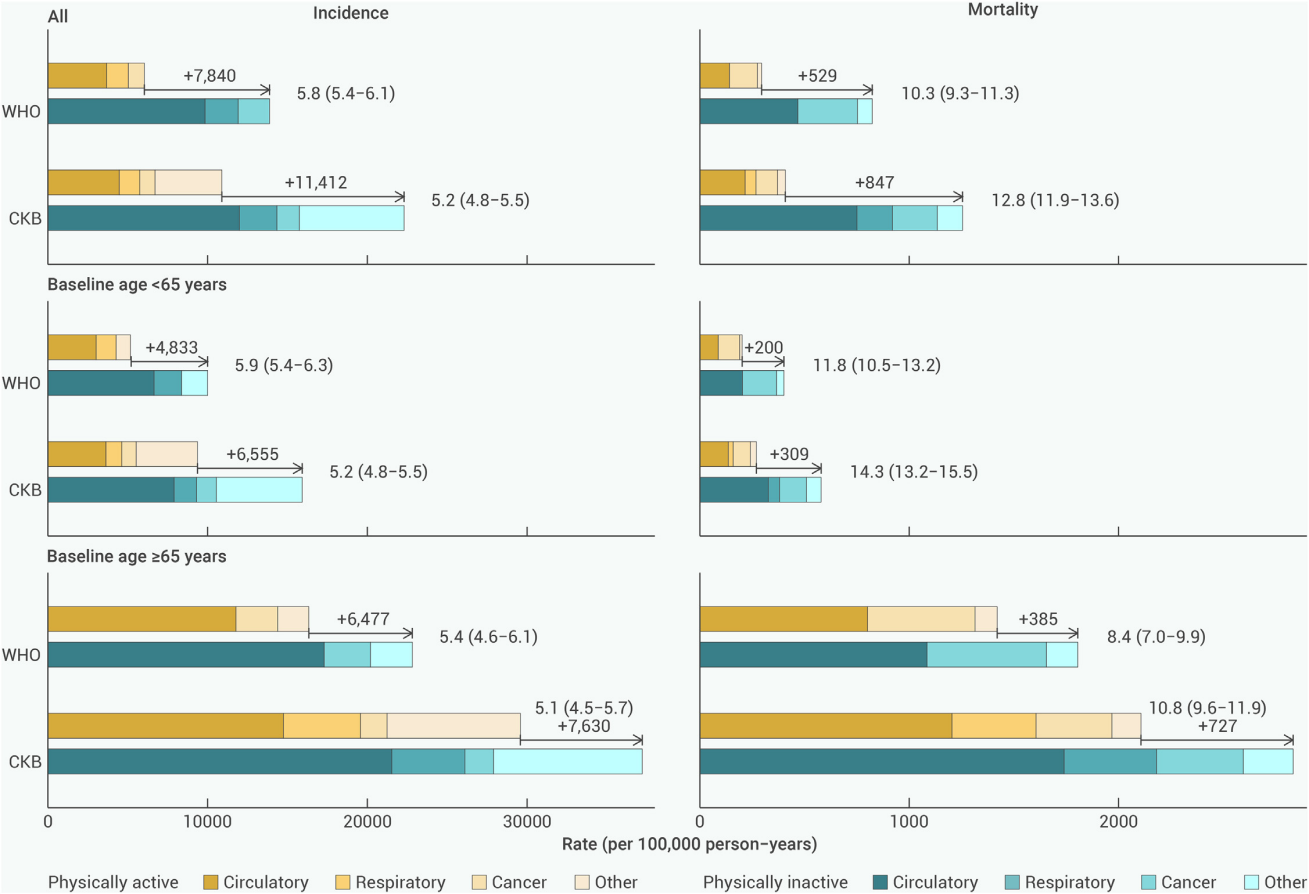
Incidence and mortality rates from CKB PA-related diseases and diseases listed in WHO PA guidelines The bar diagrams showed absolute incidence and mortality rates per 100,000 person-years for physically active and inactive participants. The numbers on the bar graph are the absolute excess incidence or mortality rates in the physically inactive group compared with the physically active group, as well as PAR% (95% confidence interval) for CKB PA-related diseases and diseases listed in WHO PA guidelines, both overall and separately for those <65 and ≥65 years. PAR% was calculated from hazard ratio stratified by age at risk (5-year groups), sex, and 10 study areas and adjusted for education, drinking status, and smoking status. PA, physical activity; PAR%, population attributable risk percent; WHO, World Health Organization; CKB, China Kadoorie Biobank.

Compared with those who were physically active (with total PA >11.5 MET-h/day), the absolute excess incidence rate of CKB PA-associated diseases per 100,000 person-years was 11,412 (6,555 for participants <65 years and 7,630 for those ≥65 years) in the physically inactive group and the corresponding number of diseases listed in WHO PA guidelines was 7,840 (4,833 for those <65 years; 6,477 for those ≥65 years). In terms of mortality, the excess of CKB PA-associated deaths and deaths from diseases listed in WHO PA guidelines due to physical inactivity was 847 (309 for those <65 years; 727 for those ≥65 years) and 529 (200 for those <65 years; 385 for those ≥65 years) per 100,000 person-years. Assuming all FDR-adjusted significant associations were causal, physical inactivity could account for 12.8% of CKB PA-associated causes of death (Figure 6) and 10.5% of all-cause mortality in this population (Table S9). Further analysis revealed that, if causal, physical inactivity might contribute to 26.9% of chronic rheumatic heart disease (I05–I09) deaths, 15.5% of pneumonia (J12–J18) deaths, and 14.6% of diabetes mellitus (E10–E14) deaths, as listed in Table S9.

As shown in Figure S30, individuals who were physically active, regardless of sex, had better survival compared with the physically inactive. The physically active group will gain an additional 3.8, 3.8, 2.8, and 1.9 years of lifespan, respectively, at the same survival probability compared with the physically inactive aged 50, 60, 70, and 80 years. At the age of 35–75 years, the physically inactive group had a greater frequency of hospitalizations and extended hospital stays for CKB PA-associated diseases than the physically active group (Figure S31).

## Discussion

This prospective study provides a comprehensive evaluation of the long-term health effects of PA on a broad spectrum of disease outcomes in Chinese adults. Overall, a higher level of PA was associated with lower risk of developing 65 diseases, 54 of which are not included in the WHO guidelines. Compared with other major risk factors such as smoking^24^ and alcohol,^25^ PA appears to be associated with a lower risk of a wide range of diseases, suggesting that PA may be an important modifiable factor in reducing the risk of many diseases, particularly in women. Furthermore, physical inactivity is associated with a higher risk of hospitalization and death. Both OPA and NOPA, and LIPA and MVPA, were associated to some extent with a reduced risk of developing these diseases.

Previous observational studies from developed countries have explored the associations of various disease morbidity and mortality outcomes with PA and have found many significant associations.^3^^,^^4^ However, most previous studies have not been able to investigate associations with multiple diseases simultaneously. A study based on objectively measured PA in the UK biobank (UKB) explored the association of MVPA with the incidence of 697 diseases and found that MVPA was associated with reduced risks of 373 (54%) diseases at 6.3 years of follow-up.^26^ These findings are consistent with most of our results but were limited to MVPA only. With a minimum number of events of 10 for the included diseases, it is possible that some associations were missed due to insufficient power.

Some of the diseases listed in the WHO PA guidelines but not confirmed in this study are mainly cancers, including esophageal, gastric, endometrial, kidney, and bladder. For example, inverse associations between PA and esophageal, gastric, and kidney cancer were also observed in this study but failed to pass the FDR correction. Also, the evidence for the associations between PA and these cancers in China or Asia is minimal and inconsistent,^10^^,^^27^^,^^28^^,^^29^ so more evidence is needed to clarify these associations. Existing evidence on anxiety and depression predominantly derives from psychometric assessments using validated clinical scales.^30^^,^^31^ Some have found that PA is slightly more strongly associated with depressive symptoms (OR = 0.84; 95% CI, 0.80–0.89) than with major depression (OR = 0.86; 95% CI, 0.76–0.98).^31^ Our study, however, is based on health insurance records that mainly capture clinically diagnosed disorders rather than subclinical symptom profiles. This methodological divergence is compounded by systematic challenges in China’s mental healthcare landscape, including the substantial underdiagnosis rate and undertreatment rate (∼10%) of psychological disorders.^32^ These limitations, coupled with the absence of a multidimensional mental health evaluation in the CKB cohort (e.g., neuropsychological testing or symptom severity grading), likely attenuated the observable associations, which underscore the necessity of implementing hybrid assessment protocols in future research, integrating both diagnostic registries and standardized psychometric tools to better delineate PA’s neuroprotective effects across the mental health continuum.

In addition, this study identified 54 PA-associated diseases across various body systems that were not considered critical or important to decision-making in the WHO PA guidelines. New insights were provided in respiratory, psychiatric, digestive, liver, kidney, and ophthalmic diseases. There are gaps or contradictory findings in previous studies on these diseases. For example, results from UKB suggested that the risk of COPD decreased as total PA increased.^33^ Still, a case-control study from the National Health and Nutrition Examination Survey^34^ found no association. More research is needed to confirm these findings.

For varicose veins, we have found a 38% higher risk of PA in the Q_5_ group, and the result remained robust after further adjustment for other covariates or exclusion of those with poor self-reported health and prior health conditions. A previous cohort study from Denmark has reported that prolonged standing or walking and heavy lifting are associated with an increased risk of varicose veins.^35^ However, in the Framingham Study, varicose veins were associated with lower PA levels in both men and women,^36^ possibly due to reverse causality—where having varicose veins reduces the ability to engage in PA. Also, we found a higher risk of inguinal hernia and other bursopathies in the Q_5_ group. More vigorous PA is a risk factor for inguinal hernia,^37^ and a study using accelerometer-measured PA from UKB^5^ has yielded similar results. We suggest that these findings are related to the higher proportion of heavy PA in the Chinese population. This is supported by the higher elevated risk of these diseases observed in OPA, where prolonged or weight-bearing standing and sudden increases in intra-abdominal pressure may increase the risk of these conditions.^37^

Many studies have explored the health effects of NOPA,^3^ while the health role of OPA remains uncertain and is sometimes described as a paradox.^38^ OPA constitutes a significant part of total PA in China,^39^ as well as other LMICs.^7^ Our study found that OPA also reduced the risk of a considerable number of diseases (e.g., cerebral infarction, chronic ischemic heart disease, diabetes mellitus, cholelithiasis, and intracerebral hemorrhage), several of which were confirmed in previous reviews,^40^ but most of which were found for the first time.^38^ Without prior evidence, our findings could only be considered hypothesis-generating, requiring further verification in other studies. Further analysis revealed that OPA was particularly associated with a lower risk of numerous diseases in rural areas, with 19 associations showing FDR-adjusted significance. In contrast, although no “OPA paradox” was observed in urban areas, the number of diseases associated with OPA was significantly lower, and none of these associations passed the FDR correction. This discrepancy may partially explain the observed protective effect of OPA in this study, where agricultural labor plays a key role in rural areas. It is worth noting that, while the findings from the Copenhagen General Population Study^38^ have adjusted for many socioeconomic factors, they have not fully considered the impact of agricultural work, which may contribute to the observed differences in the effects of OPA across different regions.

Most evidence supports MVPA in improving CVD risk factors, leading health promotion programs, and public health guidelines to emphasize MVPA with relatively little consideration given to activities of lower intensity.^41^ Recently, a growing number of studies have suggested that LIPA may also provide benefits in preventing some diseases,^42^ in addition to the benefits found in this study for respiratory disease and psychosomatic disorders. LIPA is easier to perform and adhere to, especially in populations with limitations in MVPA performance. To provide more options for increasing PA levels, the evidence on the role of LIPA should be refined. On the other hand, we also found a U-shaped association between MVPA and the risk of developing any event, where excessively high levels of MVPA (e.g., exceeding 25 MET-h/day) attenuated the health benefits of PA. This suggests that MVPA should be cautiously undertaken to avoid potential risks associated with high PA levels.

WHO PA guidelines provided differentiated exercise recommendations for adults aged 18–64 years and older populations aged ≥65 years, and indicated that, in addition to preventing chronic diseases such as CVD and diabetes, PA plays a crucial role in improving cognitive function, reducing musculoskeletal issues (e.g., osteoporosis, falls, fractures), and maintaining functional independence in the ≥65 years group.^6^ However, the present study found that the ≥65 years group tended to have lower levels of PA, and the number of associations between PA and disease was also lower than in the <65 years group. On the one hand, older adults often experience a decline in physical capacity. They are usually in a state of co-morbidity that limits mobility, potentially weakening the observed associations between PA and disease outcomes. On the other hand, older adults are often studied in the context of functional health, fall prevention, and cognitive outcomes. Still, these aspects are less frequently addressed in the ICD-10 code-defined disease outcomes, which may explain why fewer disease associations exist.

The strengths of this study include the large sample size, detailed measurements of different domains and intensities of PA, completeness of follow-up, and a wide range of morbidity and mortality outcomes coverage. However, the study is not without its drawbacks. Firstly, PA in this study was assessed through self-reporting, a feasible and widely adopted method in large-scale epidemiological investigations, and may suffer from information bias. To mitigate potential measurement errors, we analyzed PA exposure using population quintiles rather than relying on absolute MET values. It should be noted that accelerometer-derived PA measurements, while providing more objective quantification of movement patterns, capture fundamentally different dimensions of PA behavior compared with self-reported data. The latter modality retains its prominence in current PA guidelines as it incorporates individuals’ subjective interpretation and conscious awareness of their PA. Nevertheless, the discordance in informational content between objective monitoring and self-reported measures of PA (as demonstrated in Figure S32 with r = 0.26) underscores the need for further research to elucidate their conceptual correspondence.^26^ Additionally, the effects of longitudinal PA trajectories on health were not estimated in this study; however, we estimated usual PA using two repeat PA measures among 5% of participants. Secondly, the medical history information available for analysis was limited to 16 chronic diseases. For example, we found a strong association between PA and a lower risk of dorsopathies. Still, as PA is generally recommended for patients with dorsopathies, especially for early and mild stages, there is a possibility of causal inversion of this finding. Therefore, although excluding the first 3 years of follow-up supports most of our findings, reverse causation bias cannot be completely ruled out. Thirdly, we were unable to study diseases that do not typically lead to hospital admissions (e.g., anxiety and depression), nor rare diseases due to the small number of cases. Fourthly, for certain diseases associated with PA (e.g., gastric, prostate, and kidney cancers), the use of FDR adjustment may have masked true but modest associations. Fifthly, the associations between PA and specific diseases might be influenced by the occurrence of other diseases when conducting a phenome-wide association study. Although our sensitivity analyses excluded participants who developed diseases with high disability weights during the follow-up of the analyzed disease and found the results largely robust, the potential impact of comorbidities may still exist. Finally, the participants included in this study were in relatively good health, as we excluded study participants with associated diseases at baseline when analyzing specific diseases, and the CKB sample is not nationally representative, so the generalization of our results to other populations should be cautious.

Research on the disease burden associated with physical inactivity in China is limited. We showed that physical inactivity led to an excess absolute incidence rate of 11,412 per 100,000 person-years and could account for 12.8% of the deaths from PA-related causes and 10.5% of all-cause mortality in our study, which was slightly higher than all-cause mortality attributable to physical inactivity in the same period at CKB baseline globally (9.4%) and in North America (9.9%).^43^ Notably, data from 2010 to 2018 indicated an upward trend in physical inactivity among Chinese adults, with notable declines in occupational and commuting-related PA.^39^ The findings of our study provide reliable evidence that, if the current trends in physical inactivity persist, the future burden of disease attributed to physical inactivity in China is likely to increase markedly. Therefore, it is crucial to implement targeted strategies, such as nationwide public health campaigns, community-based exercise programs, and policy interventions that promote active transportation and workplace wellness initiatives, to prioritize and effectively promote PA as a key public health intervention in China.

In summary, this study demonstrates that PA may lower the risk of morbidity due to numerous diseases across a variety of body systems. These findings underscore the significant role of PA in reducing the burden of chronic diseases and improving overall public health. Public health campaigns and policies promoting PA may yield substantial health benefits and help alleviate the growing burden of non-communicable diseases in China.

## Resource availability

### Materials availability

This study did not generate new unique materials or reagents.

### Data and code availability

Details on accessing China Kadoorie Biobank data and the data release schedule are available from www.ckbiobank.org/data-access. Code is available from the corresponding author upon reasonable request.

## Funding and acknowledgments

The most important acknowledgment is to the participants in the study and the members of the survey teams in each of the 10 regional centers, as well as to the project development and management teams based in Beijing, Oxford, and the 10 regional centers. This work was supported by the Noncommunicable Chronic Diseases-National Science and Technology Major Project (2023ZD0510100). The CKB baseline survey and the first re-survey were supported by the Kadoorie Charitable Foundation in Hong Kong. The long-term follow-up has been supported by 10.13039/100004440Wellcome grants to Oxford University (212946/Z/18/Z, 202922/Z/16/Z, 104085/Z/14/Z, 088158/Z/09/Z) and grants (2016YFC0900500) from the 10.13039/501100012166National Key Research and Development Program of China, the 10.13039/501100001809National Natural Science Foundation of China (82192900, 82388102, 81390540, 91846303, 81941018), and the Chinese Ministry of Science and Technology (2011BAI09B01). The 10.13039/501100000265UK Medical Research Council (MC_UU_00017/1, MC_UU_12026/2, MC_U137686851), 10.13039/501100000289Cancer Research UK (C16077/A29186; C500/A16896), and the 10.13039/501100000274British Heart Foundation (CH/1996001/9454), provide core funding to the Clinical Trial Service Unit and Epidemiological Studies Unit at Oxford University for the project. The funders had no role in study design, data collection and analysis, decision to publish, or preparation of the manuscript.

## Author contributions

Conceptualization, J.C., L.L., and Z.C.; data curation, D.A.; formal analysis, Y.K.; funding acquisition, J.L., Z.C., L.L., and C.Y.; investigation, P.P. and F.N.; methodology, D.A.B., N.W., P.K.I., and H.D.; project administration, J.L., D.S., L.Y., and C.Y.; software, D.A.; supervision, C.Y. and H.D.; validation, Y.Z.; visualization, Y.K.; writing – original draft, Y.K. and C.Y.; writing – review & editing, C.Y., H.D., Y.Z., D.A.B., N.W., P.K.I., D.S., P.P., Y.C., L.Y., D.A., F.N., J.C., Z.C., J.L., and L.L. All authors contributed to the manuscript and approved the final version.

## Declaration of interests

The authors declare no competing interests.

## Supplemental information

Data S1. Tables S10–S27

Document S2. Article plus supplemental information
