# Supplementary material for: Phenome-wide association of physical activity with morbidity and mortality risk in China: A prospective cohort study
Source: Innovation (Camb). 2025 Mar 20;6(7):100886. doi: 10.1016/j.xinn.2025.100886 (PMC12277735; doi:10.1016/j.xinn.2025.100886)
Supplement: Document S1. Figures S1–S32, Tables S1–S9, and material S1 and S2 [file mmc1.pdf]

The Innovation, Volume *6*

Supplemental Information

Phenome-wide association of physical activity with morbidity and mortality risk in China: A prospective cohort study

Yalei Ke, Yuxuan Zhao, Derrick A. Bennett, Neil Wright, Pek Kei Im, Dianjianyi Sun, Pei Pei, Yiping Chen, Ling Yang, Daniel Avery, Feng Ning, Junshi Chen, Zhengming Chen, Jun Lv, Liming Li, Huaidong Du, Canqing Yu, and China Kadoorie Biobank Collaborative Group

Supplemental Information

Contents

Supplemental Material 1: Members of the China Kadoorie Biobank collaborative group 1

Supplemental Material 2: China Kadoorie Biobank study questionnaire on physical activity and sedentary leisure time* 2

Table S1: Physical activity types, MET values, codes and intensity categories* 4

Table S2: ICD-10 codes consolidated in phenome-wide investigation 5

Table S3: ICD-10 codes for diseases excluded in sensitivity analysis 5 (disease in the top 50 disability weights as defined by the 2013 GBD study*) 6

Table S4: Outcome classifications of aggregate endpoints........................ 7

Table S5: Estimated regression dilution ratio for physical activity 10

Table S6: Summary of number of mortality events with at least 100 events associated with physical activity by ICD-10 chapter........................ 11

Table S7: Adjusted HRs for specific diseases showing significant associations with non-occupational physical activity after FDR adjustment in rural and urban regions 12

Table S8: Adjusted HRs for specific diseases showing significant associations with occupational physical activity after FDR adjustment in rural and urban regions........................ 13

Table S9: Multivariable-adjusted HRs (95%CIs) and PAR%(95%CIs) for physical inactivity in relation to specific diseases 14

Figure S1: Flow diagram of study participants........................ 15

Figure S2: Adjusted HRs for ICD-10 chapter-specific incidence associated with physical activity........ 16

Figure S3: Adjusted HRs for incidence of specific types of infectious and parasitic diseases associated with physical activity 17

Figure S4: Adjusted HRs for incidence of specific types of neoplasms associated with physical activity 18

Figure S5: Adjusted HRs for incidence of specific types of blood and immune-related diseases associated with physical activity 19

Figure S6: Adjusted HRs for incidence of specific types of endocrine, nutritional and metabolic diseases associated with physical activity........................ 20

Figure S7: Adjusted HRs for incidence of specific types of mental and behavioural disorders associated with physical activity 21

Figure S8: Adjusted HRs for incidence of specific types of neurological diseases associated with physical

activity.....22
Figure S9: Adjusted HRs for incidence of specific types of eye and adnexa diseases associated with physical activity.....23
Figure S10: Adjusted HRs for incidence of specific types of ear and mastoid process diseases associated with physical activity.....24
Figure S11: Adjusted HRs for incidence of specific types of circulatory diseases associated with physical activity.....25
Figure S12: Adjusted HRs for incidence of specific types of respiratory diseases associated with physical activity.....26
Figure S13: Adjusted HRs for incidence of specific types of digestive diseases associated with physical activity.....27
Figure S14: Adjusted HRs for incidence of specific types of skin and subcutaneous tissue diseases associated with physical activity.....28
Figure S15: Adjusted HRs for incidence of specific types of musculoskeletal diseases associated with physical activity.....29
Figure S16: Adjusted HRs for incidence of specific types of genitourinary diseases associated with physical activity.....30
Figure S17: Adjusted HRs for incidence of specific types of pregnancy-related diseases associated with physical activity.....31
Figure S18: Adjusted HRs for incidence of other symptoms, signs and abnormal findings associated with physical activity.....32
Figure S19: Adjusted HRs for incidence of specific types of injury, poisoning and other external causes associated with physical activity.....33
Figure S20: Adjusted HRs for incidence of specific external causes associated with physical activity.....34
Figure S21: Wide landscapes of diseases associated with the highest quintile group of domain-specific physical activity increment after FDR adjustment by ICD-10 chapters.....35
Figure S22: Adjusted HRs for specific diseases showing significant associations with domain-specific physical activity after FDR adjustment by ICD-10 chapters.....36
Figure S23: Wide landscapes of diseases associated with the highest quintile group of intensity-specific physical activity increment after FDR adjustment by ICD-10 chapters.....37
Figure S24: Adjusted HRs for specific diseases showing significant associations with intensity-specific physical activity after FDR adjustment by ICD-10 chapters.....38

Figure S25: Adjusted HRs for specific diseases showing significant associations with physical activity after FDR adjustment by ICD-10 chapters in men and women 39
Figure S26: Adjusted HRs for specific diseases showing significant associations with physical activity after FDR adjustment in age group 40
Figure S27: Wide landscapes of diseases associated with the highest quintile group of physical activity after FDR adjustment by region 41
Figure S28: Adjusted HRs for specific diseases showing significant associations with physical activity after FDR adjustment by ICD-10 chapters in rural and urban regions 42
Figure S29: Adjusted HRs for all-cause and cause-specific mortality associated with physical activity. 43
Figure S30: Kaplan-Meier curves for overall survival 44
Figure S31: Total number of hospitalisations median days in hospital and from CKB PA-associated diseases 45
Figure S32: Pearson correlation coefficients for accelerometer-estimated and self-reported PAs in the CKB third resurvey (n=20,190) 46

Supplemental Material 1: Members of the China Kadoorie Biobank collaborative group

International Steering Committee: Junshi Chen, Zhengming Chen (PI), Robert Clarke, Rory Collins, Liming Li (PI), Jun Lv, Richard Peto, Robin Walters.

International Co-ordinating Centre, Oxford: Daniel Avery, Maxim Barnard, Derrick Bennett, Ruth Boxall, Ka Hung Chan, Yiping Chen, Zhengming Chen, Charlotte Clarke, Jonathan Clarke; Robert Clarke, Huaidong Du, Ahmed Edris Mohamed, Hannah Fry, Simon Gilbert, Pek Kei Im, Andri Iona, Maria Kakkoura, Christiana Kartsonaki, Hubert Lam, Kuang Lin, James Liu, Mohsen Mazidi, Iona Millwood, Sam Morris, Qunhua Nie, Alfred Pozarickij, Maryam Rahmati, Paul Ryder, Dan Schmidt, Becky Stevens, Iain Turnbull, Robin Walters, Baihan Wang, Lin Wang, Neil Wright, Ling Yang, Xiaoming Yang, Pang Yao.

National Co-ordinating Centre, Beijing: Xiao Han, Can Hou, Qingmei Xia, Chao Liu, Jun Lv, Pei Pei, Dianjianyi Sun, Canqing Yu, Lang Pan.

10 Regional Co-ordinating Centres: Qingdao CDC: Zengchang Pang, Ruqin Gao, Shanpeng Li, Haiping Duan, Shaojie Wang, Yongmei Liu, Ranran Du, Yajing Zang, Liang Cheng, Xiaocao Tian, Hua Zhang, Yaoming Zhai, Feng Ning, Xiaohui Sun, Feifei Li. **Licang CDC:** Silu Lv, Junzheng Wang, Wei Hou. **Heilongjiang Provincial CDC:** Wei Sun, Shichun Yan, Xiaoming Cui. **Nangang CDC:** Chi Wang, Zhenyuan Wu,Yanjie Li, Quan Kang. **Hainan Provincial CDC:** Huiming Luo, Tingting Ou. **Meilan CDC:** Xiangyang Zheng, Zhendong Guo, Shukuan Wu, Yilei Li, Huimei Li. **Jiangsu Provincial CDC:** Ming Wu, Yonglin Zhou, Jinyi Zhou, Ran Tao, Jie Yang, Jian Su. **Suzhou CDC:** Fang Liu, Jun Zhang, Yihe Hu, Yan Lu, Liangcai Ma, Aiyu Tang, Shuo Zhang, Jianrong Jin, Jingchao Liu. **Guangxi Provincial CDC:** Mei Lin, Zhenzhen Lu. **Liuzhou CDC:** Lifang Zhou, Changping Xie, Jian Lan,Tingping Zhu,Yun Liu, Liuping Wei, Liyuan Zhou, Ningyu Chen, Yulu Qin, Sisi Wang. **Sichuan Provincial CDC:** Xianping Wu, Ningmei Zhang, Xiaofang Chen, Xiaoyu Chang. **Pengzhou CDC:** Mingqiang Yuan, Xia Wu, Xiaofang Chen, Wei Jiang, Jiaqiu Liu, Qiang Sun. **Gansu Provincial CDC:** Faqing Chen, Xiaolan Ren, Caixia Dong. **Maiji CDC:** Hui Zhang, Enke Mao, Xiaoping Wang, Tao Wang, Xi zhang. **Henan Provincial CDC:** Kai Kang, Shixian Feng, Huizi Tian, Lei Fan. **Huixian CDC:** XiaoLin Li, Huarong Sun, Pan He, Xukui Zhang. **Zhejiang Provincial CDC:** Min Yu, Ruying Hu, Hao Wang. **Tongxiang CDC**: Xiaoyi Zhang, Yuan Cao, Kaixu Xie, Lingli Chen, Dun Shen. **Hunan Provincial CDC:** Xiaojun Li, Donghui Jin, Li Yin, Huilin Liu, Zhongxi Fu. **Liuyang CDC:** Xin Xu, Hao Zhang, Jianwei Chen,Yuan Peng, Libo Zhang, Chan Qu.

Supplemental Material 2: China Kadoorie Biobank study questionnaire on physical activity and sedentary leisure time*

Section A: For non-farmers

1. In the past 12 months, how active were you at work?

☐ Mainly sedentary (e.g. office worker)
☐ Standing occupation (e.g. guard, shop assistant)
☐ Manual work (e.g. plumber, carpenter)
☐ Heavy manual work (e.g. miner, construction worker)
☐ Retired, housewife/husband, unemployed, or disabled → *go to Question 12*

2. In a typical week, about how many hours did you usually work? ___________ hrs
3. In the past 12 months, how did you usually get to and from work?

☐ Mainly walk ☐ By bicycle
☐ By motorbike ☐ By bus/car/ferry/train
☐ Mainly stayed at home, or work near home → *go to Question 12*

4. How much time did you spend each day on the journey to and from work? _________mins

Section B: For farmers

5. In the past 12 months, did your farming work change seasonally?

☐ No → *go to Question 7*
☐ Yes

6. In the farming season in the last 12 months:
   - How many months did the farming season usually last? ___________ mths
   - What types of farming work did it usually involve?

☐ Manual ☐ Semi-mechanized ☐ Fully mechanized

   - How many hours did you usually work each day? ___________ hrs
   - Of which, how many hours did you sweat or have a much faster heartbeat? ___________hrs

7. In a typical week (in non-farming seasons), how many hours did you usually work in the field? ___________hrs
8. Apart from the agriculture work, did you have any other job?

☐ No → *go to Question 11*
☐ Yes

9. How active were you at work with the other job?

☐ Mainly sedentary ☐ Mainly general manual work
☐ Mainly standing ☐ Mainly heavy manual work

10. In a typical week, about how many hours did you spent at the other job? ________hrs
11. In a typical day how much time did you usually spend on the journey to and from work on foot or by bicycle? ________ mins

Section C: For both farmers and non-farmers

12. In the past 12 months, how often did you exercise in your leisure time?

☐ Never or almost never } → *go to Question 15*
☐ 1-3 times/month }
☐ 1-2 times/week
☐ 3-5 times/week
☐ Daily or almost every day

13. What is your main type of exercise? *(tick one box only)*

☐ Taichi /Qigong /Leisure walking ☐ Brisk walking /Gymnastics /Folk dancing

☐ Jogging /Aerobic exercise ☐ Swimming
☐ 'Ball' games (including also billiards, bowling, golf, tennis, table tennis and badminton)
☐ Other (eg. mountain climbing, home exercises, and rope jumping)

14. About how many hours per week did you spent on these exercises? ___ hrs
15. In the past 12 months, how often did you sweat or have a much faster heartbeat because of exercise?

☐ Never or almost never } → *go to Question 17*
☐ <1 time / week
☐ 1-2 times/week
☐ 3-5 times/week
☐ Daily or almost every day

16. About how many hours per week did you do such vigorous activities? ____hrs
17. In the past 12 months, about how many hours per week did you do housework? ____hrs
18. In the past 12 months, during leisure time, about how many hours per week did you spend on sitting activities (e.g. watching TV or reading)? ___hrs
19. How many hours do you typically sleep per 24h day (incl. naps)? ____hrs

* Information can also be found at http://www.ckbiobank.org/about-the-study/study-design

Table S1: Physical activity types, MET values, codes and intensity categories*

Activity type	Intensity	MET	Codes*
Heavy manual work	Vigorous	6.5	11477
Manual work	Moderate	4.5	11476
Standing work	Moderate	3.2	Mean of 11600, 11610 and 11615
Sedentary work	Low	1.8	Mean of 11580, 11585, and 11590
Manual work in the farming season	Vigorous	6.3	Mean of 11145 and 11146
Semi-mechanized work in the farming season	Moderate	3.4	Mean of 11146 and 11147
Fully mechanized work in the farming season	Low	2.4	Mean of 11147 and 11170
Work outside the farming season	Low	2.0	11147
Walking	Moderate	4.0	17270
Bicycle	Vigorous	6.8	01011
Motorbike	Low	2.8	16030
Private or public transportation (such as bus, car, underground, and ferry)	Low	1.5	Mean of 16010, 16015, and 16016
Household activity	Low	2.7	Mean of 05030†, 05040†, 05035, 05055, 05070, 05090†, 05092†, 05184, 05197, and 05200
Tai-Chi/qigong/leisure walking	Moderate	3.4	Mean of 15670 and 17160
Jogging/aerobic exercise	Vigorous	7.6	Mean of 02000, 12020, and 12150
Ball games	Moderate	5.6	Mean of 15020†, 15030†, 15055, 15080, 15090, 15255, 15605†, 15610†, 15652, 15660, 15675†, 15676†, 15710†, and 15711†
Brisk walking/gymnastics/folk dancing	Moderate	4.4	Mean of 03025, 15300, and 17200
Swimming	Vigorous	7.2	Mean of 18230, 18240, and 18310
Other exercise, e.g. mountain walking, home exercise and rope jumping	Moderate	5.9	Mean of 01200, 02064, 04001, 04100, 15110†, 15120†, 15200, 15240, 15310, 15425†, 15430†, 15537, 15550‡, 15551‡, 15552‡, 15580, 15590, 15730, 15732‡, 15733‡, 15734‡, and 19030

MET: Metabolic equivalent of tasks.

* Based on the 2024 Adult Compendium of Physical Activities: A third update of the energy costs of human activities. Herrmann SD, et al. J Sport Health Sci. 2024 Jan;13(1):6-12.

† Assigned 1/2 weight in calculating the mean MET value because the connecting two items represent one type of activity.

‡ Assigned 1/3 weight in calculating the mean MET value because the connecting three items represent one type of activity.

Table S2: ICD-10 codes consolidated in phenome-wide investigation

Chapter	Consolidated ICD-10 codes	Description of the disease
I	A00-A09	Intestinal infectious diseases
I	A17-A19	Extra-pulmonary TB
II	C00-C14	Lip, oral cavity & pharynx cancer
II	C23-C24	Biliary cancer other than the liver
II	C40-C41	Malignant neoplasms of bone & articular cartilage
II	C70-C72	CNS cancer
II	C82-C88	Non-Hodgkin lymphoma
IV	E10-E14	Diabetes mellitus
V	F01-F03	Dementia
V	F10-F19	Mental and behavioural disorders due to psychoactive substance use
V	F32-F33	Major depressive disorder
VI	G30-G32	Other degenerative diseases of the nervous system
VI	G43-G44	Migraine and other headache syndromes
VII	H11, H13	Other disorders of conjunctiva
VII	H25, H26.9	Cataract
VII	H40-H42	Glaucoma
IX	I05-I09	Chronic rheumatic heart disease
IX	I30-I32	Diseases of pericardium
IX	I33, I38	Endocarditis
IX	I34-I37	Non-rheumatic heart valve disorders
IX	I47-I49	Cardiac arrhythmias
IX	I65-I66	Occlusion and stenosis of precerebral or cerebral arteries, not resulting in cerebral infarction
IX	I83, I85, I86	Varicose veins
X	J01-J06	Other acute upper respiratory infections (other than common cold)
X	J09-J11	Influenza
X	J12-J18	Pneumonia
X	J20-J22	Other acute lower respiratory infections
X	J30-J39	Other diseases of upper respiratory tract
X	J90-J91	Pleural effusion

Table S3: ICD-10 codes for diseases excluded in sensitivity analysis 5 (disease in the top 50 disability weights as defined by the 2013 GBD study*)

Chapter	ICD-10 codes	Description of the disease
I	A15-A16	Tuberculosis
I	B20-B24	HIV
II	C00-C97	Cancer
V	F00-F03	Dementia
V	F10	Alcohol use disorder
V	F11	Heroin and other opioid dependence
V	F14	Cocaine dependence
V	F15	Amphetamine dependence
V	F20-F29	Schizophrenia
V	F31	Bipolar disorder
V	F32-F33	Major depressive disorder
V	F40-F41	Anxiety disorders
V	F82	Motor impairment
VI	G20	Parkinson's disease
VI	G35	Multiple sclerosis
VI	G40-G41	Epilepsy
IX	I21	Acute myocardial infarction
IX	I60-I63	Stroke
X	J41-J44	Chronic obstructive pulmonary disease and other chronic respiratory diseases
XI	K60	Rectovaginal fistula
XIII	M00-M99	Musculoskeletal problems
XIV	N17-N19	End-stage renal disease
XIV	N82	Vesicovaginal fistula
XVIII	R51	Headache
XIX	S01-02, S04, S06-S08	Traumatic brain injury
XIX	S02, S12, S22, S32, S42, S52, S62, S72 S82, S92	Fracture
XIX	S21-S27	Severe chest injury
XIX	S32-34	Spinal cord lesion
XIX	T20-T32	Burns

* Based on Disability weights for the Global Burden of Disease 2013 study. Salomon J A, et al. The Lancet. Global Health, 2015, 3(11): e712-e723.

Table S4: Outcome classifications of aggregate endpoints

Disease	Aggregate endpoints			
	Diseases listed in WHO PA guidelines (18-64 years)	Diseases listed in WHO PA guidelines (over 65 years)	CKB PA-associated incidence	CKB PA-associated mortality
B18 Chronic viral hepatitis			Yes	Yes
B99 Other and unspecified infectious diseases			Yes	
C15 Malignant neoplasm of oesophagus	Critical	Critical		
C16 Malignant neoplasm of stomach	Critical	Critical		
C18 Malignant neoplasm of colon	Critical	Critical		Yes
C22 Malignant neoplasm of liver and intrahepatic bile ducts			Yes	Yes
C23-C24 Biliary cancer other than the liver			Yes	
C34 Malignant neoplasm of bronchus and lung	Critical	Critical	Yes	Yes
C50 Malignant neoplasm of breast	Critical	Critical	Yes	
C54 Malignant neoplasm of corpus uteri	Critical	Critical		
C64 Malignant neoplasm of kidney, except renal pelvis	Critical	Critical		
C67 Malignant neoplasm of bladder	Critical	Critical		
C77 Secondary and unspecified malignant neoplasm of lymph nodes			Yes	
D14 Benign neoplasm of middle ear and respiratory system			Yes	
D64 Other anaemias			Yes	
D69 Purpura and other haemorrhagic conditions			Yes	
E03 Other hypothyroidism			Yes	
E10-E14 Diabetes mellitus	Critical	Critical	Yes	Yes
E46 Unspecified protein-energy malnutrition			Yes	
E78 Disorders of lipoprotein metabolism and other lipidaemias			Yes	
E88 Other metabolic disorders			Yes	
F00 Dementia in Alzheimer disease	Critical	Critical		
F01-F03 Dementia	Important	Important	Yes	
F06 Other mental disorders due to brain damage and dysfunction and to physical disease			Yes	
F20 Schizophrenia			Yes	

F32-F33 Major depressive disorder	Critical	Critical		
F41 Other anxiety disorders	Critical	Critical		
G20 Parkinson disease			Yes	
G31 Other degenerative diseases of nervous system, not elsewhere classified			Yes	
G40 Epilepsy			Yes	
G45 Transient cerebral ischemic attacks and related syndromes			Yes	
G47 Sleep disorders	Important	Important	Yes	
H25, H26.9 Cataract			Yes	
H43 Disorders of vitreous body			Yes	
I05-I09 Chronic rheumatic heart disease			Yes	Yes
I10 Essential (primary) hypertension	Important	Important	Yes	
I20 Angina pectoris			Yes	
I21 Acute myocardial infarction			Yes	Yes
I25 Chronic ischemic heart disease	Critical	Critical	Yes	Yes
I27 Other pulmonary heart diseases			Yes	Yes
I46 Cardiac arrest			Yes	
I47-I49 Cardiac arrhythmias			Yes	
I50 Heart failure			Yes	
I51 Complications and ill-defined descriptions of heart disease			Yes	
I61 Intracerebral hemorrhage	Critical	Critical	Yes	Yes
I63 Cerebral infarction	Critical	Critical	Yes	Yes
I64 Stroke, not specified as hemorrhage or infarction	Critical	Critical	Yes	
I65-I66 Occlusion and stenosis of precerebral or cerebral arteries, not resulting in cerebral infarction			Yes	
I69 Sequelae of cerebrovascular disease			Yes	Yes
I70 Atherosclerosis			Yes	
J12-J18 Pneumonia			Yes	Yes
J42 Unspecified chronic bronchitis				Yes
J44 Other chronic obstructive pulmonary disease			Yes	Yes
J96 Respiratory failure, not elsewhere classified			Yes	

K29 Gastritis and duodenitis		Yes	
K30 Dyspepsia		Yes	
K74 Fibrosis and cirrhosis of liver		Yes	Yes
K76 Other diseases of liver		Yes	
K77 Liver disorders in diseases classified elsewhere		Yes	
K80 Cholelithiasis		Yes	
K81 Cholecystitis		Yes	
K85 Acute pancreatitis		Yes	
K92 Other diseases of digestive system		Yes	
M10 Gout		Yes	
M47 Spondylosis		Yes	
M53 Other dorsopathies, not elsewhere classified		Yes	
M80 Osteoporosis with pathological fracture	Critical		
M81 Osteoporosis without pathological fracture	Critical	Yes	
N03 Chronic nephritic syndrome		Yes	Yes
N18 Chronic kidney disease		Yes	
N19 Unspecified kidney failure		Yes	
N40 Hyperplasia of prostate		Yes	
R10 Abdominal and pelvic pain		Yes	
R33 Retention of urine		Yes	
R69 Unknown and unspecified causes of morbidity		Yes	
R73 Elevated blood glucose level		Yes	
R96 Other sudden death, cause unknown			Yes
R99 Other ill-defined and unspecified causes of mortality			Yes
W01 Fall on same level from slipping, tripping and stumbling	Critical		

Critical: an outcome that is critical to decision-making. Important: an outcome that is important, but not critical to decision-making.

Table S5: Estimated regression dilution ratio for physical activity

Baseline PA quintile group (MET-h/d)	No. of participants	Mean PA level (MET-h/d)		Ratio of the ranges estimate†
		baseline	second resurvey	
**Total PA**				
(i) 0.00-8.59	4,633	5.70	11.97	0.35
(ii) 8.60-13.99	5,269	11.35	14.72	
(iii) 14.00-21.24	4,943	17.39	17.52	
(iv) 21.25-31.89	4,916	26.16	21.13	
(V) 31.90-108.10	5,196	42.23	24.79	
Difference (V– i)		36.53	12.82	
**Occupational PA‡**				
(i) 0.00-0.19	267	0.00	6.40	0.36
(ii) 0.20-11.99	6,749	6.62	9.73	
(iii) 12.00-18.28	3,285	14.92	15.38	
(iv) 18.29-27.42	3,493	22.47	17.59	
(V) 27.43-100.00	3,793	38.00	19.89	
Difference (V– i)		38.00	13.49	
**Nonoccupational PA**				
(i) 0.00-3.73	4,774	2.01	8.51	0.39
(ii) 3.74-6.29	4,666	5.13	10.62	
(iii) 6.30-8.25	5,006	7.48	12.72	
(iv) 8.26-11.33	5,049	9.83	13.40	
(V) 11.34-86.80	5,462	15.23	13.64	
Difference (V– i)		13.22	5.13	
**Low-intensity PA**				
(i) 0.00-2.69	3,107	0.81	9.19	0.20
(ii) 2.70-6.16	6,479	4.42	10.32	
(iii) 6.17-9.06	4,969	7.83	10.60	
(iv) 9.07-13.49	4,794	11.16	11.28	
(V) 13.50-41.67	5,608	17.72	12.61	
Difference (V– i)		16.91	3.42	
**Moderate-to-vigorous intensity PA**				
(i) 0.00-0.04	5,832	0.00	5.40	0.27
(ii) 0.05-2.51	3,974	1.52	7.62	
(iii) 2.52-5.89	4,750	3.93	6.76	
(iv) 5.90-20.09	5,267	11.36	7.98	
(V) 20.10-105.53	5,134	34.88	14.8	
Difference (V– i)		34.88	9.40	

PA: physical activity. MET-h/d: metabolic equivalent of task per hour per day.

† The regression dilution ratios (RDRs) were calculated using the MacMahon's method, which uses the ratio of the ranges (top vs bottom quintile, defined by baseline physical activity) of the mean physical activity levels at first resurvey to the range of such measurements at baseline.

‡ The analysis of Occupational PA is restricted to participants with paid employment.

Table S6: Summary of number of mortality events with at least 100 events associated with physical activity by ICD-10 chapter

ICD-10 Chapter		No. of diseases	No. of events	No. of significant associations			
				Without FDR adjustment		With FDR adjustment	
				Negative	Positive	Negative	Positive
I	Infectious and parasitic	1	246	1	0	1	0
II	Neoplasms	21	15,720	6	0	3	0
III	Blood and immune-related	0	0	0	0	0	0
IV	Endocrine, nutritional and metabolic	1	373	1	0	1	0
V	Mental and behavioural	1	113	0	0	0	0
VI	Nerve-related	1	172	0	0	0	0
VII	Eye and adnexa	0	0	0	0	0	0
VIII	Ear and mastoid process	0	0	0	0	0	0
IX	Circulatory	12	17,231	7	0	7	0
X	Respiratory	3	2,351	3	0	3	0
XI	Digestive	2	427	1	0	1	0
XII	Skin and subcutaneous tissue	0	0	0	0	0	0
XIII	Musculoskeletal	0	0	0	0	0	0
XIV	Genitourinary	1	291	1	0	1	0
XV	Pregnancy-related	0	0	0	0	0	0
XVIII	Other symptoms, signs and abnormal findings	2	887	2	0	2	0
XIX	Injury, poisoning and other external causes	0	0	0	0	0	0
XX	External causes	8	1,509	0	0	0	0
**Total**		**53**	**39,320**	**22**	**0**	**19**	**0**

Table S7: Adjusted HRs for specific diseases showing significant associations with non-occupational physical activity after FDR adjustment in rural and urban regions

Disease	Urban			Rural		
	No. event	HR (95%CI)	Sig. FDR P	No. event	HR (95%CI)	Sig. FDR P
E10-E14 Diabetes mellitus	8,267	0.85 (0.81-0.90)	Yes	10,181	0.86 (0.82-0.91)	Yes
I21 Acute myocardial infarction	2,530	0.72 (0.65-0.79)	Yes	4,404	0.83 (0.76-0.90)	
I25 Chronic ischemic heart disease	20,337	0.85 (0.83-0.88)	Yes	17,634	0.93 (0.90-0.97)	
I27 Other pulmonary heart diseases	226	0.43 (0.30-0.61)	Yes	2,089	0.90 (0.80-1.01)	
I46 Cardiac arrest	290	0.53 (0.40-0.72)	Yes	281	1.18 (0.85-1.65)	
I50 Heart failure	1,294	0.63 (0.56-0.72)	Yes	2,224	0.90 (0.81-1.00)	
I61 Intracerebral hemorrhage	2,794	0.77 (0.70-0.84)	Yes	7,607	0.85 (0.80-0.91)	Yes
I63 Cerebral infarction	23,837	0.88 (0.86-0.91)	Yes	22,716	0.88 (0.85-0.91)	Yes
I64 Stroke, not specified as hemorrhage or infarction	1,071	0.74 (0.64-0.85)	Yes	732	0.76 (0.61-0.95)	
I67 Other cerebrovascular diseases	12,843	0.91 (0.88-0.94)	Yes	19,131	0.98 (0.95-1.02)	
I69 Sequelae of cerebrovascular disease	1,893	0.69 (0.62-0.77)	Yes	3,739	0.96 (0.87-1.05)	
I83, I85, I86 Varicose veins	1,200	1.42 (1.25-1.62)	Yes	2,186	1.06 (0.93-1.22)	
J12-J18 Pneumonia	12,130	0.82 (0.78-0.85)	Yes	16,140	1.00 (0.96-1.04)	
J44 Other chronic obstructive pulmonary disease	2,027	0.65 (0.59-0.73)	Yes	6,968	0.89 (0.83-0.96)	
K40 Inguinal hernia	1,621	1.39 (1.23-1.56)	Yes	2,568	1.08 (0.93-1.25)	
K80 Cholelithiasis	3,335	0.79 (0.73-0.85)	Yes	8,210	0.85 (0.81-0.90)	Yes
K81 Cholecystitis	2,011	0.69 (0.62-0.76)	Yes	4,574	1.00 (0.94-1.08)	
M10 Gout	658	0.66 (0.54-0.80)	Yes	657	1.08 (0.81-1.44)	
M47 Spondylosis	3,085	0.76 (0.70-0.82)	Yes	14,036	0.94 (0.91-0.98)	
N03 Chronic nephritic syndrome	359	0.44 (0.33-0.59)	Yes	562	0.90 (0.70-1.15)	
N18 Chronic kidney disease	927	0.47 (0.40-0.55)	Yes	940	0.86 (0.70-1.05)	
N19 Unspecified kidney failure	910	0.56 (0.48-0.65)	Yes	859	1.27 (1.04-1.53)	
R69 Unknown and unspecified causes of morbidity	13,828	0.82 (0.79-0.85)	Yes	7,472	0.97 (0.91-1.03)	
R99 Other ill-defined and unspecified causes of mortality	564	0.65 (0.53-0.78)	Yes	136	0.54 (0.32-0.92)	

HR: hazard ratio;

HRs were stratified by age at risk (5-year groups), sex and ten study areas, and were adjusted for education, drinking status, smoking status and occupational physical activity.

Table S8: Adjusted HRs for specific diseases showing significant associations with occupational physical activity after FDR adjustment in rural and urban regions

Disease	Urban			Rural		
	No. event	HR (95%CI)	Sig. FDR P	No. event	HR (95%CI)	Sig. FDR P
A00-A09 Intestinal infectious diseases	717	0.57 (0.49-0.66)		5,161	0.72 (0.68-0.77)	Yes
E10-E14 Diabetes mellitus	3,900	0.83 (0.78-0.88)		8,285	0.65 (0.62-0.68)	Yes
G40 Epilepsy	137	0.47 (0.35-0.63)		368	0.29 (0.23-0.37)	Yes
G45 Transient cerebral ischemic attacks and related syndromes	1,958	0.78 (0.71-0.85)		3,548	0.65 (0.59-0.70)	Yes
I05-I09 Chronic rheumatic heart disease	63	invalid estimate		363	0.42 (0.32-0.55)	Yes
I10 Essential (primary) hypertension	2,094	1.18 (1.08-1.30)		4,385	0.72 (0.67-0.77)	Yes
I21 Acute myocardial infarction	672	1.40 (1.20-1.63)		3,114	0.56 (0.51-0.61)	Yes
I25 Chronic ischemic heart disease	6,287	0.64 (0.61-0.68)		13,080	0.68 (0.65-0.71)	Yes
I51 Complications and ill-defined descriptions of heart disease	302	0.84 (0.68-1.04)		1,048	0.55 (0.47-0.65)	Yes
I61 Intracerebral hemorrhage	974	0.85 (0.76-0.95)		5,688	0.61 (0.57-0.65)	Yes
I63 Cerebral infarction	7,093	0.76 (0.72-0.79)		17,781	0.79 (0.76-0.82)	Yes
I69 Sequelae of cerebrovascular disease	519	0.39 (0.33-0.46)		2,760	0.60 (0.54-0.66)	Yes
K59 Other functional intestinal disorders	104	invalid estimate		282	0.40 (0.29-0.55)	Yes
K80 Cholelithiasis	1,485	0.83 (0.75-0.91)		6,755	0.75 (0.71-0.79)	Yes
K92 Other diseases of digestive system	782	0.77 (0.68-0.87)		1,950	0.59 (0.53-0.65)	Yes
M10 Gout	223	invalid estimate		519	0.40 (0.33-0.48)	Yes
N40 Hyperplasia of prostate	474	invalid estimate		1,683	0.64 (0.57-0.71)	Yes
R69 Unknown and unspecified causes of morbidity	7,675	0.66 (0.64-0.69)		6,902	0.67 (0.63-0.71)	Yes
S72 Fracture of femur	377	0.60 (0.50-0.73)		1,258	0.50 (0.44-0.57)	Yes

HR: hazard ratio;

HRs were stratified by age at risk (5-year groups), sex and ten study areas, and were adjusted for education, drinking status, smoking status and non-occupational physical activity.

Table S9: Multivariable-adjusted HRs (95%CIs) and PAR%(95%CIs) for physical inactivity in relation to specific diseases

	No. events	HR (95%CI)	PAR% (95%CI)
**Incidence**			
F20 Schizophrenia	614	1.74 (1.45-2.10)	18.7 (12.2-25.3)
F01-F03 Dementia	309	1.60 (1.24-2.06)	15.6 (7.0-24.7)
G40 Epilepsy	818	1.55 (1.32-1.83)	14.6 (9.0-20.4)
N18 Chronic kidney disease	1,867	1.50 (1.35-1.66)	13.3 (9.7-17.0)
N19 Unspecified kidney failure	1,769	1.49 (1.34-1.66)	13.2 (9.5-17.0)
I05-I09 Chronic rheumatic heart disease	669	1.44 (1.21-1.73)	12.1 (6.1-18.4)
D69 Purpura and other haemorrhagic conditions	815	1.37 (1.16-1.62)	10.3 (4.7-16.1)
I27 Other pulmonary heart diseases	2,315	1.37 (1.25-1.51)	10.3 (7.1-13.6)
E79 Disorders of purine and pyrimidine metabolism	327	1.37 (1.06-1.77)	10.2 (1.8-19.2)
B18 Chronic viral hepatitis	888	1.34 (1.14-1.58)	9.5 (4.2-15.2)
E46 Unspecified protein-energy malnutrition	253	1.32 (0.98-1.79)	9.0 (-0.8-19.6)
I46 Cardiac arrest	571	1.32 (1.08-1.60)	8.9 (2.5-15.6)
I51 Complications and ill-defined descriptions of heart disease	2,150	1.30 (1.18-1.43)	8.5 (5.3-11.8)
K76 Other diseases of liver	1,978	1.28 (1.15-1.43)	8.0 (4.5-11.7)
G20 Parkinson disease	899	1.28 (1.10-1.48)	7.9 (2.9-13.0)
I70 Atherosclerosis	1,207	1.27 (1.11-1.45)	7.8 (3.4-12.3)
K74 Fibrosis and cirrhosis of liver	1,898	1.26 (1.14-1.41)	7.6 (4.1-11.2)
I20 Angina pectoris	2,438	1.26 (1.15-1.39)	7.6 (4.5-10.7)
G47 Sleep disorders	445	1.26 (1.00-1.58)	7.5 (0.1-15.3)
D64 Other anaemias	1,778	1.25 (1.12-1.40)	7.3 (3.6-11.1)
**All-cause**	331,971	1.10 (1.10-1.11)	3.1 (2.9-3.4)
**Mortality**			
I05-I09 Chronic rheumatic heart disease	171	2.19 (1.55-3.09)	26.9 (14.5-39.3)
R96 Other sudden death, cause unknown	205	2.09 (1.50-2.91)	25.3 (13.4-37.2)
I27 Other pulmonary heart diseases	162	1.73 (1.20-2.49)	18.4 (5.7-31.6)
J42 Unspecified chronic bronchitis	464	1.70 (1.37-2.11)	17.8 (10.3-25.6)
J12-J18 Pneumonia	654	1.59 (1.32-1.93)	15.5 (9.0-22.3)
E10-E14 Diabetes mellitus	373	1.55 (1.23-1.96)	14.6 (6.6-22.9)
B18 Chronic viral hepatitis	246	1.53 (1.14-2.06)	14.1 (4.0-24.7)
I69 Sequelae of cerebrovascular disease	1,749	1.51 (1.36-1.69)	13.7 (9.9-17.6)
J44 Other chronic obstructive pulmonary disease	1,233	1.45 (1.28-1.65)	12.3 (7.9-16.8)
I25 Chronic ischemic heart disease	2,510	1.45 (1.32-1.59)	12.2 (9.0-15.4)
I63 Cerebral infarction	2,004	1.43 (1.29-1.59)	11.8 (8.3-15.4)
R99 Other ill-defined and unspecified causes of mortality	682	1.42 (1.20-1.70)	11.6 (5.7-17.8)
K74 Fibrosis and cirrhosis of liver	320	1.34 (1.04-1.73)	9.6 (1.3-18.4)
I61 Intracerebral hemorrhage	5,507	1.31 (1.23-1.39)	8.7 (6.7-10.8)
N03 Chronic nephritic syndrome	291	1.30 (1.00-1.69)	8.5 (-0.0-17.6)
I21 Acute myocardial infarction	4,203	1.28 (1.19-1.37)	7.9 (5.5-10.3)
C22 Malignant neoplasm of liver and intrahepatic bile ducts	2,448	1.24 (1.13-1.36)	7.0 (3.9-10.1)
C34 Malignant neoplasm of bronchus and lung	4,547	1.13 (1.06-1.21)	3.9 (1.7-6.2)
**All-cause**	55,052	1.38 (1.35-1.40)	10.5 (9.8-11.1)

HRs were stratified by age at risk (5-year groups), sex and ten study areas and adjusted for education, drinking status and smoking status. PAR% for FDR-adjusted significant diseases were calculated, and the top 20 were listed.

PAR%: Population Attributable Risk Percent. HR: hazard ratio. CI: confidence interval.

Figure S1: Flow diagram of study participants

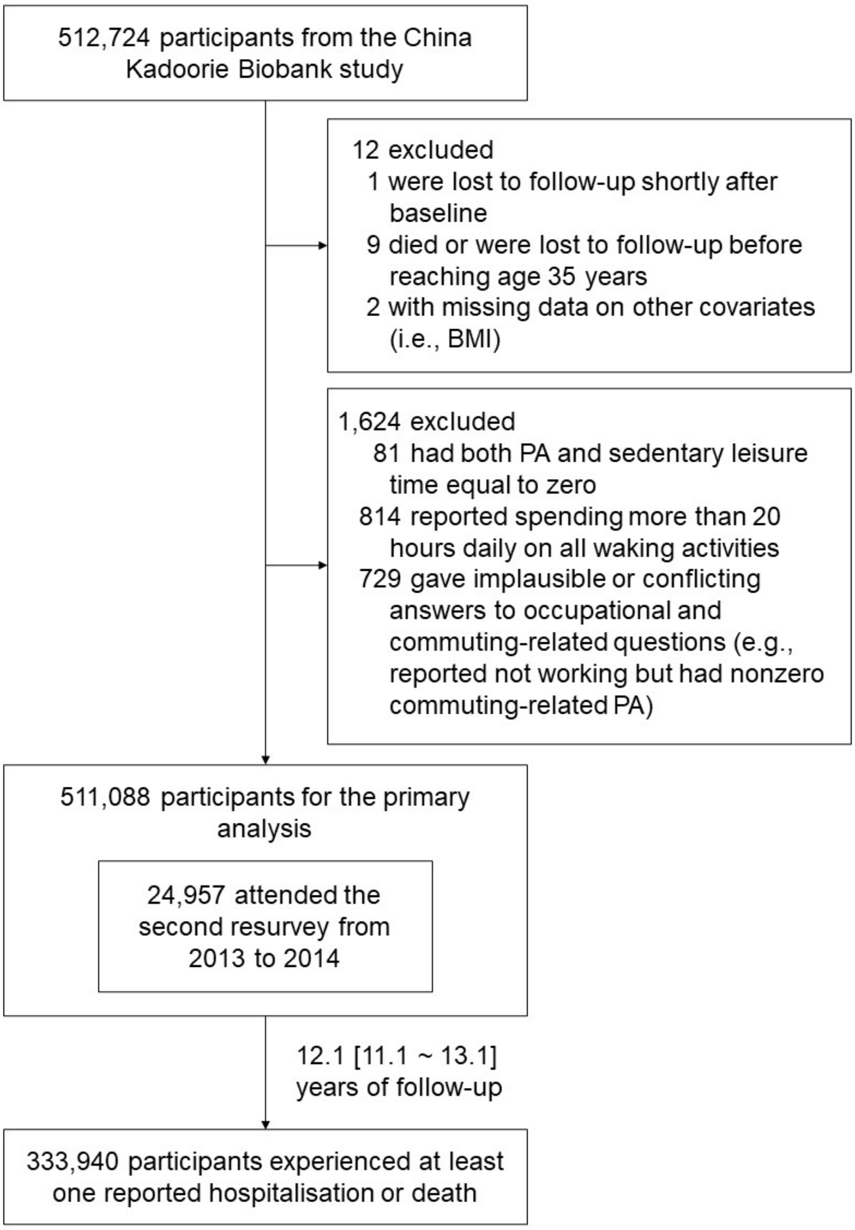

Figure S2: Adjusted HRs for ICD-10 chapter-specific incidence associated with physical activity

Chapter	No.event	Q5 v.s. Q1	HR (95%CI)	Sig.FDR P
I Infectious and parasitic	18,210		0.91 (0.87-0.95)	Yes
II Neoplasms	48,316		0.84 (0.82-0.86)	Yes
III Blood and immune-related	4,252		0.74 (0.68-0.80)	Yes
IV Endocrine, nutritional and metabolic	41,728		0.67 (0.65-0.69)	Yes
V Mental and behavioural	5,146		0.61 (0.57-0.66)	Yes
VI Nerve-related	19,574		0.84 (0.80-0.88)	Yes
VII Eye and adnexa	26,207		0.90 (0.86-0.94)	Yes
VIII Ear and mastoid process	7,129		0.94 (0.88-1.01)	
IX Circulatory	148,757		0.77 (0.76-0.79)	Yes
X Respiratory	70,273		0.84 (0.82-0.86)	Yes
XI Digestive	74,232		0.90 (0.88-0.91)	Yes
XII Skin and subcutaneous tissue	4,450		0.89 (0.82-0.96)	Yes
XIII Musculoskeletal	55,180		0.95 (0.92-0.97)	Yes
XIV Genitourinary	40,884		0.91 (0.89-0.94)	Yes
XV Pregnancy-related	1,783		1.14 (1.00-1.29)	
XVIII Other symptoms, signs and abnormal findings	43,764		0.83 (0.81-0.85)	Yes
XIX Injury, poisoning and other external causes	29,119		1.06 (1.03-1.09)	Yes
XX External causes	3,973		0.97 (0.90-1.05)	
Any	331,971		0.86 (0.85-0.87)	Yes

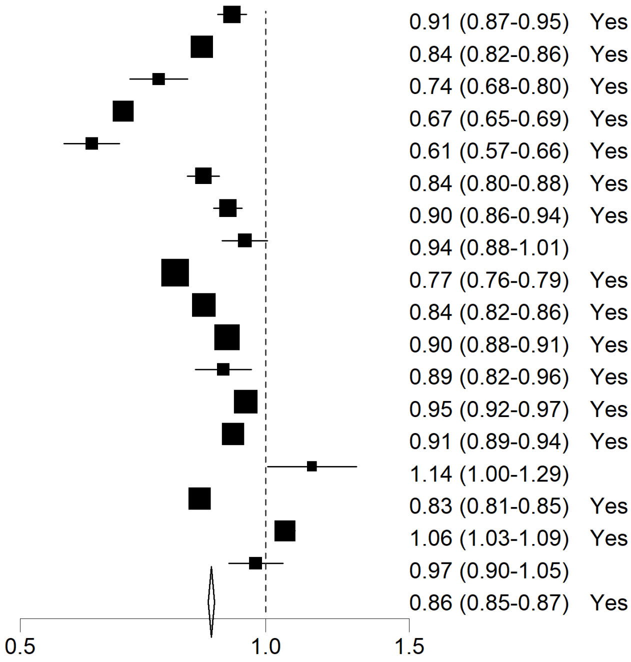

PA: physical activity; HR: hazard ratio; Q_5: highest quintile; Q_1: lowest quintile.

The x-axis is on a log scale. The HRs for the highest quintile group compared to the lowest quintile group are presented in the figure. HRs were stratified by age at risk (5-year groups), sex and ten study areas, and were adjusted for education, drinking status and smoking status.

Figure S3: Adjusted HRs for incidence of specific types of infectious and parasitic diseases associated with physical activity

Chapter	No.event	HR (95%CI)	Sig.FDR P
**I Infectious and parasitic**			
B18 Chronic viral hepatitis	888	0.66 (0.55-0.78)	Yes
B99 Other and unspecified infectious diseases	947	0.68 (0.60-0.79)	Yes
A41 Other sepsis	323	0.74 (0.50-1.10)	
A46 Erysipelas	351	0.76 (0.54-1.05)	
A15 Respiratory tuberculosis, bacteriologically and histologically confirmed	530	0.79 (0.61-1.02)	
B19 Unspecified viral hepatitis	473	0.86 (0.69-1.07)	
B17 Other acute viral hepatitis	119	0.88 (0.55-1.40)	
B24 Unspecified human immunodeficiency virus [HIV] disease	105	0.90 (0.52-1.55)	
A00-A09 Intestinal infectious diseases	8,449	0.92 (0.87-0.98)	
A16 Respiratory tuberculosis, not confirmed bacteriologically or histologically	1,731	0.93 (0.81-1.06)	
A17-A19 Extra-pulmonary TB	523	0.96 (0.76-1.21)	
B02 Zoster [herpes zoster]	2,816	1.12 (1.00-1.25)	
B08 Other viral infections characterized by skin and mucous membrane lesions, not elsewhere classified	114	1.21 (0.74-1.96)	
Other	1,185	1.05 (0.91-1.23)	
**Any**	**18,210**	**0.91 (0.87-0.95)**	**Yes**

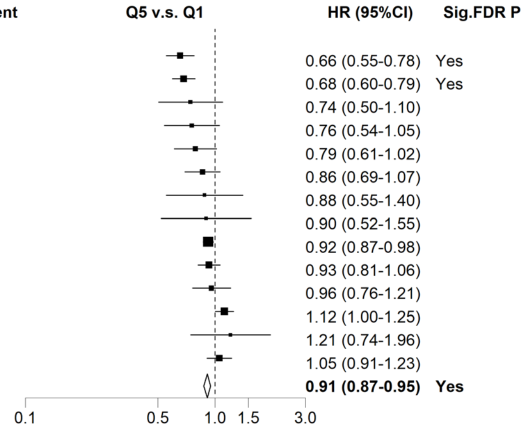

PA: physical activity. HR: hazard ratio.

The x-axis is on a log scale. HRs were stratified by age at risk (5-year groups), sex and ten study areas, and were adjusted for education, drinking status and smoking status.

Figure S4: Adjusted HRs for incidence of specific types of neoplasms associated with physical activity

Chapter	No.event	HR (95%CI)	Sig.FDR P
**II Neoplasms**			
D14 Benign neoplasm of middle ear and respiratory system	165	0.44 (0.28-0.70)	Yes
C77 Secondary and unspecified malignant neoplasm of lymph nodes	245	0.51 (0.34-0.77)	Yes
D11 Benign neoplasm of major salivary glands	212	0.55 (0.37-0.80)	
D31 Benign neoplasm of eye and adnexa	524	0.58 (0.39-0.87)	
D06 Carcinoma in situ of cervix uteri	210	0.59 (0.42-0.82)	
C55 Malignant neoplasm of uterus, part unspecified	372	0.62 (0.43-0.90)	
D00 Carcinoma in situ of oral cavity, oesophagus and stomach	119	0.63 (0.37-1.05)	
D39 Neoplasm of uncertain or unknown behaviour of female genital organs	312	0.64 (0.48-0.87)	
D41 Neoplasm of uncertain or unknown behaviour of urinary organs	272	0.66 (0.46-0.94)	
C23-C24 Biliary cancer other than the liver	781	0.66 (0.53-0.82)	Yes
D12 Benign neoplasm of colon, rectum, anus and anal canal	424	0.68 (0.50-0.91)	
C79 Secondary malignant neoplasm of other and unspecified sites	390	0.68 (0.50-0.92)	
C78 Secondary malignant neoplasm of respiratory and digestive organs	358	0.69 (0.51-0.93)	
C26 Malignant neoplasm of other and ill-defined digestive organs	205	0.70 (0.46-1.08)	
D18 Haemangioma and lymphangioma, any site	611	0.71 (0.58-0.87)	
C61 Malignant neoplasm of prostate	567	0.71 (0.54-0.94)	
C44 Other malignant neoplasms of skin	240	0.73 (0.50-1.06)	
D43 Neoplasm of uncertain or unknown behaviour of brain and central nervous system	301	0.74 (0.54-1.02)	
C64 Malignant neoplasm of kidney, except renal pelvis	489	0.74 (0.57-0.96)	
C22 Malignant neoplasm of liver and intrahepatic bile ducts	3,364	0.75 (0.68-0.82)	Yes
D32 Benign neoplasm of meninges	381	0.77 (0.59-1.01)	
C70-72 CNS cancer	646	0.79 (0.64-0.96)	
C96 Other and unspecified malignant neoplasms of lymphoid, haematopoietic and related tissue	134	0.80 (0.46-1.38)	
C18 Malignant neoplasm of colon	2,175	0.80 (0.70-0.92)	
D17 Benign lipomatous neoplasm	1,359	0.81 (0.71-0.92)	
D40 Neoplasm of uncertain or unknown behaviour of male genital organs	100	0.82 (0.43-1.56)	
C50 Malignant neoplasm of breast	2,838	0.82 (0.73-0.92)	Yes
C32 Malignant neoplasm of larynx	236	0.82 (0.56-1.21)	
C73 Malignant neoplasm of thyroid gland	734	0.83 (0.68-1.00)	
C54 Malignant neoplasm of corpus uteri	606	0.83 (0.66-1.04)	
C57 Malignant neoplasm of other and unspecified female genital organs	178	0.83 (0.53-1.30)	
C34 Malignant neoplasm of bronchus and lung	7,041	0.85 (0.79-0.91)	Yes
D44 Neoplasm of uncertain or unknown behaviour of endocrine glands	907	0.86 (0.71-1.02)	
C00-C14 Lip, oral cavity & pharynx cancer	807	0.86 (0.71-1.04)	
C80 Malignant neoplasm, without specification of site	3,305	0.86 (0.79-0.94)	
C82-C88 Non-Hodgkin lymphoma	942	0.87 (0.73-1.03)	
D26 Other benign neoplasms of uterus	1,106	0.88 (0.78-0.99)	
C15 Malignant neoplasm of oesophagus	2,722	0.88 (0.79-0.98)	
C40-C41 Malignant neoplasms of bone & articular cartilage	372	0.88 (0.67-1.17)	
D37 Neoplasm of uncertain or unknown behaviour of oral cavity and digestive organs	1,078	0.89 (0.77-1.02)	
D07 Carcinoma in situ of other and unspecified genital organs	121	0.89 (0.50-1.60)	
D24 Benign neoplasm of breast	806	0.90 (0.76-1.06)	
C16 Malignant neoplasm of stomach	3,964	0.91 (0.84-0.99)	
D34 Benign neoplasm of thyroid gland	767	0.92 (0.76-1.10)	
C53 Malignant neoplasm of cervix uteri	1,184	0.93 (0.79-1.08)	
D23 Other benign neoplasms of skin	263	0.93 (0.67-1.28)	
D48 Neoplasm of uncertain or unknown behaviour of other and unspecified sites	1,395	0.94 (0.81-1.09)	
C49 Malignant neoplasm of other connective and soft tissue	196	0.94 (0.65-1.37)	
D38 Neoplasm of uncertain or unknown behaviour of middle ear and respiratory and intrathoracic organs	849	0.95 (0.80-1.13)	
C20 Malignant neoplasm of rectum	2,087	0.95 (0.84-1.08)	
C19 Malignant neoplasm of rectosigmoid junction	106	0.95 (0.58-1.57)	
D35 Benign neoplasm of other and unspecified endocrine glands	260	0.96 (0.72-1.28)	
D25 Leiomyoma of uterus	4,917	0.97 (0.91-1.03)	
C67 Malignant neoplasm of bladder	628	0.98 (0.77-1.25)	
C76 Malignant neoplasm of other and ill-defined sites	347	1.03 (0.77-1.38)	
C17 Malignant neoplasm of small intestine	183	1.04 (0.70-1.54)	
C95 Leukaemia of unspecified cell type	498	1.06 (0.84-1.34)	
D01 Carcinoma in situ of other and unspecified digestive organs	99	1.07 (0.64-1.77)	
C56 Malignant neoplasm of ovary	540	1.08 (0.84-1.39)	
C25 Malignant neoplasm of pancreas	995	1.09 (0.92-1.29)	
D36 Benign neoplasm of other and unspecified sites	587	1.10 (0.90-1.33)	
D02 Carcinoma in situ of middle ear and respiratory system	108	1.11 (0.65-1.90)	
D21 Other benign neoplasms of connective and other soft tissue	185	1.12 (0.80-1.57)	
D27 Benign neoplasm of ovary	417	1.13 (0.89-1.43)	
D16 Benign neoplasm of bone and articular cartilage	227	1.15 (0.84-1.56)	
D13 Benign neoplasm of other and ill-defined parts of digestive system	448	1.16 (0.90-1.49)	
D30 Benign neoplasm of urinary organs	157	1.19 (0.77-1.85)	
C48 Malignant neoplasm of retroperitoneum and peritoneum	98	1.21 (0.73-2.02)	
D46 Myelodysplastic syndromes	130	1.21 (0.76-1.95)	
D33 Benign neoplasm of brain and other parts of central nervous system	216	1.22 (0.91-1.65)	
C92 Myeloid leukaemia	229	1.29 (0.93-1.79)	
C91 Lymphoid leukaemia	190	1.39 (0.97-1.98)	
C90 Multiple myeloma and malignant plasma cell neoplasms	308	1.59 (1.19-2.12)	
Other	1,584	0.74 (0.64-0.86)	Yes
**Any**	**48,316**	**0.84 (0.82-0.86)**	**Yes**

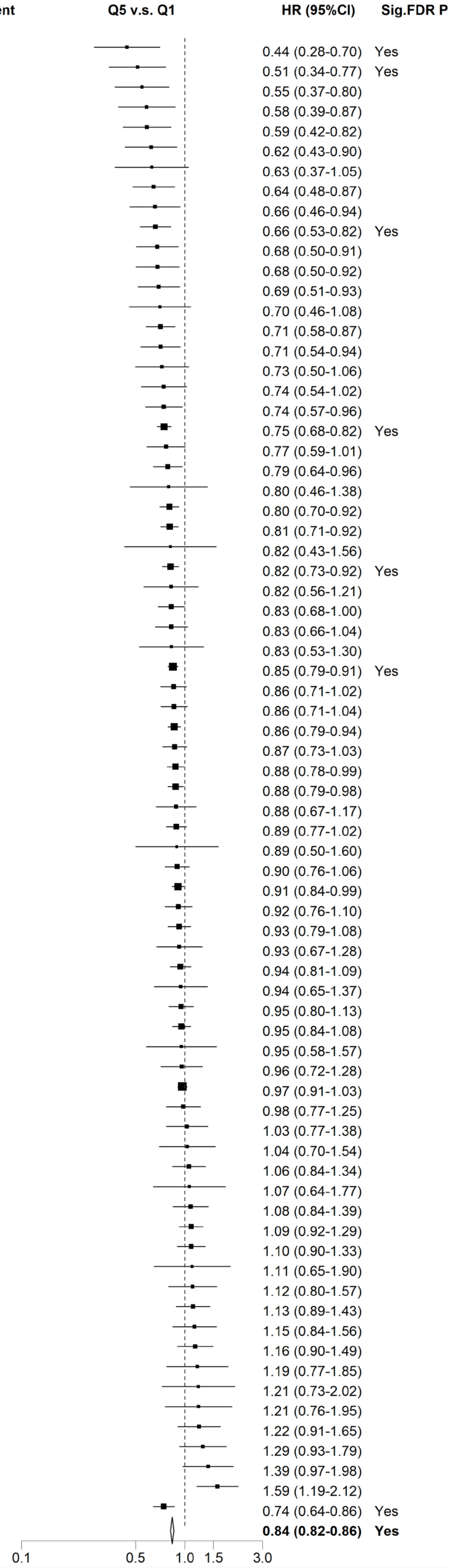

PA: physical activity. HR: hazard ratio.

The x-axis is on a log scale. HRs were stratified by age at risk (5-year groups), sex and ten study areas, and were adjusted for education, drinking status and smoking status.

Figure S5: Adjusted HRs for incidence of specific types of blood and immune-related diseases associated with physical activity

Chapter	No.event	Q5 v.s. Q1	HR (95%CI)	Sig.FDR P
**III Blood and immune-related**				
D69 Purpura and other haemorrhagic conditions	815		0.60 (0.49-0.73)	Yes
D53 Other nutritional anaemias	238		0.69 (0.52-0.91)	
D64 Other anaemias	1,778		0.72 (0.63-0.82)	Yes
D86 Sarcoidosis	194		0.79 (0.56-1.13)	
D50 Iron deficiency anaemia	560		0.80 (0.64-1.01)	
D61 Other aplastic anaemias	297		0.84 (0.62-1.15)	
D70 Agranulocytosis	327		1.16 (0.89-1.51)	
Other	499		0.62 (0.49-0.79)	Yes
**Any**	**4,252**		**0.74 (0.68-0.80)**	**Yes**

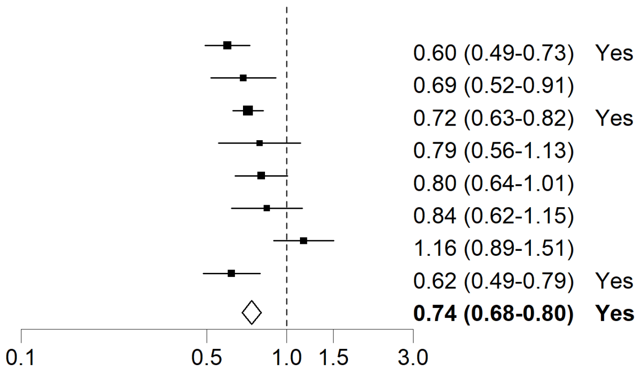

PA: physical activity. HR: hazard ratio.

The x-axis is on a log scale. HRs were stratified by age at risk (5-year groups), sex and ten study areas, and were adjusted for education, drinking status and smoking status.

Figure S6: Adjusted HRs for incidence of specific types of endocrine, nutritional and metabolic diseases associated with physical activity

Chapter	No.event	HR (95%CI)	Sig.FDR P
**IV Endocrine, nutritional and metabolic**			
E88 Other metabolic disorders	461	0.48 (0.35-0.65)	Yes
E46 Unspecified protein-energy malnutrition	253	0.53 (0.38-0.74)	Yes
E79 Disorders of purine and pyrimidine metabolism	327	0.54 (0.39-0.75)	Yes
E03 Other hypothyroidism	318	0.55 (0.39-0.75)	Yes
E10-E14 Diabetes mellitus	18,448	0.76 (0.74-0.79)	Yes
E78 Disorders of lipoprotein metabolism and other lipidaemias	2,839	0.78 (0.70-0.87)	Yes
E16 Other disorders of pancreatic internal secretion	448	0.80 (0.59-1.08)	
E87 Other disorders of fluid, electrolyte and acid-base balance	2,082	0.84 (0.75-0.95)	
E83 Disorders of mineral metabolism	108	0.90 (0.52-1.56)	
E07 Other disorders of thyroid	296	0.90 (0.69-1.19)	
E06 Thyroiditis	296	0.99 (0.74-1.33)	
E04 Other nontoxic goitre	2,244	0.99 (0.89-1.10)	
E05 Thyrotoxicosis [hyperthyroidism]	771	1.13 (0.94-1.35)	
Other	420	0.59 (0.44-0.79)	Yes
**Any**	**41,728**	**0.67 (0.65-0.69)**	**Yes**

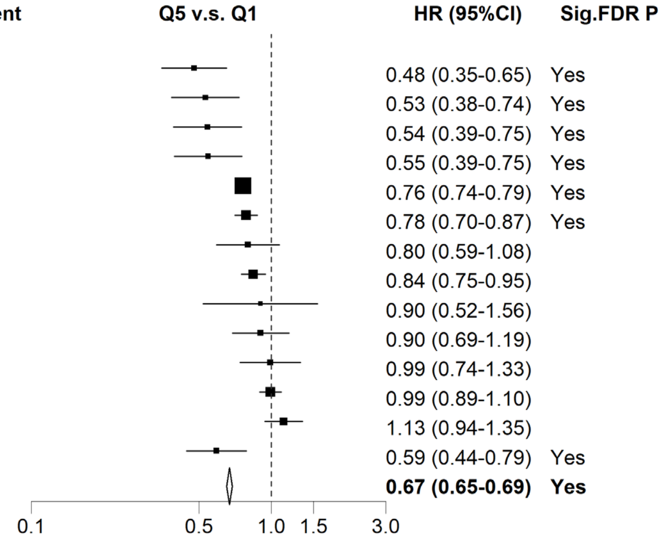

PA: physical activity. HR: hazard ratio.

The x-axis is on a log scale. HRs were stratified by age at risk (5-year groups), sex and ten study areas, and were adjusted for education, drinking status and smoking status.

Figure S7: Adjusted HRs for incidence of specific types of mental and behavioural disorders associated with physical activity

Chapter	No.event	HR (95%CI)	Sig.FDR P
**V Mental and behavioural**			
F06 Other mental disorders due to brain damage and dysfunction and to physical disease	168	0.31 (0.19-0.52)	Yes
F20 Schizophrenia	614	0.42 (0.34-0.52)	Yes
F01-F03 Dementia	309	0.47 (0.31-0.72)	Yes
F07 Personality and behavioural disorders due to brain disease, damage and dysfunction	127	0.69 (0.46-1.03)	
F45 Somatoform disorders	711	0.72 (0.58-0.88)	
F41 Other anxiety disorders	579	0.72 (0.57-0.91)	
F99 Mental disorder, not otherwise specified	678	0.72 (0.56-0.92)	
F48 Other neurotic disorders	605	0.76 (0.60-0.96)	
F32-F33 Major depressive disorder	792	0.81 (0.67-0.98)	
F44 Dissociative [conversion] disorders	191	0.85 (0.63-1.16)	
F00 Dementia in Alzheimer disease	159	1.08 (0.62-1.87)	
Other	319	0.69 (0.51-0.92)	
**Any**	**5,146**	**0.61 (0.57-0.66)**	**Yes**

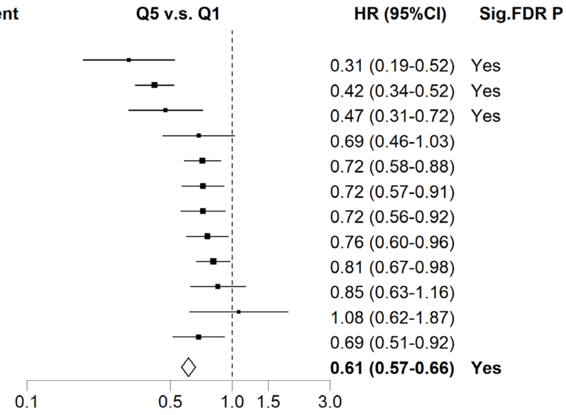

PA: physical activity. HR: hazard ratio.

The x-axis is on a log scale. HRs were stratified by age at risk (5-year groups), sex and ten study areas, and were adjusted for education, drinking status and smoking status.

Figure S8: Adjusted HRs for incidence of specific types of neurological diseases associated with physical activity

Chapter	No.event	HR (95%CI)	Sig.FDR P
**VI Nerve-related**			
G40 Epilepsy	818	0.51 (0.42-0.63)	Yes
G47 Sleep disorders	445	0.57 (0.43-0.75)	Yes
G31 Other degenerative diseases of nervous system, not elsewhere classified	828	0.58 (0.45-0.75)	Yes
G20 Parkinson disease	899	0.70 (0.55-0.88)	Yes
G93 Other disorders of brain	305	0.70 (0.50-0.99)	
G51 Facial nerve disorders	1,554	0.82 (0.70-0.94)	
G56 Mononeuropathies of upper limb	225	0.82 (0.61-1.11)	
G43-G44 Migraine and other headache syndromes	2,036	0.85 (0.75-0.96)	
G30-G32 Other degenerative diseases of the nervous system	121	0.88 (0.45-1.71)	
G90 Disorders of autonomic nervous system	198	0.89 (0.60-1.33)	
G45 Transient cerebral ischaemic attacks and related syndromes	10,579	0.90 (0.84-0.96)	Yes
G50 Disorders of trigeminal nerve	307	0.92 (0.67-1.26)	
G58 Other mononeuropathies	202	1.10 (0.75-1.61)	
G62 Other polyneuropathies	522	1.10 (0.87-1.41)	
G91 Hydrocephalus	171	1.21 (0.82-1.78)	
G70 Myasthenia gravis and other myoneural disorders	110	1.76 (1.05-2.95)	
G04 Encephalitis, myelitis and encephalomyelitis	133	1.92 (1.28-2.88)	
Other	984	0.83 (0.70-0.99)	
**Any**	**19,574**	**0.84 (0.80-0.88)**	**Yes**

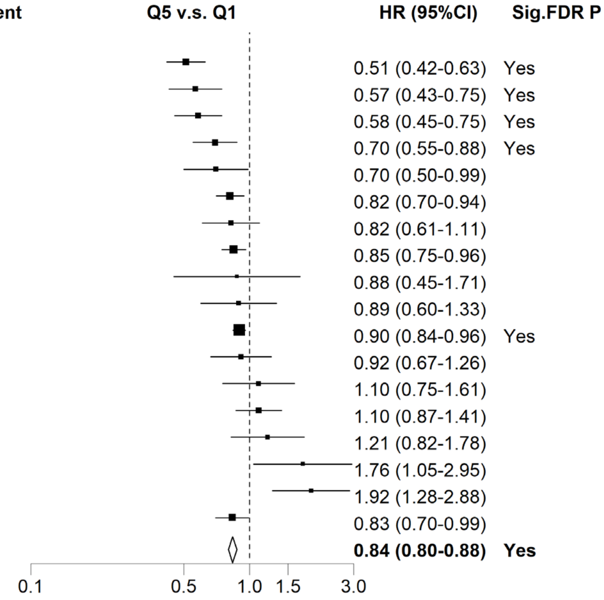

PA: physical activity. HR: hazard ratio.

The x-axis is on a log scale. HRs were stratified by age at risk (5-year groups), sex and ten study areas, and were adjusted for education, drinking status and smoking status.

Figure S9: Adjusted HRs for incidence of specific types of eye and adnexa diseases associated with physical activity

Chapter	No.event	HR (95%CI)	Sig.FDR P
**VII Eye and adnexa**			
H49 Paralytic strabismus	146	0.48 (0.24-0.96)	
H43 Disorders of vitreous body	492	0.57 (0.43-0.76)	Yes
H46 Optic neuritis	146	0.72 (0.45-1.14)	
H10 Conjunctivitis	201	0.73 (0.50-1.05)	
H20 Iridocyclitis	212	0.81 (0.53-1.22)	
H34 Retinal vascular occlusions	384	0.84 (0.60-1.16)	
H33 Retinal detachments and breaks	763	0.86 (0.71-1.04)	
H35 Other retinal disorders	584	0.87 (0.68-1.11)	
H40-H42 Glaucoma	1,860	0.87 (0.74-1.03)	
H25, H26.9 Cataract	17,548	0.88 (0.83-0.93)	Yes
H02 Other disorders of eyelid	540	0.89 (0.68-1.17)	
H47 Other disorders of optic [2nd] nerve and visual pathways	236	1.04 (0.70-1.55)	
H11, H13 Other disorders of conjunctiva	2,710	1.06 (0.96-1.17)	
H04 Disorders of lacrimal system	467	1.08 (0.83-1.41)	
H16 Keratitis	699	1.15 (0.95-1.40)	
H57 Other disorders of eye and adnexa	129	1.36 (0.55-3.39)	
Other	781	1.05 (0.88-1.27)	
**Any**	**26,207**	**0.90 (0.86-0.94)**	**Yes**

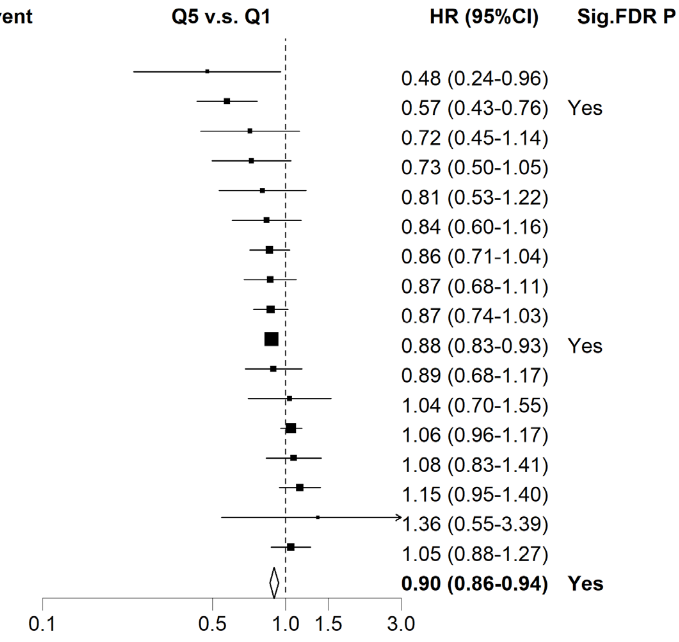

PA: physical activity. HR: hazard ratio.

The x-axis is on a log scale. HRs were stratified by age at risk (5-year groups), sex and ten study areas, and were adjusted for education, drinking status and smoking status.

Figure S10: Adjusted HRs for incidence of specific types of ear and mastoid process diseases associated with physical activity

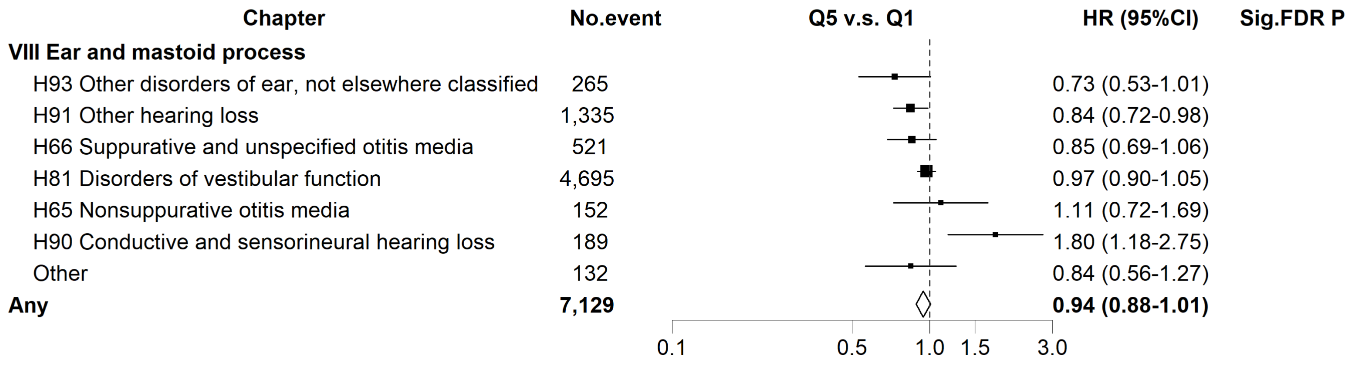

PA: physical activity. HR: hazard ratio.

The x-axis is on a log scale. HRs were stratified by age at risk (5-year groups), sex and ten study areas, and were adjusted for education, drinking status and smoking status.

Figure S11: Adjusted HRs for incidence of specific types of circulatory diseases associated with physical activity

Chapter	No.event	HR (95%CI)	Sig.FDR P
**IX Circulatory**			
I12 Hypertensive renal disease	76	0.47 (0.21-1.03)	
I34-I37 Non-rheumatic heart valve disorders	150	0.47 (0.31-0.73)	
I89 Other noninfective disorders of lymphatic vessels and lymph nodes	135	0.55 (0.35-0.87)	
I27 Other pulmonary heart diseases	2,315	0.56 (0.49-0.64)	Yes
I46 Cardiac arrest	571	0.57 (0.43-0.76)	Yes
I51 Complications and ill-defined descriptions of heart disease	2,150	0.60 (0.52-0.68)	Yes
I05-I09 Chronic rheumatic heart disease	669	0.60 (0.48-0.76)	Yes
I82 Other venous embolism and thrombosis	240	0.62 (0.42-0.91)	
I69 Sequelae of cerebrovascular disease	5,632	0.64 (0.59-0.70)	Yes
I70 Atherosclerosis	1,207	0.66 (0.55-0.80)	Yes
I72 Other aneurysm and dissection	240	0.67 (0.46-0.99)	
I21 Acute myocardial infarction	6,934	0.68 (0.63-0.74)	Yes
I20 Angina pectoris	2,438	0.70 (0.61-0.80)	Yes
I30-I32 Diseases of pericardium	158	0.71 (0.46-1.08)	
I33, I38 Endocarditis	556	0.72 (0.55-0.94)	
I65-I66 Occlusion and stenosis of precerebral or cerebral arteries, not resulting in cerebral infarction	2,915	0.72 (0.63-0.83)	Yes
I25 Chronic ischaemic heart disease	37,971	0.73 (0.70-0.76)	Yes
I50 Heart failure	3,518	0.76 (0.68-0.84)	Yes
I61 Intracerebral haemorrhage	10,401	0.77 (0.73-0.81)	Yes
I63 Cerebral infarction	46,553	0.77 (0.75-0.80)	Yes
I64 Stroke, not specified as haemorrhage or infarction	1,803	0.78 (0.66-0.90)	Yes
I26 Pulmonary embolism	226	0.79 (0.51-1.22)	
I11 Hypertensive heart disease	367	0.82 (0.61-1.11)	
I10 Essential (primary) hypertension	10,830	0.83 (0.78-0.88)	Yes
I44 Atrioventricular and left bundle-branch block	141	0.83 (0.50-1.39)	
I47-I49 Cardiac arrhythmias	5,109	0.84 (0.77-0.90)	Yes
I60 Subarachnoid haemorrhage	978	0.86 (0.71-1.03)	
I80 Phlebitis and thrombophlebitis	696	0.89 (0.72-1.10)	
I42 Cardiomyopathy	701	0.89 (0.74-1.07)	
I24 Other acute ischaemic heart diseases	684	0.90 (0.72-1.13)	
I45 Other conduction disorders	139	0.91 (0.59-1.39)	
I71 Aortic aneurysm and dissection	297	0.94 (0.68-1.32)	
I67 Other cerebrovascular diseases	31,974	0.95 (0.91-0.98)	Yes
I95 Hypotension	126	0.96 (0.63-1.45)	
I62 Other nontraumatic intracranial haemorrhage	529	0.97 (0.78-1.20)	
I74 Arterial embolism and thrombosis	244	0.99 (0.65-1.51)	
I77 Other disorders of arteries and arterioles	173	1.08 (0.74-1.58)	
I88 Nonspecific lymphadenitis	127	1.12 (0.74-1.71)	
I83, I85, I86 Varicose veins	3,386	1.38 (1.29-1.48)	Yes
Other	695	0.65 (0.52-0.81)	Yes
**Any**	**148,757**	**0.77 (0.76-0.79)**	**Yes**

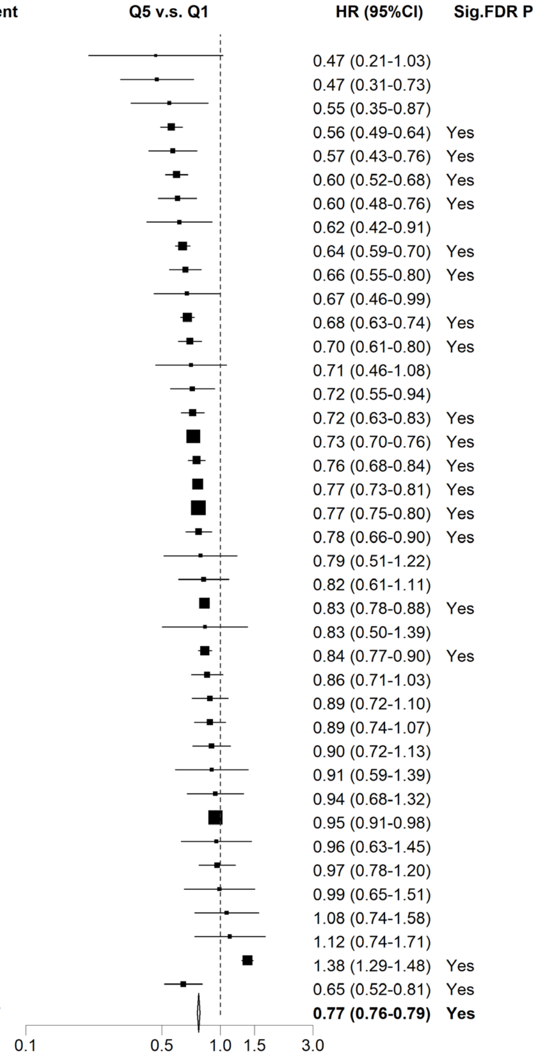

PA: physical activity. HR: hazard ratio.

The x-axis is on a log scale. HRs were stratified by age at risk (5-year groups), sex and ten study areas, and were adjusted for education, drinking status and smoking status.

Figure S12: Adjusted HRs for incidence of specific types of respiratory diseases associated with physical activity

Chapter	No.event	HR (95%CI)	Sig.FDR P
**X Respiratory**			
J96 Respiratory failure, not elsewhere classified	622	0.55 (0.40-0.75)	Yes
J85 Abscess of lung and mediastinum	395	0.58 (0.37-0.90)	
J62 Pneumoconiosis due to dust containing silica	77	0.60 (0.39-0.92)	
J69 Pneumonitis due to solids and liquids	127	0.61 (0.30-1.26)	
J93 Pneumothorax	276	0.69 (0.52-0.92)	
J43 Emphysema	592	0.69 (0.54-0.89)	
J90-J91 Pleural effusion	861	0.77 (0.63-0.94)	
J00 Acute nasopharyngitis [common cold]	548	0.78 (0.65-0.93)	
J44 Other chronic obstructive pulmonary disease	8,995	0.80 (0.75-0.85)	Yes
J98 Other respiratory disorders	1,626	0.80 (0.71-0.91)	
J09-J11 Influenza	280	0.82 (0.59-1.13)	
J45 Asthma	1,826	0.82 (0.71-0.94)	
J47 Bronchiectasis	1,177	0.83 (0.70-0.99)	
J12-J18 Pneumonia	28,270	0.87 (0.84-0.90)	Yes
J30-J39 Other diseases of upper respiratory tract	4,265	0.87 (0.81-0.94)	
J42 Unspecified chronic bronchitis	4,637	0.91 (0.83-0.99)	
J01-J06 Other acute upper respiratory infections (other than common cold)	5,932	0.94 (0.88-1.01)	
J84 Other interstitial pulmonary diseases	611	0.94 (0.73-1.22)	
J40 Bronchitis, not specified as acute or chronic	4,937	1.00 (0.93-1.08)	
J20-J22 Other acute lower respiratory infections	7,832	1.03 (0.97-1.09)	
J41 Simple and mucopurulent chronic bronchitis	73	1.40 (0.59-3.36)	
Other	197	0.80 (0.56-1.15)	
**Any**	**70,273**	**0.84 (0.82-0.86)**	**Yes**

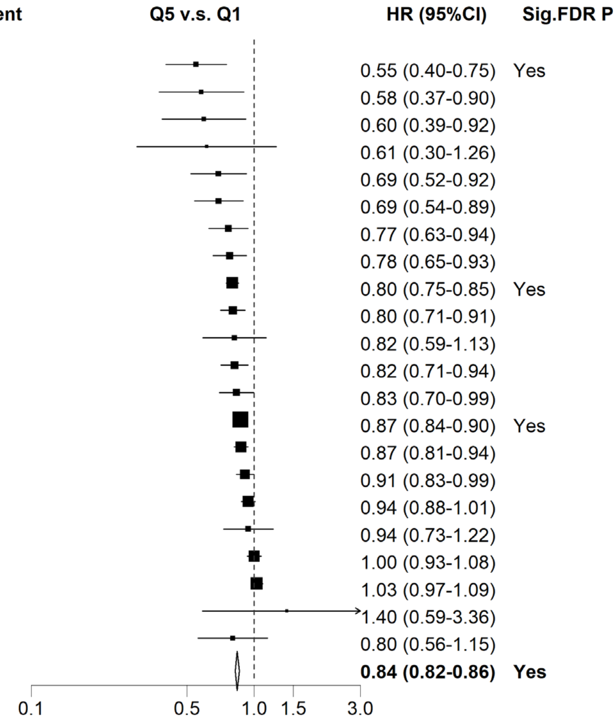

PA: physical activity. HR: hazard ratio.

The x-axis is on a log scale. HRs were stratified by age at risk (5-year groups), sex and ten study areas, and were adjusted for education, drinking status and smoking status.

Figure S13: Adjusted HRs for incidence of specific types of digestive diseases associated with physical activity

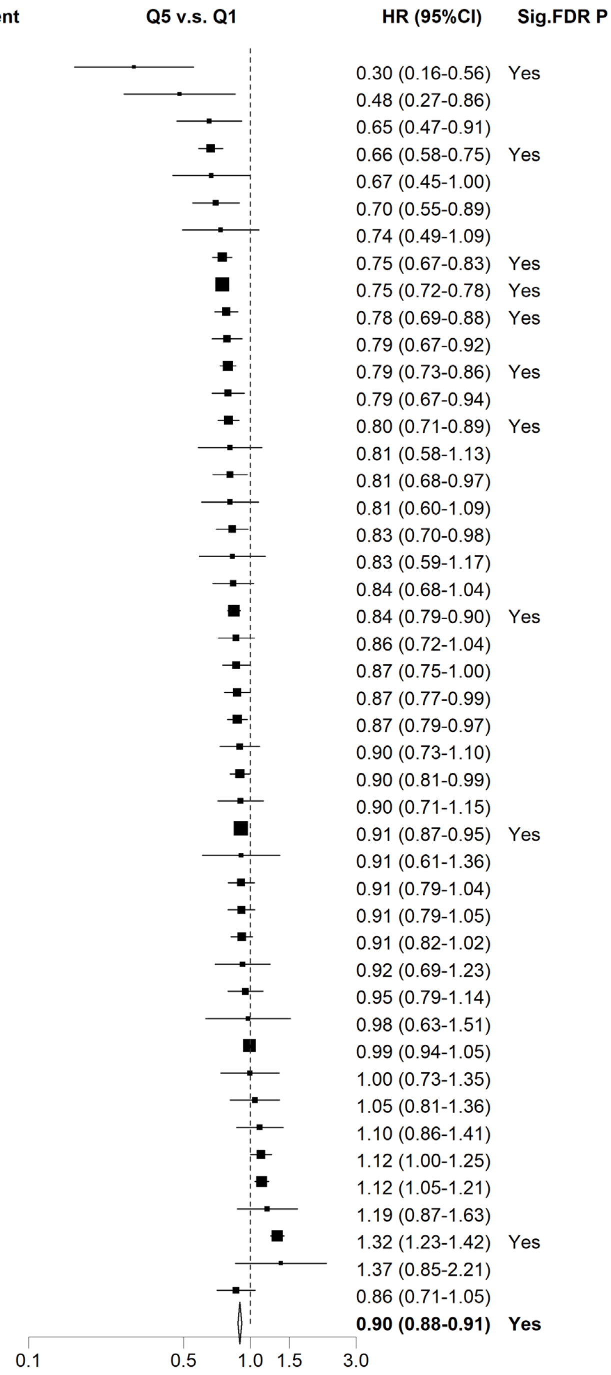

Chapter	No.event	HR (95%CI)	Sig.FDR P
**XI Digestive**			
K77 Liver disorders in diseases classified elsewhere	107	0.30 (0.16-0.56)	Yes
K04 Diseases of pulp and periapical tissues	113	0.48 (0.27-0.86)	
K86 Other diseases of pancreas	221	0.65 (0.47-0.91)	
K76 Other diseases of liver	1,978	0.66 (0.58-0.75)	Yes
K11 Diseases of salivary glands	208	0.67 (0.45-1.00)	
K59 Other functional intestinal disorders	682	0.70 (0.55-0.89)	
K72 Hepatic failure, not elsewhere classified	160	0.74 (0.49-1.09)	
K30 Dyspepsia	2,881	0.75 (0.67-0.83)	Yes
K80 Cholelithiasis	11,545	0.75 (0.72-0.78)	Yes
K74 Fibrosis and cirrhosis of liver	1,898	0.78 (0.69-0.88)	Yes
K83 Other diseases of biliary tract	1,248	0.79 (0.67-0.92)	
K92 Other diseases of digestive system	4,464	0.79 (0.73-0.86)	Yes
K26 Duodenal ulcer	986	0.79 (0.67-0.94)	
K85 Acute pancreatitis	2,120	0.80 (0.71-0.89)	Yes
K51 Ulcerative colitis	334	0.81 (0.58-1.13)	
K82 Other diseases of gallbladder	610	0.81 (0.68-0.97)	
K22 Other diseases of oesophagus	293	0.81 (0.60-1.09)	
K61 Abscess of anal and rectal regions	831	0.83 (0.70-0.98)	
K73 Chronic hepatitis, not elsewhere classified	261	0.83 (0.59-1.17)	
K21 Gastro-oesophageal reflux disease	825	0.84 (0.68-1.04)	
K81 Cholecystitis	6,585	0.84 (0.79-0.90)	Yes
K75 Other inflammatory liver diseases	748	0.86 (0.72-1.04)	
K52 Other noninfective gastroenteritis and colitis	1,548	0.87 (0.75-1.00)	
K62 Other diseases of anus and rectum	1,694	0.87 (0.77-0.99)	
K56 Paralytic ileus and intestinal obstruction without hernia	3,146	0.87 (0.79-0.97)	
K27 Peptic ulcer, site unspecified	806	0.90 (0.73-1.10)	
K63 Other diseases of intestine	2,993	0.90 (0.81-0.99)	
K36 Other appendicitis	406	0.90 (0.71-1.15)	
K29 Gastritis and duodenitis	17,376	0.91 (0.87-0.95)	Yes
K12 Stomatitis and related lesions	162	0.91 (0.61-1.36)	
K60 Fissure and fistula of anal and rectal regions	1,006	0.91 (0.79-1.04)	
K31 Other diseases of stomach and duodenum	1,941	0.91 (0.79-1.05)	
K25 Gastric ulcer	2,202	0.91 (0.82-1.02)	
K66 Other disorders of peritoneum	354	0.92 (0.69-1.23)	
K65 Peritonitis	762	0.95 (0.79-1.14)	
K91 Postprocedural disorders of digestive system, not elsewhere classified	133	0.98 (0.63-1.51)	
K64 Haemorrhoids and perianal venous thrombosis	7,820	0.99 (0.94-1.05)	
K20 Oesophagitis	376	1.00 (0.73-1.35)	
K05 Gingivitis and periodontal diseases	415	1.05 (0.81-1.36)	
K70 Alcoholic liver disease	302	1.10 (0.86-1.41)	
K37 Unspecified appendicitis	1,528	1.12 (1.00-1.25)	
K35 Acute appendicitis	4,110	1.12 (1.05-1.21)	
K71 Toxic liver disease	262	1.19 (0.87-1.63)	
K40 Inguinal hernia	4,189	1.32 (1.23-1.42)	Yes
K38 Other diseases of appendix	130	1.37 (0.85-2.21)	
Other	779	0.86 (0.71-1.05)	
**Any**	**74,232**	**0.90 (0.88-0.91)**	**Yes**

PA: physical activity. HR: hazard ratio.

The x-axis is on a log scale. HRs were stratified by age at risk (5-year groups), sex and ten study areas, and were adjusted for education, drinking status and smoking status.

Figure S14: Adjusted HRs for incidence of specific types of skin and subcutaneous tissue diseases associated with physical activity

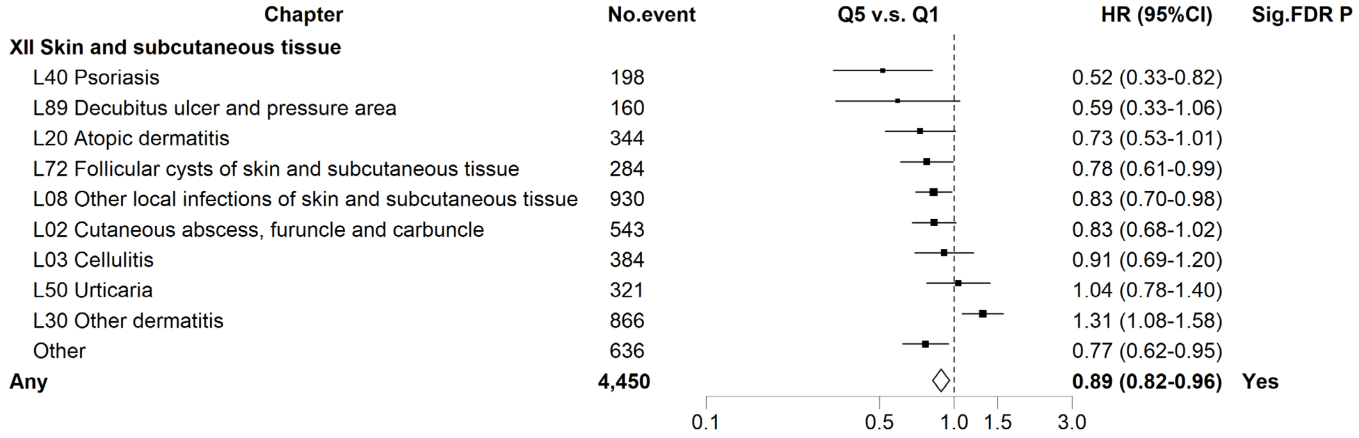

PA: physical activity. HR: hazard ratio.

The x-axis is on a log scale. HRs were stratified by age at risk (5-year groups), sex and ten study areas, and were adjusted for education, drinking status and smoking status.

Figure S15: Adjusted HRs for incidence of specific types of musculoskeletal diseases associated with physical activity

Chapter	No.event	Q5 v.s. Q1	HR (95%CI)	Sig.FDR P
**XIII Musculoskeletal**				
M53 Other dorsopathies, not elsewhere classified	114		0.31 (0.18-0.55)	Yes
M32 Systemic lupus erythematosus	199		0.49 (0.32-0.75)	
M13 Other arthritis	268		0.53 (0.33-0.85)	
M60 Myositis	131		0.59 (0.38-0.93)	
M16 Coxarthrosis [arthrosis of hip]	136		0.69 (0.43-1.10)	
M10 Gout	1,315		0.71 (0.59-0.84)	Yes
M35 Other systemic involvement of connective tissue	449		0.71 (0.55-0.92)	
M81 Osteoporosis without pathological fracture	1,832		0.72 (0.60-0.86)	Yes
M45 Ankylosing spondylitis	130		0.73 (0.50-1.07)	
M75 Shoulder lesions	1,009		0.85 (0.73-0.99)	
M87 Osteonecrosis	634		0.85 (0.69-1.04)	
M85 Other disorders of bone density and structure	250		0.86 (0.60-1.23)	
M50 Cervical disc disorders	3,062		0.87 (0.79-0.97)	
M79 Other soft tissue disorders, not elsewhere classified	819		0.88 (0.72-1.07)	
M47 Spondylosis	17,121		0.90 (0.87-0.94)	Yes
M06 Other rheumatoid arthritis	1,955		0.93 (0.83-1.05)	
M65 Synovitis and tenosynovitis	561		0.95 (0.77-1.16)	
M48 Other spondylopathies	2,931		0.95 (0.85-1.07)	
M25 Other joint disorders, not elsewhere classified	884		0.96 (0.81-1.14)	
M54 Dorsalgia	1,756		0.97 (0.86-1.09)	
M51 Other intervertebral disc disorders	18,888		0.98 (0.95-1.02)	
M17 Gonarthrosis [arthrosis of knee]	2,992		1.00 (0.89-1.11)	
M80 Osteoporosis with pathological fracture	633		1.01 (0.78-1.29)	
M43 Other deforming dorsopathies	401		1.04 (0.83-1.32)	
M67 Other disorders of synovium and tendon	370		1.05 (0.85-1.29)	
M62 Other disorders of muscle	326		1.05 (0.81-1.36)	
M84 Disorders of continuity of bone	182		1.06 (0.72-1.55)	
M19 Other arthrosis	7,870		1.08 (1.01-1.16)	
M86 Osteomyelitis	154		1.13 (0.76-1.68)	
M77 Other enthesopathies	121		1.25 (0.87-1.79)	
M72 Fibroblastic disorders	167		1.32 (0.91-1.92)	
M23 Internal derangement of knee	336		1.59 (1.24-2.04)	
M71 Other bursopathies	356		1.94 (1.59-2.37)	Yes
Other	993		0.72 (0.61-0.86)	Yes
**Any**	**55,180**		**0.95 (0.92-0.97)**	**Yes**

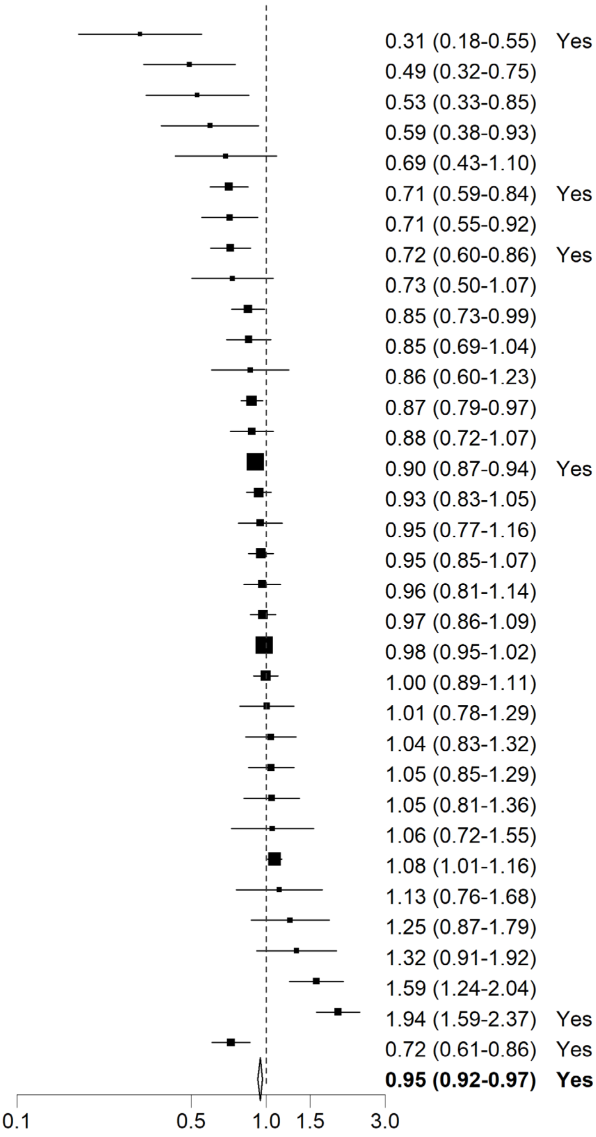

PA: physical activity. HR: hazard ratio.

The x-axis is on a log scale. HRs were stratified by age at risk (5-year groups), sex and ten study areas, and were adjusted for education, drinking status and smoking status.

Figure S16: Adjusted HRs for incidence of specific types of genitourinary diseases associated with physical activity

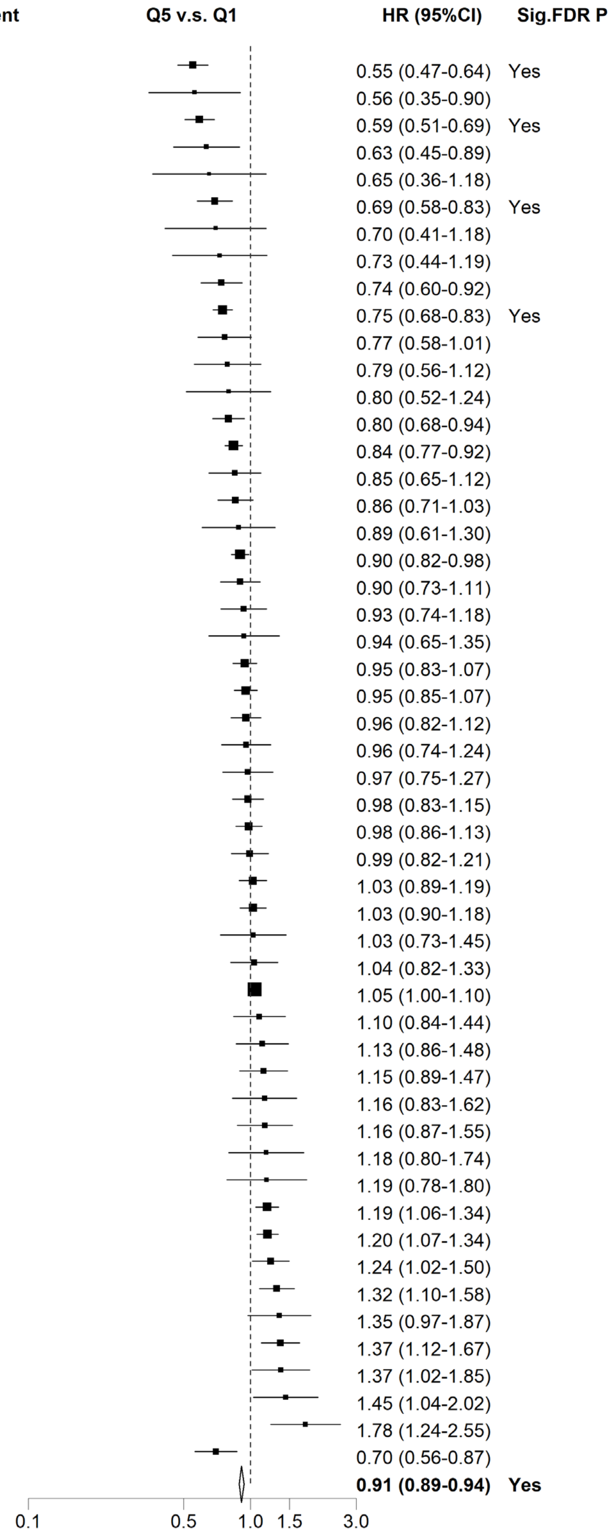

Chapter	No.event	HR (95%CI)	Sig.FDR P
**XIV Genitourinary**			
N18 Chronic kidney disease	1,867	0.55 (0.47-0.64)	Yes
N17 Acute renal failure	156	0.56 (0.35-0.90)	
N19 Unspecified kidney failure	1,769	0.59 (0.51-0.69)	Yes
N32 Other disorders of bladder	294	0.63 (0.45-0.89)	
N11 Chronic tubulo-interstitial nephritis	129	0.65 (0.36-1.18)	
N03 Chronic nephritic syndrome	921	0.69 (0.58-0.83)	Yes
N64 Other disorders of breast	130	0.70 (0.41-1.18)	
N00 Acute nephritic syndrome	140	0.73 (0.44-1.19)	
N10 Acute tubulo-interstitial nephritis	899	0.74 (0.60-0.92)	
N40 Hyperplasia of prostate	3,790	0.75 (0.68-0.83)	Yes
N04 Nephrotic syndrome	478	0.77 (0.58-1.01)	
N92 Excessive, frequent and irregular menstruation	195	0.79 (0.56-1.12)	
N35 Urethral stricture	138	0.80 (0.52-1.24)	
N41 Inflammatory diseases of prostate	781	0.80 (0.68-0.94)	
N73 Other female pelvic inflammatory diseases	2,524	0.84 (0.77-0.92)	
N85 Other noninflammatory disorders of uterus, except cervix	327	0.85 (0.65-1.12)	
N45 Orchitis and epididymitis	539	0.86 (0.71-1.03)	
N95 Menopausal and other perimenopausal disorders	204	0.89 (0.61-1.30)	
N39 Other disorders of urinary system	4,183	0.90 (0.82-0.98)	
N30 Cystitis	693	0.90 (0.73-1.11)	
N34 Urethritis and urethral syndrome	456	0.93 (0.74-1.18)	
N61 Inflammatory disorders of breast	170	0.94 (0.65-1.35)	
N80 Endometriosis	1,119	0.95 (0.83-1.07)	
N84 Polyp of female genital tract	1,524	0.95 (0.85-1.07)	
N83 Noninflammatory disorders of ovary, fallopian tube and broad ligament	941	0.96 (0.82-1.12)	
N05 Unspecified nephritic syndrome	351	0.96 (0.74-1.24)	
N12 Tubulo-interstitial nephritis, not specified as acute or chronic	371	0.97 (0.75-1.27)	
N28 Other disorders of kidney and ureter, not elsewhere classified	1,068	0.98 (0.83-1.15)	
N23 Unspecified renal colic	1,631	0.98 (0.86-1.13)	
N70 Salpingitis and oophoritis	405	0.99 (0.82-1.21)	
N13 Obstructive and reflux uropathy	1,150	1.03 (0.89-1.19)	
N72 Inflammatory disease of cervix uteri	1,006	1.03 (0.90-1.18)	
N36 Other disorders of urethra	233	1.03 (0.73-1.45)	
N75 Diseases of Bartholin gland	291	1.04 (0.82-1.33)	
N20 Calculus of kidney and ureter	10,057	1.05 (1.00-1.10)	
N71 Inflammatory disease of uterus, except cervix	362	1.10 (0.84-1.44)	
N43 Hydrocele and spermatocele	251	1.13 (0.86-1.48)	
N60 Benign mammary dysplasia	394	1.15 (0.89-1.47)	
N88 Other noninflammatory disorders of cervix uteri	165	1.16 (0.83-1.62)	
N87 Dysplasia of cervix uteri	238	1.16 (0.87-1.55)	
N47 Redundant prepuce, phimosis and paraphimosis	111	1.18 (0.80-1.74)	
N90 Other noninflammatory disorders of vulva and perineum	129	1.19 (0.78-1.80)	
N63 Unspecified lump in breast	1,217	1.19 (1.06-1.34)	
N93 Other abnormal uterine and vaginal bleeding	1,512	1.20 (1.07-1.34)	
N76 Other inflammation of vagina and vulva	527	1.24 (1.02-1.50)	
N81 Female genital prolapse	947	1.32 (1.10-1.58)	
N50 Other disorders of male genital organs	158	1.35 (0.97-1.87)	
N21 Calculus of lower urinary tract	643	1.37 (1.12-1.67)	
N86 Erosion and ectropion of cervix uteri	148	1.37 (1.02-1.85)	
N62 Hypertrophy of breast	194	1.45 (1.04-2.02)	
N15 Other renal tubulo-interstitial diseases	142	1.78 (1.24-2.55)	
Other	487	0.70 (0.56-0.87)	
**Any**	**40,884**	**0.91 (0.89-0.94)**	**Yes**

PA: physical activity. HR: hazard ratio.

The x-axis is on a log scale. HRs were stratified by age at risk (5-year groups), sex and ten study areas, and were adjusted for education, drinking status and smoking status.

Figure S17: Adjusted HRs for incidence of specific types of pregnancy-related diseases associated with physical activity

Chapter	No.event	Q5 v.s. Q1	HR (95%CI)	Sig.FDR P
**XV Pregnancy-related**				
O82 Single delivery by caesarean section	345		0.95 (0.72-1.25)	
O00 Ectopic pregnancy	229		1.07 (0.78-1.48)	
O80 Single spontaneous delivery	1,004		1.14 (0.96-1.36)	
Other	252		1.17 (0.84-1.63)	
**Any**	**1,783**		**1.14 (1.00-1.29)**	

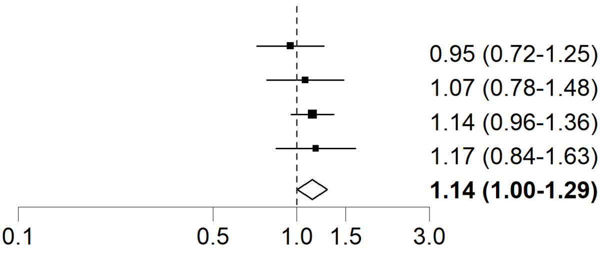

PA: physical activity. HR: hazard ratio.

The x-axis is on a log scale. HRs were stratified by age at risk (5-year groups), sex and ten study areas, and were adjusted for education, drinking status and smoking status.

Figure S18: Adjusted HRs for incidence of other symptoms, signs and abnormal findings associated with physical activity

Chapter	No.event	HR (95%CI)	Sig.FDR P
**XVIII Other symptoms, signs and abnormal findings**			
R73 Elevated blood glucose level	147	0.24 (0.13-0.46)	Yes
R40 Somnolence, stupor and coma	109	0.49 (0.26-0.92)	
R06 Abnormalities of breathing	223	0.52 (0.33-0.83)	
R96 Other sudden death, cause unknown	221	0.61 (0.36-1.02)	
R65 Systemic Inflammatory Response Syndrome [SIRS]	147	0.62 (0.33-1.18)	
R99 Other ill-defined and unspecified causes of mortality	700	0.63 (0.47-0.86)	
R53 Malaise and fatigue	513	0.69 (0.56-0.85)	
R33 Retention of urine	743	0.72 (0.57-0.90)	
R20 Disturbances of skin sensation	165	0.75 (0.46-1.24)	
R18 Ascites	293	0.76 (0.55-1.04)	
R90 Abnormal findings on diagnostic imaging of central nervous system	372	0.76 (0.57-1.02)	
R69 Unknown and unspecified causes of morbidity	21,300	0.78 (0.76-0.81)	Yes
R50 Fever of other and unknown origin	1,554	0.79 (0.69-0.91)	
R09 Other symptoms and signs involving the circulatory and respiratory systems	459	0.80 (0.61-1.04)	
R93 Abnormal findings on diagnostic imaging of other body structures	762	0.80 (0.65-0.98)	
R94 Abnormal results of function studies	831	0.82 (0.70-0.96)	
R10 Abdominal and pelvic pain	3,821	0.82 (0.75-0.90)	Yes
R31 Unspecified haematuria	370	0.85 (0.64-1.14)	
R60 Oedema, not elsewhere classified	416	0.86 (0.63-1.16)	
R51 Headache	2,851	0.88 (0.81-0.95)	
R91 Abnormal findings on diagnostic imaging of lung	687	0.88 (0.72-1.07)	
R55 Syncope and collapse	499	0.89 (0.69-1.14)	
R04 Haemorrhage from respiratory passages	1,031	0.89 (0.76-1.05)	
R05 Cough	526	0.90 (0.68-1.18)	
R59 Enlarged lymph nodes	123	0.90 (0.61-1.33)	
R19 Other symptoms and signs involving the digestive system and abdomen	606	0.90 (0.73-1.12)	
R22 Localized swelling, mass and lump of skin and subcutaneous tissue	1,244	0.91 (0.80-1.03)	
R07 Pain in throat and chest	1,006	0.92 (0.77-1.10)	
R42 Dizziness and giddiness	5,075	0.93 (0.86-1.01)	
R57 Shock, not elsewhere classified	346	0.99 (0.73-1.33)	
R11 Nausea and vomiting	205	1.00 (0.64-1.58)	
R00 Abnormalities of heart beat	1,095	1.13 (0.97-1.31)	
R17 Unspecified jaundice	181	1.24 (0.85-1.80)	
Other	751	0.69 (0.56-0.84)	Yes
**Any**	**43,764**	**0.83 (0.81-0.85)**	**Yes**

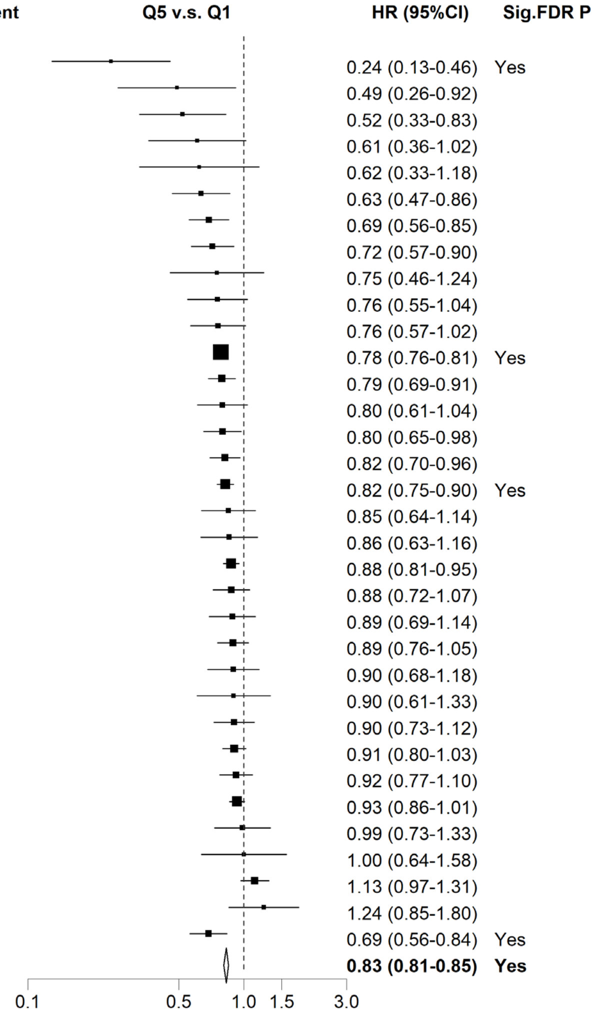

PA: physical activity. HR: hazard ratio.

The x-axis is on a log scale. HRs were stratified by age at risk (5-year groups), sex and ten study areas, and were adjusted for education, drinking status and smoking status.

Figure S19: Adjusted HRs for incidence of specific types of injury, poisoning and other external causes associated with physical activity

Chapter	No.event	HR (95%CI)	Sig.FDR P
**XIX Injury, poisoning and other external causes**			
S02 Fracture of skull and facial bones	304	0.60 (0.46-0.78)	
T00 Superficial injuries involving multiple body regions	315	0.79 (0.58-1.08)	
S39 Other and unspecified injuries of abdomen, lower back and pelvis	172	0.83 (0.56-1.22)	
S72 Fracture of femur	3,561	0.85 (0.76-0.94)	
T84 Complications of internal orthopaedic prosthetic devices, implants and grafts	172	0.86 (0.59-1.25)	
S09 Other and unspecified injuries of head	747	0.89 (0.77-1.04)	
S01 Open wound of head	269	0.91 (0.68-1.21)	
T18 Foreign body in alimentary tract	110	0.94 (0.50-1.78)	
S13 Dislocation, sprain and strain of joints and ligaments at neck level	164	0.97 (0.59-1.59)	
S52 Fracture of forearm	2,204	0.97 (0.86-1.08)	
S32 Fracture of lumbar spine and pelvis	2,700	0.98 (0.88-1.08)	
S42 Fracture of shoulder and upper arm	2,164	1.01 (0.91-1.12)	
S82 Fracture of lower leg, including ankle	3,212	1.04 (0.96-1.12)	
T02 Fractures involving multiple body regions	142	1.08 (0.81-1.43)	
S43 Dislocation, sprain and strain of joints and ligaments of shoulder girdle	233	1.08 (0.80-1.47)	
S06 Intracranial injury	1,619	1.09 (0.96-1.22)	
S83 Dislocation, sprain and strain of joints and ligaments of knee	204	1.12 (0.82-1.53)	
S22 Fracture of rib(s), sternum and thoracic spine	2,477	1.13 (1.03-1.24)	
T14 Injury of unspecified body region	5,419	1.16 (1.09-1.23)	Yes
S05 Injury of eye and orbit	333	1.16 (0.90-1.49)	
S80 Superficial injury of lower leg	110	1.16 (0.75-1.81)	
S62 Fracture at wrist and hand level	414	1.19 (0.98-1.46)	
S92 Fracture of foot, except ankle	697	1.23 (1.06-1.44)	
S69 Other and unspecified injuries of wrist and hand	274	1.29 (1.04-1.60)	
T30 Burn and corrosion, body region unspecified	381	1.31 (1.05-1.63)	
S00 Superficial injury of head	464	1.36 (1.08-1.70)	
T63 Toxic effect of contact with venomous animals	285	1.38 (1.01-1.89)	
S60 Superficial injury of wrist and hand	162	1.42 (1.05-1.91)	
S29 Other and unspecified injuries of thorax	101	1.55 (1.03-2.32)	
S33 Dislocation, sprain and strain of joints and ligaments of lumbar spine and pelvis	164	1.77 (1.21-2.58)	
S27 Injury of other and unspecified intrathoracic organs	131	1.97 (1.40-2.77)	
Other	2,311	1.19 (1.08-1.31)	
**Any**	**29,119**	**1.06 (1.03-1.09)**	**Yes**

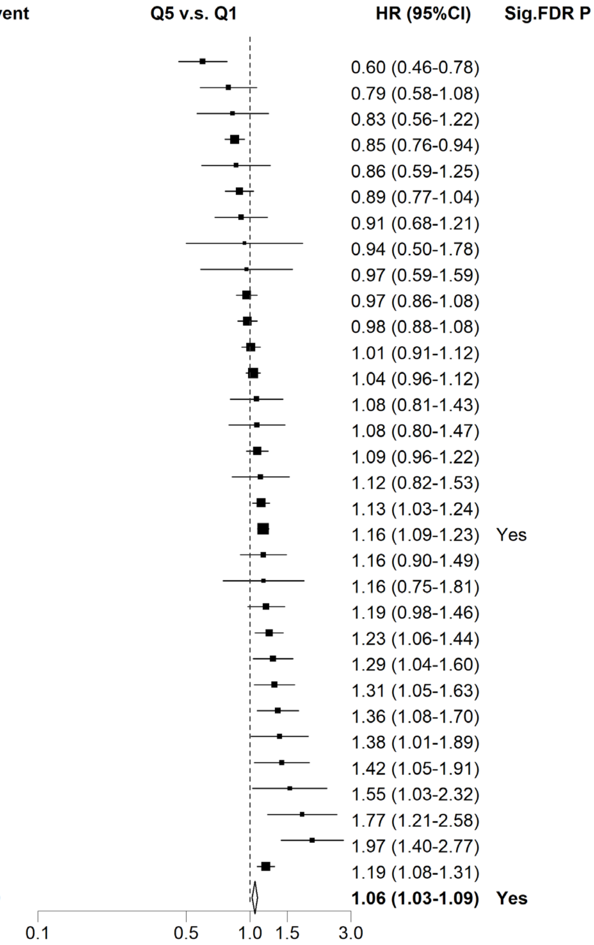

PA: physical activity. HR: hazard ratio.

The x-axis is on a log scale. HRs were stratified by age at risk (5-year groups), sex and ten study areas, and were adjusted for education, drinking status and smoking status.

Figure S20: Adjusted HRs for incidence of specific external causes associated with physical activity

Chapter	No.event	Q5 v.s. Q1	HR (95%CI)	Sig.FDR P
**XX External causes**				
W01 Fall on same level from slipping, tripping and stumbling	245		0.59 (0.36-0.95)	
Y17 Poisoning by and exposure to other gases and vapours, undetermined intent	178		0.77 (0.52-1.16)	
W70 Drowning and submersion following fall into natural water	103		0.79 (0.49-1.26)	
X70 Intentional self-harm by hanging, strangulation and suffocation	227		0.79 (0.56-1.12)	
X68 Intentional self-poisoning by and exposure to pesticides	227		0.80 (0.58-1.10)	
V03 Pedestrian injured in collision with car, pick-up truck or van	365		1.11 (0.86-1.44)	
V23 Motorcycle rider injured in collision with car, pick-up truck or van	117		1.27 (0.90-1.79)	
V13 Pedal cyclist injured in collision with car, pick-up truck or van	116		1.29 (0.92-1.80)	
W13 Fall from, out of or through building or structure	136		1.32 (0.92-1.89)	
Other	2,275		0.99 (0.90-1.10)	
**Any**	**3,973**		**0.97 (0.90-1.05)**	

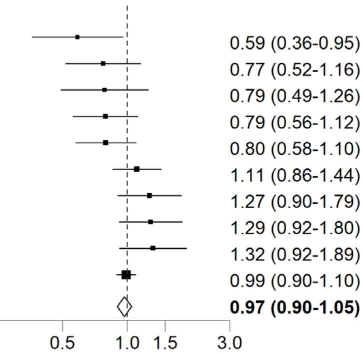

PA: physical activity. HR: hazard ratio.

The x-axis is on a log scale. HRs were stratified by age at risk (5-year groups), sex and ten study areas, and were adjusted for education, drinking status and smoking status.

Figure S21: Wide landscapes of diseases associated with the highest quintile group of domain-specific physical activity increment after FDR adjustment by ICD-10 chapters

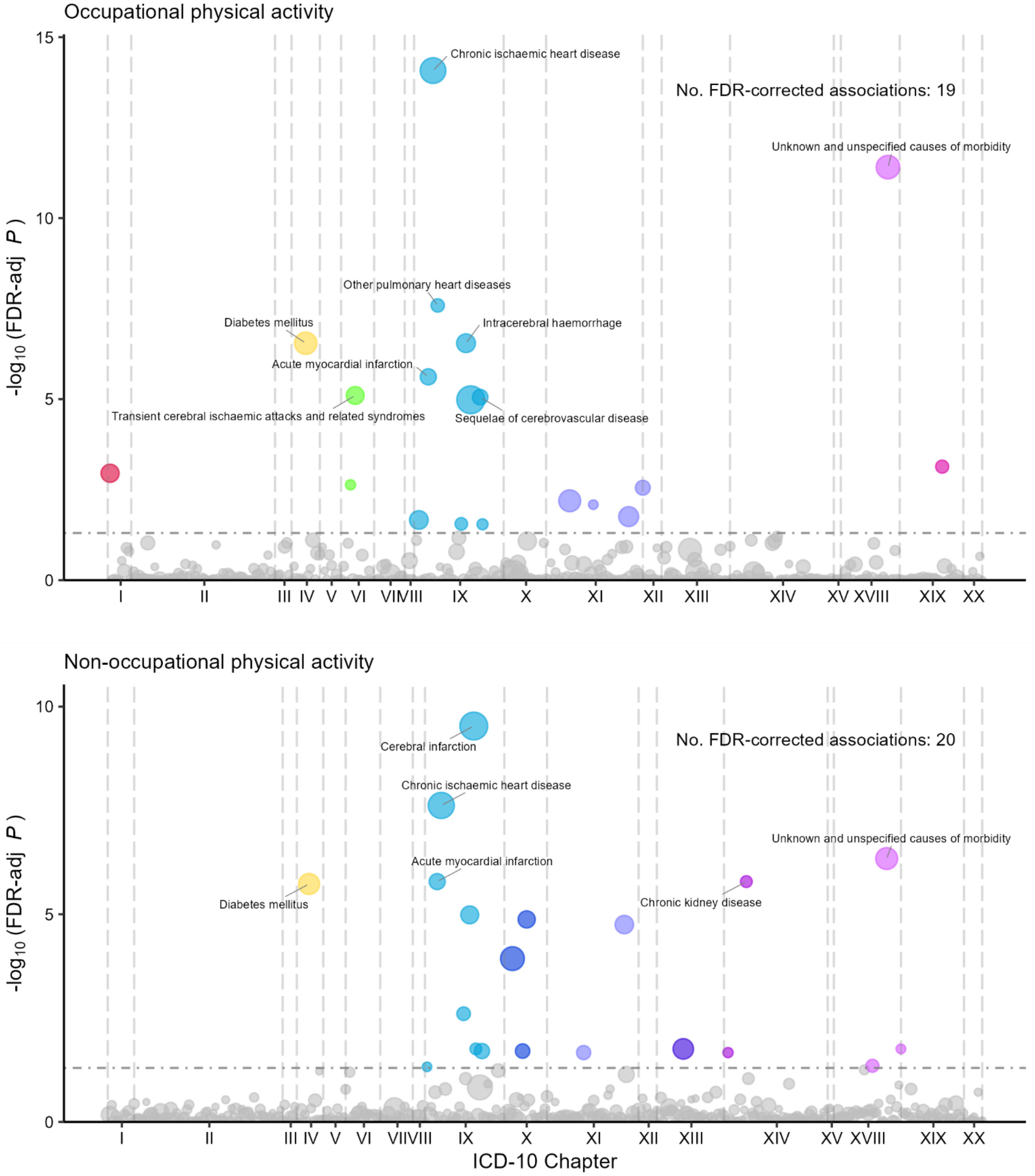

The y-axis represents the negative log (10) of the phenome-wide *P* value after FDR adjustment. Horizontal gray dashed line indicates the cut-off for 0.05. The size of the point is proportional to the number of cases. The models were stratified by age at risk (5-year groups), sex and ten study areas, and were adjusted for education, drinking status, smoking status and occupational physical activity or non-occupational physical activity.

Figure S22: Adjusted HRs for specific diseases showing significant associations with domain-specific physical activity after FDR adjustment by ICD-10 chapters

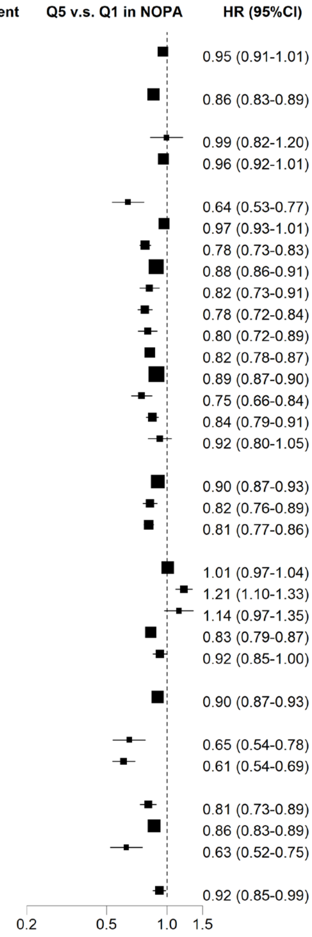

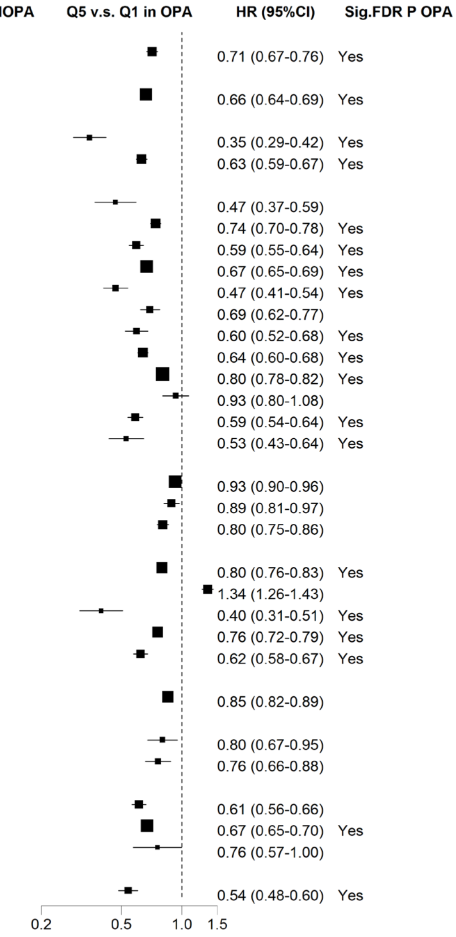

Disease	No.event	HR (95%CI)	Sig.FDR P NOPA	HR (95%CI)	Sig.FDR P OPA
**I Infectious and parasitic**					
A00-A09 Intestinal infectious diseases	8,449	0.95 (0.91-1.01)		0.71 (0.67-0.76)	Yes
**IV Endocrine, nutritional and metabolic**					
E10-E14 Diabetes mellitus	18,448	0.86 (0.83-0.89)	Yes	0.66 (0.64-0.69)	Yes
**VI Nerve-related**					
G40 Epilepsy	818	0.99 (0.82-1.20)		0.35 (0.29-0.42)	Yes
G45 Transient cerebral ischaemic attacks and related syndromes	10,579	0.96 (0.92-1.01)		0.63 (0.59-0.67)	Yes
**IX Circulatory**					
I05-I09 Chronic rheumatic heart disease	669	0.64 (0.53-0.77)	Yes	0.47 (0.37-0.59)	
I10 Essential (primary) hypertension	10,830	0.97 (0.93-1.01)		0.74 (0.70-0.78)	Yes
I21 Acute myocardial infarction	6,934	0.78 (0.73-0.83)	Yes	0.59 (0.55-0.64)	Yes
I25 Chronic ischaemic heart disease	37,971	0.88 (0.86-0.91)	Yes	0.67 (0.65-0.69)	Yes
I27 Other pulmonary heart diseases	2,315	0.82 (0.73-0.91)		0.47 (0.41-0.54)	Yes
I50 Heart failure	3,518	0.78 (0.72-0.84)	Yes	0.69 (0.62-0.77)	
I51 Complications and ill-defined descriptions of heart disease	2,150	0.80 (0.72-0.89)		0.60 (0.52-0.68)	Yes
I61 Intracerebral haemorrhage	10,401	0.82 (0.78-0.87)	Yes	0.64 (0.60-0.68)	Yes
I63 Cerebral infarction	46,553	0.89 (0.87-0.90)	Yes	0.80 (0.78-0.82)	Yes
I64 Stroke, not specified as haemorrhage or infarction	1,803	0.75 (0.66-0.84)	Yes	0.93 (0.80-1.08)	
I69 Sequelae of cerebrovascular disease	5,632	0.84 (0.79-0.91)	Yes	0.59 (0.54-0.64)	Yes
I70 Atherosclerosis	1,207	0.92 (0.80-1.05)		0.53 (0.43-0.64)	Yes
**X Respiratory**					
J12-J18 Pneumonia	28,270	0.90 (0.87-0.93)	Yes	0.93 (0.90-0.96)	
J42 Unspecified chronic bronchitis	4,637	0.82 (0.76-0.89)	Yes	0.89 (0.81-0.97)	
J44 Other chronic obstructive pulmonary disease	8,995	0.81 (0.77-0.86)	Yes	0.80 (0.75-0.86)	
**XI Digestive**					
K29 Gastritis and duodenitis	17,376	1.01 (0.97-1.04)		0.80 (0.76-0.83)	Yes
K40 Inguinal hernia	4,189	1.21 (1.10-1.33)	Yes	1.34 (1.26-1.43)	
K59 Other functional intestinal disorders	682	1.14 (0.97-1.35)		0.40 (0.31-0.51)	Yes
K80 Cholelithiasis	11,545	0.83 (0.79-0.87)	Yes	0.76 (0.72-0.79)	Yes
K92 Other diseases of digestive system	4,464	0.92 (0.85-1.00)		0.62 (0.58-0.67)	Yes
**XIII Musculoskeletal**					
M47 Spondylosis	17,121	0.90 (0.87-0.93)	Yes	0.85 (0.82-0.89)	
**XIV Genitourinary**					
N03 Chronic nephritic syndrome	921	0.65 (0.54-0.78)	Yes	0.80 (0.67-0.95)	
N18 Chronic kidney disease	1,867	0.61 (0.54-0.69)	Yes	0.76 (0.66-0.88)	
**XVIII Other symptoms, signs and abnormal findings**					
R51 Headache	2,851	0.81 (0.73-0.89)	Yes	0.61 (0.56-0.66)	
R69 Unknown and unspecified causes of morbidity	21,300	0.86 (0.83-0.89)	Yes	0.67 (0.65-0.70)	Yes
R99 Other ill-defined and unspecified causes of mortality	700	0.63 (0.52-0.75)	Yes	0.76 (0.57-1.00)	
**XIX Injury, poisoning and other external causes**					
S72 Fracture of femur	3,561	0.92 (0.85-0.99)		0.54 (0.48-0.60)	Yes

HR: hazard ratio; NOPA: non-occupational physical activity.

The x-axis is on a log scale. HRs were stratified by age at risk (5-year groups), sex and ten study areas, and were adjusted for education, drinking status, smoking status and occupational physical activity or non-occupational physical activity.

Figure S23: Wide landscapes of diseases associated with the highest quintile group of intensity-specific physical activity increment after FDR adjustment by ICD-10 chapters

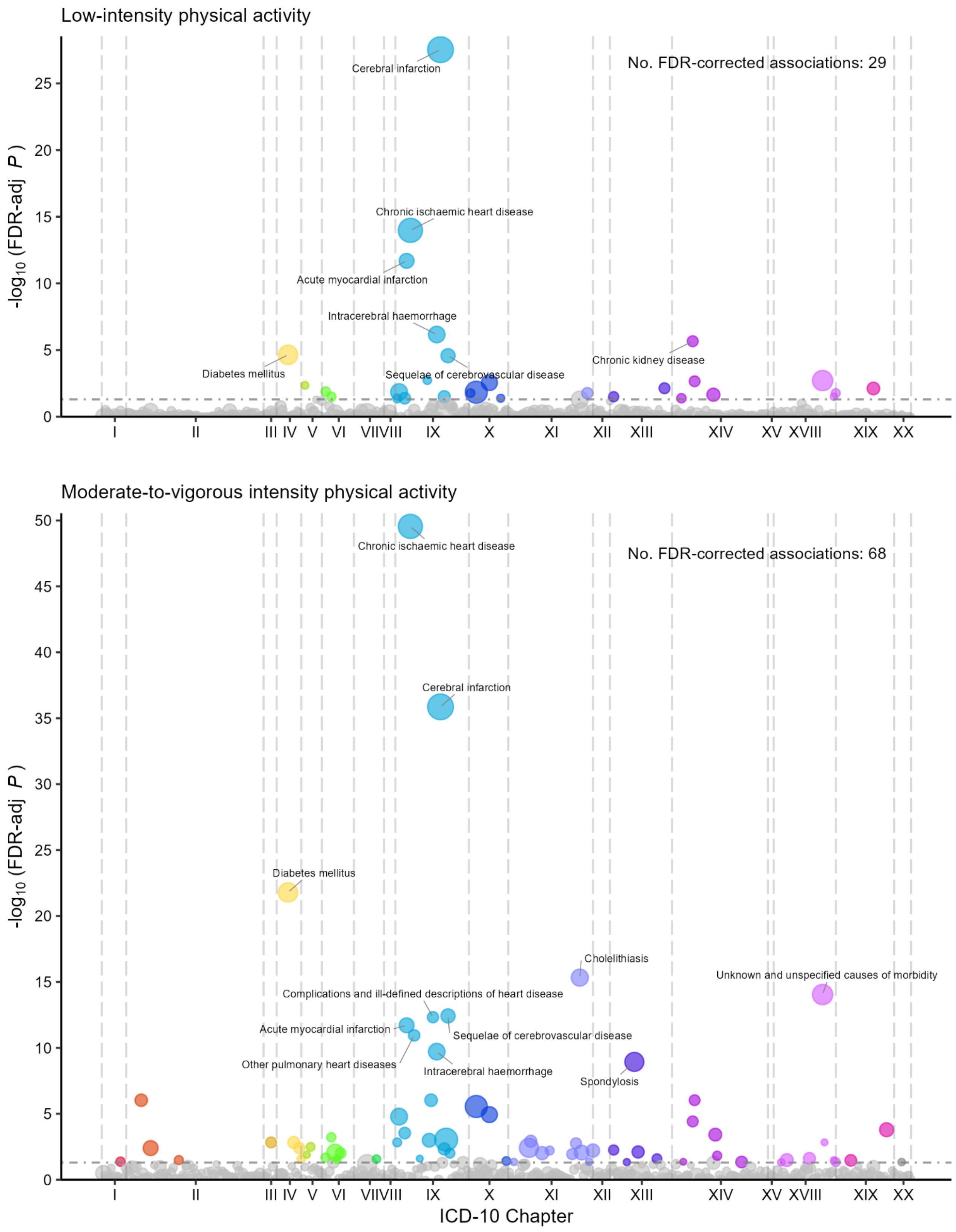

The y-axis represents the negative log (10) of the phenome-wide *P* value after FDR adjustment. Horizontal gray dashed line indicates the cut-off for 0.05. The size of the point is proportional to the number of cases. The models were stratified by age at risk (5-year groups), sex and ten study areas, and were adjusted for education, drinking status, smoking status and low-intensity physical activity or moderate-to-vigorous intensity physical activity.

Figure S24: Adjusted HRs for specific diseases showing significant associations with intensity-specific physical activity after FDR adjustment by ICD-10 chapters

Disease	No.event	Q5 v.s. Q1 in MVPA	HR (95%CI)	Sig.FDR P MVPA	Q5 v.s. Q1 in LIPA	HR (95%CI)	Sig.FDR P LIPA
**I Infectious and parasitic**							
B18 Chronic viral hepatitis	888		0.73 (0.62-0.87)	Yes		0.79 (0.67-0.93)	
**II Neoplasms**							
C22 Malignant neoplasm of liver and intrahepatic bile ducts	3,364		0.71 (0.64-0.78)	Yes		0.97 (0.88-1.06)	
C34 Malignant neoplasm of bronchus and lung	7,041		0.85 (0.80-0.92)	Yes		0.92 (0.86-0.98)	
C73 Malignant neoplasm of thyroid gland	734		0.70 (0.56-0.86)	Yes		0.82 (0.70-0.95)	
**III Blood and immune-related**							
D64 Other anaemias	1,778		0.71 (0.61-0.81)	Yes		0.83 (0.74-0.93)	
**IV Endocrine, nutritional and metabolic**							
E10-E14 Diabetes mellitus	18,448		0.77 (0.74-0.80)	Yes		0.85 (0.83-0.88)	Yes
E78 Disorders of lipoprotein metabolism and other lipidaemias	2,839		0.75 (0.67-0.83)	Yes		0.98 (0.90-1.07)	
E87 Other disorders of fluid, electrolyte and acid-base balance	2,082		0.74 (0.66-0.83)	Yes		0.88 (0.80-0.97)	
E88 Other metabolic disorders	461		0.57 (0.42-0.75)	Yes		1.01 (0.81-1.26)	
**V Mental and behavioural**							
F01-F03 Dementia	309		0.59 (0.40-0.88)			0.38 (0.26-0.56)	Yes
F06 Other mental disorders due to brain damage and dysfunction and to physical disease	168		0.38 (0.23-0.63)	Yes		0.63 (0.43-0.91)	
F20 Schizophrenia	614		0.60 (0.49-0.74)	Yes		0.68 (0.56-0.83)	
**VI Nerve-related**							
G20 Parkinson disease	899		0.66 (0.52-0.83)	Yes		0.62 (0.52-0.75)	Yes
G40 Epilepsy	818		0.60 (0.49-0.72)	Yes		0.65 (0.54-0.79)	Yes
G45 Transient cerebral ischaemic attacks and related syndromes	10,579		0.88 (0.82-0.94)	Yes		0.94 (0.90-0.99)	
G47 Sleep disorders	445		0.60 (0.46-0.78)	Yes		0.76 (0.61-0.94)	
G51 Facial nerve disorders	1,554		0.74 (0.64-0.85)	Yes		0.99 (0.87-1.11)	
**VII Eye and adnexa**							
H43 Disorders of vitreous body	492		0.61 (0.46-0.80)	Yes		0.71 (0.57-0.89)	
**IX Circulatory**							
I05-I09 Chronic rheumatic heart disease	669		0.57 (0.45-0.72)	Yes		0.60 (0.50-0.72)	Yes
I10 Essential (primary) hypertension	10,830		0.83 (0.79-0.88)	Yes		0.86 (0.82-0.90)	Yes
I20 Angina pectoris	2,438		0.70 (0.61-0.81)	Yes		0.76 (0.69-0.85)	Yes
I21 Acute myocardial infarction	6,934		0.70 (0.65-0.76)	Yes		0.68 (0.64-0.73)	Yes
I25 Chronic ischaemic heart disease	37,971		0.73 (0.70-0.76)	Yes		0.83 (0.81-0.86)	Yes
I27 Other pulmonary heart diseases	2,315		0.54 (0.48-0.62)	Yes		0.83 (0.75-0.92)	
I34-I37 Non-rheumatic heart valve disorders	150		0.40 (0.25-0.66)	Yes		0.77 (0.52-1.13)	
I46 Cardiac arrest	571		0.69 (0.52-0.90)			0.49 (0.37-0.63)	Yes
I47-I49 Cardiac arrhythmias	5,109		0.81 (0.75-0.88)	Yes		1.03 (0.96-1.10)	
I50 Heart failure	3,518		0.70 (0.63-0.77)	Yes		0.86 (0.79-0.94)	
I51 Complications and ill-defined descriptions of heart disease	2,150		0.53 (0.46-0.61)	Yes		0.80 (0.72-0.89)	
I61 Intracerebral haemorrhage	10,401		0.78 (0.74-0.83)	Yes		0.79 (0.75-0.83)	Yes
I63 Cerebral infarction	46,553		0.79 (0.77-0.81)	Yes		0.80 (0.78-0.82)	Yes
I65-I66 Occlusion and stenosis of precerebral or cerebral arteries, not resulting in cerebral infarction	2,915		0.76 (0.67-0.87)	Yes		0.78 (0.71-0.86)	Yes
I67 Other cerebrovascular diseases	31,974		0.92 (0.89-0.95)	Yes		0.95 (0.92-0.97)	
I69 Sequelae of cerebrovascular disease	5,632		0.67 (0.61-0.72)	Yes		0.76 (0.71-0.82)	Yes
I70 Atherosclerosis	1,207		0.68 (0.56-0.82)	Yes		0.74 (0.64-0.86)	
**X Respiratory**							
J00 Acute nasopharyngitis [common cold]	548		0.84 (0.69-1.02)			1.87 (1.57-2.22)	Yes
J12-J18 Pneumonia	28,270		0.89 (0.86-0.92)	Yes		0.92 (0.89-0.94)	Yes
J44 Other chronic obstructive pulmonary disease	8,995		0.81 (0.77-0.87)	Yes		0.84 (0.79-0.88)	Yes
J85 Abscess of lung and mediastinum	395		0.70 (0.47-1.05)			0.51 (0.36-0.73)	Yes
J96 Respiratory failure, not elsewhere classified	622		0.63 (0.48-0.83)	Yes		0.72 (0.56-0.92)	
**XI Digestive**							
K11 Diseases of salivary glands	208		0.50 (0.33-0.75)	Yes		0.72 (0.50-1.03)	
K29 Gastritis and duodenitis	17,376		0.90 (0.86-0.94)	Yes		1.00 (0.97-1.04)	
K30 Dyspepsia	2,881		0.74 (0.67-0.82)	Yes		0.97 (0.90-1.06)	
K40 Inguinal hernia	4,189		1.20 (1.12-1.29)	Yes		1.11 (1.01-1.21)	
K59 Other functional intestinal disorders	682		0.60 (0.46-0.76)	Yes		0.89 (0.74-1.06)	
K74 Fibrosis and cirrhosis of liver	1,898		0.77 (0.68-0.87)	Yes		0.83 (0.74-0.93)	
K76 Other diseases of liver	1,978		0.73 (0.64-0.82)	Yes		0.96 (0.87-1.07)	
K80 Cholelithiasis	11,545		0.76 (0.72-0.80)	Yes		0.88 (0.85-0.92)	
K81 Cholecystitis	6,585		0.86 (0.81-0.92)	Yes		0.89 (0.85-0.94)	
K85 Acute pancreatitis	2,120		0.86 (0.77-0.96)			0.74 (0.67-0.82)	Yes
K86 Other diseases of pancreas	221		0.53 (0.37-0.76)	Yes		0.74 (0.54-1.01)	
K92 Other diseases of digestive system	4,464		0.83 (0.76-0.90)	Yes		0.90 (0.83-0.97)	
**XIII Musculoskeletal**							
M10 Gout	1,315		0.70 (0.60-0.82)	Yes		0.70 (0.58-0.83)	Yes
M32 Systemic lupus erythematosus	199		0.48 (0.30-0.76)	Yes		0.67 (0.49-0.91)	
M47 Spondylosis	17,121		0.83 (0.80-0.86)	Yes		0.97 (0.94-1.00)	
M50 Cervical disc disorders	3,062		0.80 (0.73-0.89)	Yes		0.92 (0.85-0.99)	
M75 Shoulder lesions	1,009		0.73 (0.62-0.85)	Yes		0.89 (0.77-1.02)	
M81 Osteoporosis without pathological fracture	1,832		0.85 (0.70-1.02)			0.65 (0.58-0.74)	Yes
**XIV Genitourinary**							
N10 Acute tubulo-interstitial nephritis	899		0.98 (0.80-1.21)			0.64 (0.54-0.76)	Yes
N11 Chronic tubulo-interstitial nephritis	129		0.34 (0.17-0.69)	Yes		0.77 (0.52-1.14)	
N18 Chronic kidney disease	1,867		0.66 (0.57-0.76)	Yes		0.58 (0.51-0.67)	Yes
N19 Unspecified kidney failure	1,769		0.60 (0.52-0.70)	Yes		0.68 (0.59-0.77)	Yes
N39 Other disorders of urinary system	4,183		0.86 (0.78-0.94)			0.80 (0.74-0.86)	Yes
N40 Hyperplasia of prostate	3,790		0.76 (0.69-0.84)	Yes		1.03 (0.93-1.14)	
N41 Inflammatory diseases of prostate	781		0.68 (0.57-0.80)	Yes		1.00 (0.82-1.22)	
N73 Other female pelvic inflammatory diseases	2,524		0.82 (0.75-0.91)	Yes		0.92 (0.86-0.99)	
**XVIII Other symptoms, signs and abnormal findings**							
R06 Abnormalities of breathing	223		0.48 (0.30-0.78)	Yes		0.61 (0.41-0.91)	
R10 Abdominal and pelvic pain	3,821		0.85 (0.78-0.93)	Yes		0.91 (0.84-0.98)	
R51 Headache	2,851		0.84 (0.76-0.92)	Yes		0.95 (0.88-1.04)	
R69 Unknown and unspecified causes of morbidity	21,300		0.83 (0.80-0.86)	Yes		0.89 (0.86-0.92)	Yes
R73 Elevated blood glucose level	147		0.23 (0.12-0.45)	Yes		0.94 (0.65-1.36)	
R96 Other sudden death, cause unknown	221		0.41 (0.23-0.72)	Yes		0.36 (0.21-0.64)	Yes
R99 Other ill-defined and unspecified causes of mortality	700		0.64 (0.47-0.86)	Yes		0.58 (0.45-0.75)	Yes
**XIX Injury, poisoning and other external causes**							
S22 Fracture of rib(s), sternum and thoracic spine	2,477		1.23 (1.11-1.35)	Yes		1.06 (0.96-1.16)	
S72 Fracture of femur	3,561		0.86 (0.77-0.96)			0.76 (0.70-0.83)	Yes
T14 Injury of unspecified body region	5,419		1.24 (1.16-1.32)	Yes		0.97 (0.91-1.04)	
**XX External causes**							
W01 Fall on same level from slipping, tripping and stumbling	245		0.46 (0.27-0.76)	Yes		1.35 (0.99-1.84)	
		0.2 0.5 1.0 2.0			0.2 0.5 1.0 2.0		

HR: hazard ratio.

The x-axis is on a log scale. HRs were stratified by age at risk (5-year groups), sex and ten study areas, and were adjusted for education, drinking status, smoking status and LIPA or MVPA.

Figure S25: Adjusted HRs for specific diseases showing significant associations with physical activity after FDR adjustment by ICD-10 chapters in men and women

Disease	No.event_women	Q5 v.s. Q1 in Women	HR (95%CI)	Sig.FDR P women	No.event_men	Q5 v.s. Q1 in Men	HR (95%CI)	Sig.FDR P men	P for interaction
**I Infectious and parasitic**									
B18 Chronic viral hepatitis	404		0.79 (0.60-1.05)		484		0.57 (0.46-0.71)	Yes	0.622
**II Neoplasms**									
C22 Malignant neoplasm of liver and intrahepatic bile ducts	1,171		0.67 (0.55-0.83)	Yes	2,193		0.77 (0.69-0.86)	Yes	0.188
C23-C24 Biliary cancer other than the liver	437		0.90 (0.68-1.20)		344		0.46 (0.33-0.65)	Yes	<0.001
C34 Malignant neoplasm of bronchus and lung	2,850		0.86 (0.76-0.98)		4,191		0.84 (0.77-0.91)	Yes	0.010
D44 Neoplasm of uncertain or unknown behaviour of endocrine glands	743		1.01 (0.83-1.23)		164		0.43 (0.27-0.69)	Yes	0.002
**III Blood and immune-related**									
D64 Other anaemias	1,196		0.71 (0.61-0.84)	Yes	582		0.72 (0.57-0.91)		0.238
D69 Purpura and other haemorrhagic conditions	523		0.61 (0.47-0.78)	Yes	292		0.58 (0.43-0.79)		0.284
**IV Endocrine, nutritional and metabolic**									
E10-E14 Diabetes mellitus	11,337		0.81 (0.77-0.86)	Yes	7,111		0.72 (0.68-0.76)	Yes	0.167
E78 Disorders of lipoprotein metabolism and other lipidaemias	2,002		0.87 (0.76-1.00)		837		0.66 (0.55-0.80)	Yes	0.033
E79 Disorders of purine and pyrimidine metabolism	198		0.84 (0.55-1.30)		129		0.37 (0.23-0.61)	Yes	0.05
E88 Other metabolic disorders	246		0.44 (0.28-0.70)	Yes	215		0.50 (0.33-0.77)		0.984
**V Mental and behavioural**									
F20 Schizophrenia	381		0.33 (0.24-0.46)	Yes	233		0.54 (0.40-0.72)	Yes	0.430
**VI Nerve-related**									
G31 Other degenerative diseases of nervous system, not elsewhere classified	397		0.49 (0.33-0.75)	Yes	431		0.64 (0.47-0.88)		0.061
G40 Epilepsy	351		0.42 (0.29-0.61)	Yes	467		0.57 (0.45-0.73)	Yes	0.542
**VII Eye and adnexa**									
H25, H26.9 Cataract	11,307		0.82 (0.76-0.88)	Yes	6,241		0.96 (0.89-1.04)		0.308
H43 Disorders of vitreous body	299		0.47 (0.31-0.70)	Yes	193		0.77 (0.51-1.15)		0.158
**IX Circulatory**									
I05-I09 Chronic rheumatic heart disease	444		0.67 (0.50-0.90)		225		0.51 (0.36-0.74)	Yes	0.238
I10 Essential (primary) hypertension	7,116		0.83 (0.76-0.89)	Yes	3,714		0.84 (0.77-0.92)	Yes	0.796
I20 Angina pectoris	1,460		0.62 (0.51-0.76)	Yes	978		0.81 (0.67-0.98)		0.025
I21 Acute myocardial infarction	3,075		0.62 (0.54-0.72)	Yes	3,859		0.72 (0.65-0.79)	Yes	<0.001
I25 Chronic ischaemic heart disease	23,177		0.73 (0.70-0.77)	Yes	14,794		0.73 (0.70-0.77)	Yes	0.112
I27 Other pulmonary heart diseases	1,157		0.61 (0.51-0.73)	Yes	1,158		0.52 (0.43-0.62)	Yes	<0.001
I46 Cardiac arrest	269		0.37 (0.22-0.62)	Yes	302		0.77 (0.54-1.08)		0.078
I47-I49 Cardiac arrhythmias	3,138		0.80 (0.71-0.89)	Yes	1,971		0.88 (0.78-0.98)		0.422
I50 Heart failure	1,971		0.85 (0.74-0.98)		1,547		0.68 (0.58-0.78)	Yes	0.299
I51 Complications and ill-defined descriptions of heart disease	1,252		0.51 (0.41-0.64)	Yes	898		0.65 (0.55-0.77)	Yes	0.156
I61 Intracerebral haemorrhage	4,914		0.73 (0.67-0.80)	Yes	5,487		0.80 (0.74-0.86)	Yes	0.006
I63 Cerebral infarction	25,990		0.76 (0.72-0.79)	Yes	20,563		0.79 (0.76-0.82)	Yes	0.236
I65-I66 Occlusion and stenosis of precerebral or cerebral arteries, not resulting in cerebral infarction	1,571		0.77 (0.63-0.94)		1,344		0.69 (0.58-0.82)	Yes	0.638
I69 Sequelae of cerebrovascular disease	2,651		0.63 (0.54-0.74)	Yes	2,981		0.65 (0.58-0.72)	Yes	0.469
I70 Atherosclerosis	647		0.69 (0.52-0.92)		560		0.64 (0.49-0.82)	Yes	0.004
I83, I85, I86 Varicose veins	1,562		1.35 (1.20-1.51)	Yes	1,824		1.39 (1.28-1.51)	Yes	0.050
**X Respiratory**									
J12-J18 Pneumonia	16,593		0.89 (0.85-0.94)	Yes	11,677		0.84 (0.80-0.89)	Yes	<0.001
J44 Other chronic obstructive pulmonary disease	4,332		0.82 (0.74-0.90)	Yes	4,663		0.79 (0.73-0.86)	Yes	<0.001
J96 Respiratory failure, not elsewhere classified	270		0.67 (0.40-1.11)		352		0.49 (0.33-0.73)	Yes	0.540
**XI Digestive**									
K29 Gastritis and duodenitis	11,796		0.93 (0.88-0.98)		5,580		0.88 (0.82-0.94)	Yes	0.178
K30 Dyspepsia	1,864		0.78 (0.69-0.88)		1,017		0.68 (0.57-0.81)	Yes	0.245
K40 Inguinal hernia	392		0.92 (0.69-1.22)		3,797		1.36 (1.27-1.47)	Yes	0.761
K76 Other diseases of liver	1,265		0.61 (0.52-0.73)	Yes	713		0.75 (0.62-0.90)		0.654
K80 Cholelithiasis	8,503		0.78 (0.74-0.82)	Yes	3,042		0.69 (0.63-0.75)	Yes	<0.001
K92 Other diseases of digestive system	1,954		0.74 (0.64-0.85)	Yes	2,510		0.83 (0.75-0.91)	Yes	0.647
**XIII Musculoskeletal**									
M35 Other systemic involvement of connective tissue	383		0.54 (0.39-0.73)	Yes					
M47 Spondylosis	12,045		0.93 (0.89-0.97)		5,076		0.86 (0.81-0.92)	Yes	<0.001
M81 Osteoporosis without pathological fracture	1,566		0.69 (0.56-0.84)	Yes	266		0.92 (0.59-1.44)		0.540
**XIV Genitourinary**									
N03 Chronic nephritic syndrome	529		0.61 (0.48-0.79)	Yes	392		0.80 (0.61-1.05)		0.344
N18 Chronic kidney disease	899		0.53 (0.42-0.68)	Yes	968		0.58 (0.47-0.71)	Yes	0.178
N19 Unspecified kidney failure	884		0.57 (0.44-0.73)	Yes	885		0.61 (0.50-0.74)	Yes	0.337
N40 Hyperplasia of prostate					3,767		0.75 (0.68-0.83)	Yes	
**XVIII Other symptoms, signs and abnormal findings**									
R10 Abdominal and pelvic pain	2,384		0.74 (0.65-0.83)	Yes	1,437		0.94 (0.83-1.07)		0.08
R53 Malaise and fatigue	306		0.49 (0.36-0.68)	Yes	207		1.00 (0.75-1.32)		0.435
R69 Unknown and unspecified causes of morbidity	13,191		0.76 (0.73-0.80)	Yes	8,109		0.81 (0.77-0.85)	Yes	<0.001
R99 Other ill-defined and unspecified causes of mortality	320		0.81 (0.50-1.31)		380		0.54 (0.36-0.80)	Yes	0.728
		0.2 0.5 1.0 2.0				0.2 0.5 1.0 2.0			

HR: hazard ratio.

The x-axis is on a log scale. HRs were stratified by age at risk (5-year groups), sex and ten study areas, and were adjusted for education, drinking status and smoking status.

Figure S26: Adjusted HRs for specific diseases showing significant associations with physical activity after FDR adjustment in age group

Disease	No.event_<65 years	<65 years	HR (95%CI)	Sig.FDR P	WHO (18-64years)	No.event_≥65 years	≥65 years	HR (95%CI)	Sig.FDR P	WHO (≥65years)	P for interaction
**I Infectious and parasitic**											
B18 Chronic viral hepatitis	783		0.69 (0.58-0.82)	Yes		105		0.49 (0.18-1.36)			0.728
B99 Other and unspecified infectious diseases	763		0.70 (0.61-0.81)	Yes		184		0.74 (0.44-1.27)			0.621
**II Neoplasms**											
C22 Malignant neoplasm of liver and intrahepatic bile ducts	2,426		0.76 (0.69-0.84)	Yes		938		0.67 (0.48-0.93)			0.672
C34 Malignant neoplasm of bronchus and lung	4,591		0.87 (0.81-0.93)	Yes	Critical	2,450		0.80 (0.65-0.99)		Critical	0.444
C50 Malignant neoplasm of breast	2,542		0.81 (0.72-0.91)	Yes	Critical	296		0.33 (0.08-1.38)		Critical	0.085
**III Blood and immune-related**											
D64 Other anaemias	1,222		0.65 (0.56-0.74)	Yes		556		0.93 (0.63-1.36)			0.233
D69 Purpura and other haemorrhagic conditions	664		0.58 (0.48-0.71)	Yes		151		0.61 (0.25-1.50)			0.555
**IV Endocrine, nutritional and metabolic**											
E10-E14 Diabetes mellitus	15,500		0.77 (0.74-0.80)	Yes	Critical	2,948		0.79 (0.67-0.94)		Critical	0.719
E78 Disorders of lipoprotein metabolism and other lipidaemias	2,414		0.77 (0.69-0.86)	Yes		425		0.91 (0.52-1.58)			0.905
E88 Other metabolic disorders	318		0.37 (0.26-0.52)	Yes		143		0.99 (0.49-2.01)			0.325
**V Mental and behavioural**											
F01-F03 Dementia	123		0.42 (0.25-0.71)	Yes	Important	186		0.51 (0.26-1.00)		Important	0.543
**VI Nerve-related**											
G31 Other degenerative diseases of nervous system, not elsewhere classified	393		0.51 (0.39-0.67)	Yes		435		0.74 (0.45-1.20)			0.566
G40 Epilepsy	637		0.52 (0.43-0.64)	Yes		181		0.37 (0.13-1.01)			0.836
**VII Eye and adnexa**											
H25, H26.9 Cataract	10,382		0.85 (0.80-0.90)	Yes		7,166		0.94 (0.82-1.07)			0.194
**IX Circulatory**											
I05-I09 Chronic rheumatic heart disease	504		0.60 (0.48-0.75)	Yes		165		0.42 (0.15-1.14)			0.431
I10 Essential (primary) hypertension	8,887		0.82 (0.77-0.87)	Yes	Important	1,943		0.91 (0.70-1.19)		Important	0.665
I20 Angina pectoris	1,763		0.74 (0.64-0.85)	Yes		675		0.55 (0.31-0.99)			0.313
I21 Acute myocardial infarction	3,922		0.70 (0.65-0.77)	Yes		3,012		0.61 (0.49-0.76)	Yes		0.734
I25 Chronic ischaemic heart disease	26,938		0.75 (0.72-0.78)	Yes	Critical	11,033		0.64 (0.56-0.73)	Yes	Critical	0.031
I27 Other pulmonary heart diseases	1,348		0.59 (0.51-0.68)	Yes		967		0.44 (0.31-0.61)	Yes		0.075
I47-I49 Cardiac arrhythmias	3,761		0.83 (0.77-0.91)	Yes		1,348		1.03 (0.82-1.31)			0.064
I50 Heart failure	1,932		0.68 (0.61-0.76)	Yes		1,586		0.87 (0.70-1.09)			0.301
I51 Complications and ill-defined descriptions of heart disease	1,564		0.61 (0.53-0.70)	Yes		586		0.51 (0.32-0.82)			0.697
I61 Intracerebral haemorrhage	6,614		0.76 (0.72-0.81)	Yes	Critical	3,787		0.82 (0.71-0.96)		Critical	0.588
I63 Cerebral infarction	32,709		0.76 (0.74-0.79)	Yes	Critical	13,844		0.85 (0.77-0.93)	Yes	Critical	0.457
I65-I66 Occlusion and stenosis of precerebral or cerebral arteries, not resulting in cerebral infarction	1,901		0.68 (0.59-0.78)	Yes		1,014		1.00 (0.68-1.47)			0.214
I69 Sequelae of cerebrovascular disease	3,602		0.66 (0.60-0.72)	Yes		2,030		0.60 (0.47-0.78)	Yes		0.368
I70 Atherosclerosis	842		0.67 (0.56-0.82)	Yes		365		0.50 (0.25-1.01)			0.729
I83, I85, I86 Varicose veins	3,067		1.37 (1.28-1.46)	Yes		319		1.07 (0.71-1.61)			0.164
**X Respiratory**											
J12-J18 Pneumonia	20,637		0.88 (0.85-0.91)	Yes		7,633		0.81 (0.71-0.92)	Yes		0.864
J44 Other chronic obstructive pulmonary disease	5,372		0.82 (0.76-0.88)	Yes		3,623		0.79 (0.67-0.92)			0.906
J96 Respiratory failure, not elsewhere classified	356		0.55 (0.40-0.76)	Yes		266		0.42 (0.15-1.17)			0.875
**XI Digestive**											
K29 Gastritis and duodenitis	14,122		0.91 (0.87-0.95)	Yes		3,254		0.78 (0.63-0.96)			0.309
K30 Dyspepsia	2,357		0.72 (0.65-0.80)	Yes		524		0.62 (0.40-0.97)			0.236
K40 Inguinal hernia	3,094		1.19 (1.11-1.28)	Yes		1,095		1.75 (1.40-2.19)	Yes		0.008
K74 Fibrosis and cirrhosis of liver	1,521		0.73 (0.65-0.83)	Yes		377		1.01 (0.65-1.59)			0.179
K76 Other diseases of liver	1,672		0.66 (0.58-0.74)	Yes		306		0.75 (0.42-1.33)			0.864
K80 Cholelithiasis	9,807		0.75 (0.71-0.78)	Yes		1,738		0.67 (0.52-0.87)			0.003
K81 Cholecystitis	5,330		0.86 (0.80-0.91)	Yes		1,255		0.78 (0.58-1.06)			0.491
K92 Other diseases of digestive system	3,100		0.81 (0.74-0.88)	Yes		1,364		0.85 (0.67-1.09)			0.423
**XIII Musculoskeletal**											
M47 Spondylosis	15,185		0.90 (0.86-0.93)	Yes		1,936		1.13 (0.91-1.40)			0.193
M81 Osteoporosis without pathological fracture	1,090		0.70 (0.58-0.85)	Yes		742		0.72 (0.42-1.24)		Critical	0.597
**XIV Genitourinary**											
N18 Chronic kidney disease	1,319		0.53 (0.45-0.62)	Yes		548		0.62 (0.34-1.12)			0.673
N19 Unspecified kidney failure	1,184		0.64 (0.55-0.75)	Yes		585		0.46 (0.27-0.78)			0.487
N40 Hyperplasia of prostate	2,080		0.82 (0.73-0.91)			1,710		0.65 (0.51-0.84)	Yes		0.104
N81 Female genital prolapse	762		1.47 (1.23-1.76)	Yes		185		0.54 (0.17-1.76)			0.179
**XVIII Other symptoms, signs and abnormal findings**											
R10 Abdominal and pelvic pain	2,949		0.79 (0.72-0.87)	Yes		872		0.92 (0.66-1.28)			0.335
R69 Unknown and unspecified causes of morbidity	16,450		0.80 (0.77-0.83)	Yes		4,850		0.74 (0.65-0.85)	Yes		0.717
**XIX Injury, poisoning and other external causes**											
T14 Injury of unspecified body region	4,636		1.19 (1.12-1.27)	Yes		783		1.10 (0.78-1.56)			0.359
		0.2 0.5 1.0 2.0					0.2 0.5 1.0 2.0				

HR: hazard ratio.

The x-axis is on a log scale. HRs were stratified by age at risk (5-year groups), sex and ten study areas, and were adjusted for education, drinking status and smoking status.

Figure S27: Wide landscapes of diseases associated with the highest quintile group of physical activity after FDR adjustment by region

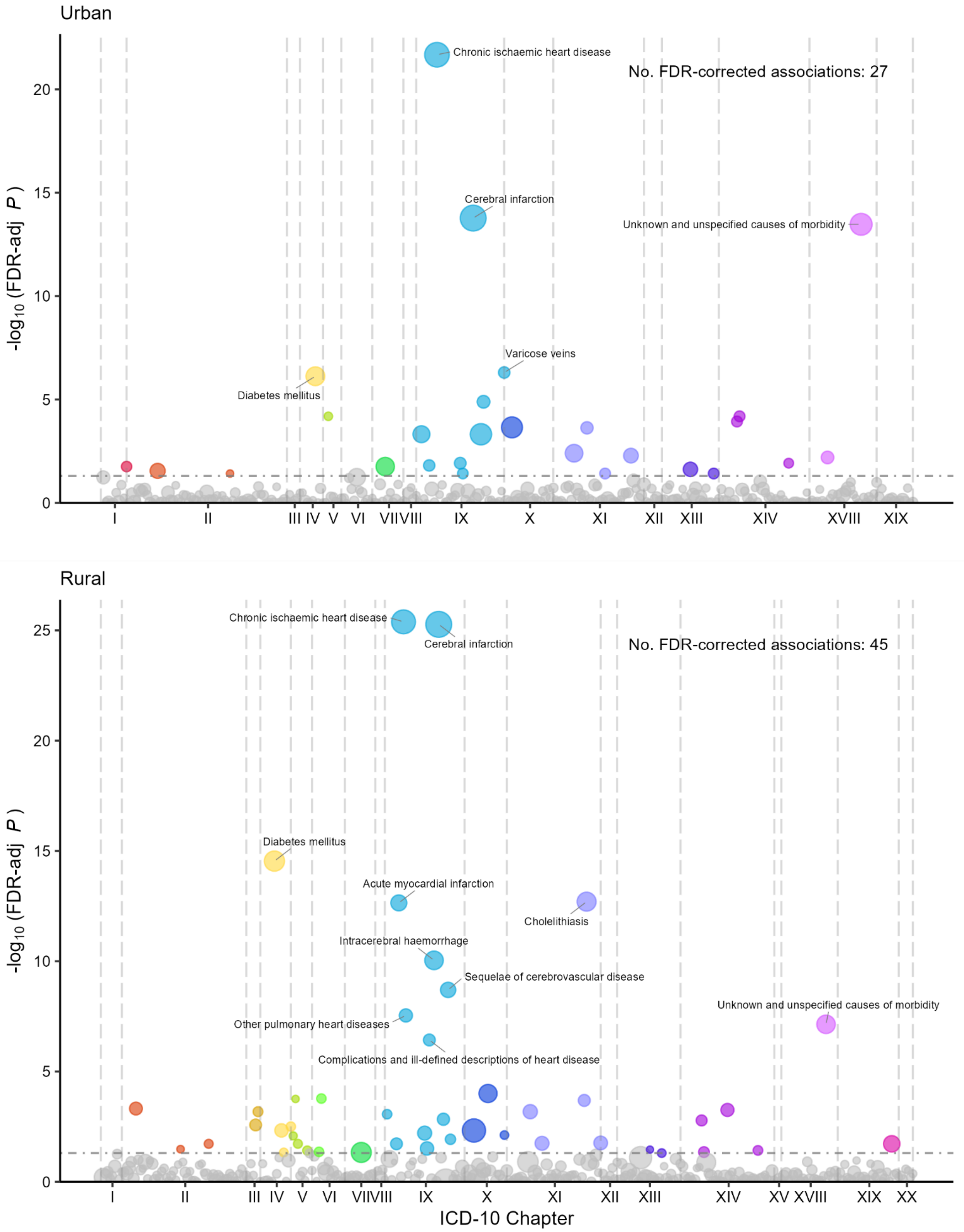

The y-axis represents the negative log (10) of the phenome-wide *P* value after FDR adjustment. Horizontal gray dashed line indicates the cut-off for 0.05. The size of the point is proportional to the number of cases. The models were stratified by age at risk (5-year groups), sex and ten study areas, and were adjusted for education, drinking status and smoking status.

Figure S28: Adjusted HRs for specific diseases showing significant associations with physical activity after FDR adjustment by ICD-10 chapters in rural and urban regions

Disease	No.event_urban	Q5 v.s. Q1 in urban	HR (95%CI)	Sig.FDR P urban	No.event_rural	Q5 v.s. Q1 in rural	HR (95%CI)	Sig.FDR P rural	P for interaction
**I Infectious and parasitic**									
B99 Other and unspecified infectious diseases	767		0.69 (0.59-0.80)	Yes	180		0.66 (0.49-0.89)		0.675
**II Neoplasms**									
C22 Malignant neoplasm of liver and intrahepatic bile ducts	1,487		0.81 (0.68-0.97)		1,877		0.71 (0.64-0.80)	Yes	0.245
C34 Malignant neoplasm of bronchus and lung	3,636		0.80 (0.71-0.91)	Yes	3,405		0.87 (0.80-0.95)		0.011
C78 Secondary malignant neoplasm of respiratory and digestive organs	217		0.89 (0.57-1.38)		141		0.44 (0.30-0.65)	Yes	0.901
D14 Benign neoplasm of middle ear and respiratory system	124		0.35 (0.19-0.63)	Yes					
D18 Haemangioma and lymphangioma, any site	263		0.94 (0.65-1.35)		348		0.56 (0.44-0.71)	Yes	0.162
**III Blood and immune-related**									
D64 Other anaemias	497		0.89 (0.67-1.20)		1,281		0.67 (0.58-0.78)	Yes	0.001
D69 Purpura and other haemorrhagic conditions	254		0.74 (0.46-1.19)		561		0.52 (0.43-0.65)	Yes	0.533
**IV Endocrine, nutritional and metabolic**									
E10-E14 Diabetes mellitus	8,267		0.79 (0.74-0.84)	Yes	10,181		0.74 (0.71-0.77)	Yes	0.013
E78 Disorders of lipoprotein metabolism and other lipidaemias	650		0.64 (0.44-0.93)		2,189		0.74 (0.66-0.83)	Yes	<0.001
E79 Disorders of purine and pyrimidine metabolism					297		0.55 (0.40-0.76)	Yes	
E88 Other metabolic disorders					431		0.47 (0.34-0.65)	Yes	
**V Mental and behavioural**									
F01-F03 Dementia	108		0.87 (0.40-1.90)		201		0.38 (0.23-0.62)	Yes	0.408
F06 Other mental disorders due to brain damage and dysfunction and to physical disease					120		0.21 (0.12-0.37)	Yes	
F20 Schizophrenia	274		0.33 (0.23-0.49)	Yes	340		0.55 (0.42-0.71)	Yes	0.293
F45 Somatoform disorders	323		0.92 (0.67-1.26)		388		0.58 (0.45-0.76)	Yes	0.008
**VI Nerve-related**									
G31 Other degenerative diseases of nervous system, not elsewhere classified	366		0.43 (0.24-0.77)		462		0.61 (0.47-0.81)	Yes	0.233
G40 Epilepsy	359		0.66 (0.47-0.93)		459		0.47 (0.37-0.60)	Yes	0.551
**VII Eye and adnexa**									
H25, H26.9 Cataract	7,720		0.80 (0.70-0.91)	Yes	9,828		0.90 (0.85-0.95)	Yes	<0.001
**IX Circulatory**									
I05-I09 Chronic rheumatic heart disease	201		1.05 (0.61-1.82)		468		0.51 (0.40-0.65)	Yes	0.644
I10 Essential (primary) hypertension	5,689		0.77 (0.70-0.86)	Yes	5,141		0.88 (0.82-0.94)		<0.001
I20 Angina pectoris	1,153		0.60 (0.45-0.79)	Yes	1,285		0.73 (0.62-0.85)	Yes	0.017
I21 Acute myocardial infarction	2,530		0.82 (0.69-0.98)		4,404		0.64 (0.58-0.70)	Yes	0.316
I25 Chronic ischaemic heart disease	20,337		0.71 (0.66-0.75)	Yes	17,634		0.73 (0.70-0.76)	Yes	<0.001
I27 Other pulmonary heart diseases	226		0.38 (0.18-0.82)		2,089		0.59 (0.52-0.68)	Yes	<0.001
I47-I49 Cardiac arrhythmias	2,445		0.85 (0.73-0.98)		2,664		0.78 (0.72-0.86)	Yes	<0.001
I50 Heart failure	1,294		0.62 (0.48-0.80)	Yes	2,224		0.80 (0.72-0.89)	Yes	<0.001
I51 Complications and ill-defined descriptions of heart disease	823		0.66 (0.51-0.84)	Yes	1,327		0.56 (0.48-0.66)	Yes	0.007
I61 Intracerebral haemorrhage	2,794		0.87 (0.76-0.99)		7,607		0.75 (0.70-0.80)	Yes	<0.001
I63 Cerebral infarction	23,837		0.78 (0.74-0.82)	Yes	22,716		0.77 (0.74-0.80)	Yes	<0.001
I65-I66 Occlusion and stenosis of precerebral or cerebral arteries, not resulting in cerebral infarction	1,420		0.81 (0.63-1.05)		1,495		0.68 (0.58-0.79)	Yes	0.006
I67 Other cerebrovascular diseases	12,843		0.85 (0.79-0.90)	Yes	19,131		1.00 (0.96-1.04)		<0.001
I69 Sequelae of cerebrovascular disease	1,893		0.58 (0.48-0.70)	Yes	3,739		0.67 (0.60-0.74)	Yes	<0.001
I70 Atherosclerosis	493		0.66 (0.44-0.99)		714		0.63 (0.51-0.77)	Yes	<0.001
I83, I85, I86 Varicose veins	1,200		1.82 (1.59-2.08)	Yes	2,186		1.16 (1.08-1.25)		0.068
**X Respiratory**									
J12-J18 Pneumonia	12,130		0.84 (0.79-0.90)	Yes	16,140		0.90 (0.86-0.94)	Yes	<0.001
J44 Other chronic obstructive pulmonary disease	2,027		0.78 (0.63-0.97)		6,968		0.81 (0.76-0.87)	Yes	<0.001
J96 Respiratory failure, not elsewhere classified	346		0.70 (0.40-1.23)		276		0.45 (0.31-0.65)	Yes	0.302
**XI Digestive**									
K29 Gastritis and duodenitis	6,586		0.83 (0.76-0.90)	Yes	10,790		0.92 (0.88-0.97)		<0.001
K30 Dyspepsia					2,802		0.73 (0.66-0.81)	Yes	
K40 Inguinal hernia	1,621		1.51 (1.31-1.74)	Yes	2,568		1.24 (1.15-1.34)	Yes	<0.001
K62 Other diseases of anus and rectum	887		0.66 (0.53-0.83)	Yes	807		1.04 (0.89-1.22)		0.071
K76 Other diseases of liver	611		0.75 (0.57-0.99)		1,367		0.63 (0.55-0.72)	Yes	<0.001
K80 Cholelithiasis	3,335		0.79 (0.71-0.87)	Yes	8,210		0.72 (0.69-0.76)	Yes	<0.001
K92 Other diseases of digestive system	2,074		0.81 (0.70-0.93)		2,390		0.79 (0.72-0.88)	Yes	0.571
**XIII Musculoskeletal**									
M47 Spondylosis	3,085		0.79 (0.70-0.89)	Yes	14,036		0.92 (0.89-0.96)		<0.001
M53 Other dorsopathies, not elsewhere classified					107		0.34 (0.19-0.60)	Yes	
M71 Other bursopathies					292		2.01 (1.64-2.47)	Yes	
M81 Osteoporosis without pathological fracture	870		0.48 (0.30-0.77)	Yes	962		0.74 (0.61-0.90)		<0.001
**XIV Genitourinary**									
N18 Chronic kidney disease	927		0.48 (0.36-0.64)	Yes	940		0.63 (0.52-0.75)	Yes	0.012
N19 Unspecified kidney failure	910		0.48 (0.36-0.63)	Yes	859		0.71 (0.59-0.85)	Yes	0.006
N40 Hyperplasia of prostate	1,791		0.80 (0.66-0.97)		1,999		0.72 (0.64-0.81)	Yes	0.003
N73 Other female pelvic inflammatory diseases	543		0.57 (0.45-0.73)	Yes	1,981		0.94 (0.85-1.03)		0.037
N81 Female genital prolapse	441		1.10 (0.76-1.58)		506		1.65 (1.35-2.02)	Yes	0.001
**XVIII Other symptoms, signs and abnormal findings**									
R10 Abdominal and pelvic pain	1,816		0.73 (0.63-0.84)	Yes	2,005		0.90 (0.81-1.01)		<0.001
R69 Unknown and unspecified causes of morbidity	13,828		0.79 (0.76-0.82)	Yes	7,472		0.76 (0.71-0.81)	Yes	<0.001
**XIX Injury, poisoning and other external causes**									
T14 Injury of unspecified body region	546		1.05 (0.81-1.35)		4,873		1.17 (1.09-1.25)	Yes	0.248

HR: hazard ratio.

The x-axis is on a log scale. HRs were stratified by age at risk (5-year groups), sex and ten study areas, and were adjusted for education, drinking status and smoking status.

Figure S29: Adjusted HRs for all-cause and cause-specific mortality associated with physical activity

Disease	No.event	HR (95%CI)	Sig.FDR P
**I Infectious and parasitic**			
B18 Chronic viral hepatitis	246	0.42 (0.29-0.60)	Yes
**II Neoplasms**			
C00-C14 Lip, oral cavity & pharynx cancer	256	1.16 (0.83-1.61)	
C15 Malignant neoplasm of oesophagus	1,594	0.84 (0.72-0.97)	
C16 Malignant neoplasm of stomach	2,165	0.87 (0.78-0.98)	
C18 Malignant neoplasm of colon	579	0.66 (0.49-0.88)	Yes
C20 Malignant neoplasm of rectum	654	0.94 (0.74-1.19)	
C22 Malignant neoplasm of liver and intrahepatic bile ducts	2,448	0.73 (0.65-0.82)	Yes
C23-C24 Biliary cancer other than the liver	331	0.68 (0.48-0.97)	
C25 Malignant neoplasm of pancreas	728	1.03 (0.84-1.26)	
C34 Malignant neoplasm of bronchus and lung	4,547	0.80 (0.73-0.88)	Yes
C40-C41 Malignant neoplasms of bone & articular cartilage	156	0.60 (0.36-1.01)	
C50 Malignant neoplasm of breast	411	0.67 (0.48-0.92)	
C53 Malignant neoplasm of cervix uteri	242	0.64 (0.44-0.95)	
C55 Malignant neoplasm of uterus, part unspecified	121	0.38 (0.17-0.83)	
C56 Malignant neoplasm of ovary	171	0.89 (0.55-1.42)	
C61 Malignant neoplasm of prostate	171	0.93 (0.54-1.60)	
C67 Malignant neoplasm of bladder	167	0.74 (0.43-1.28)	
C70-72 CNS cancer	363	0.66 (0.49-0.88)	
C82-C88 Non-Hodgkin lymphoma	150	0.99 (0.64-1.52)	
C90 Multiple myeloma and malignant plasma cell neoplasms	121	1.43 (0.90-2.26)	
C95 Leukaemia of unspecified cell type	235	0.76 (0.54-1.09)	
C96 Other and unspecified malignant neoplasms of lymphoid, haematopoietic and related tissue	110	0.66 (0.33-1.30)	
**IV Endocrine, nutritional and metabolic**			
E10-E14 Diabetes mellitus	373	0.48 (0.32-0.74)	Yes
**V Mental and behavioural**			
F01-F03 Dementia	113	0.59 (0.31-1.14)	
**VI Nerve-related**			
G31 Other degenerative diseases of nervous system, not elsewhere classified	172	0.60 (0.34-1.06)	
**IX Circulatory**			
I05-I09 Chronic rheumatic heart disease	171	0.26 (0.14-0.49)	Yes
I11 Hypertensive heart disease	117	0.77 (0.44-1.36)	
I21 Acute myocardial infarction	4,203	0.63 (0.56-0.70)	Yes
I25 Chronic ischaemic heart disease	2,510	0.58 (0.48-0.70)	Yes
I27 Other pulmonary heart diseases	162	0.31 (0.13-0.72)	Yes
I46 Cardiac arrest	191	1.03 (0.65-1.64)	
I60 Subarachnoid haemorrhage	129	0.62 (0.32-1.19)	
I61 Intracerebral haemorrhage	5,507	0.69 (0.63-0.75)	Yes
I63 Cerebral infarction	2,004	0.53 (0.44-0.63)	Yes
I64 Stroke, not specified as haemorrhage or infarction	343	0.71 (0.49-1.01)	
I67 Other cerebrovascular diseases	145	0.92 (0.47-1.80)	
I69 Sequelae of cerebrovascular disease	1,749	0.46 (0.38-0.56)	Yes
**X Respiratory**			
J12-J18 Pneumonia	654	0.43 (0.27-0.68)	Yes
J42 Unspecified chronic bronchitis	464	0.49 (0.34-0.70)	Yes
J44 Other chronic obstructive pulmonary disease	1,233	0.48 (0.39-0.60)	Yes
**XI Digestive**			
K25 Gastric ulcer	107	0.56 (0.27-1.18)	
K74 Fibrosis and cirrhosis of liver	320	0.50 (0.34-0.74)	Yes
**XIV Genitourinary**			
N03 Chronic nephritic syndrome	291	0.41 (0.26-0.65)	Yes
**XVIII Other symptoms, signs and abnormal findings**			
R96 Other sudden death, cause unknown	205	0.46 (0.26-0.81)	Yes
R99 Other ill-defined and unspecified causes of mortality	682	0.66 (0.49-0.90)	Yes
**XX External causes**			
V03 Pedestrian injured in collision with car, pick-up truck or van	351	1.05 (0.81-1.38)	
V13 Pedal cyclist injured in collision with car, pick-up truck or van	114	1.23 (0.88-1.73)	
V23 Motorcycle rider injured in collision with car, pick-up truck or van	117	1.27 (0.90-1.79)	
W01 Fall on same level from slipping, tripping and stumbling	234	0.58 (0.35-0.97)	
W13 Fall from, out of or through building or structure	136	1.32 (0.92-1.89)	
W70 Drowning and submersion following fall into natural water	103	0.79 (0.49-1.26)	
X68 Intentional self-poisoning by and exposure to pesticides	227	0.80 (0.58-1.10)	
X70 Intentional self-harm by hanging, strangulation and suffocation	227	0.79 (0.56-1.12)	
**Any event**	**55,052**	**0.60 (0.58-0.62)**	**Yes**

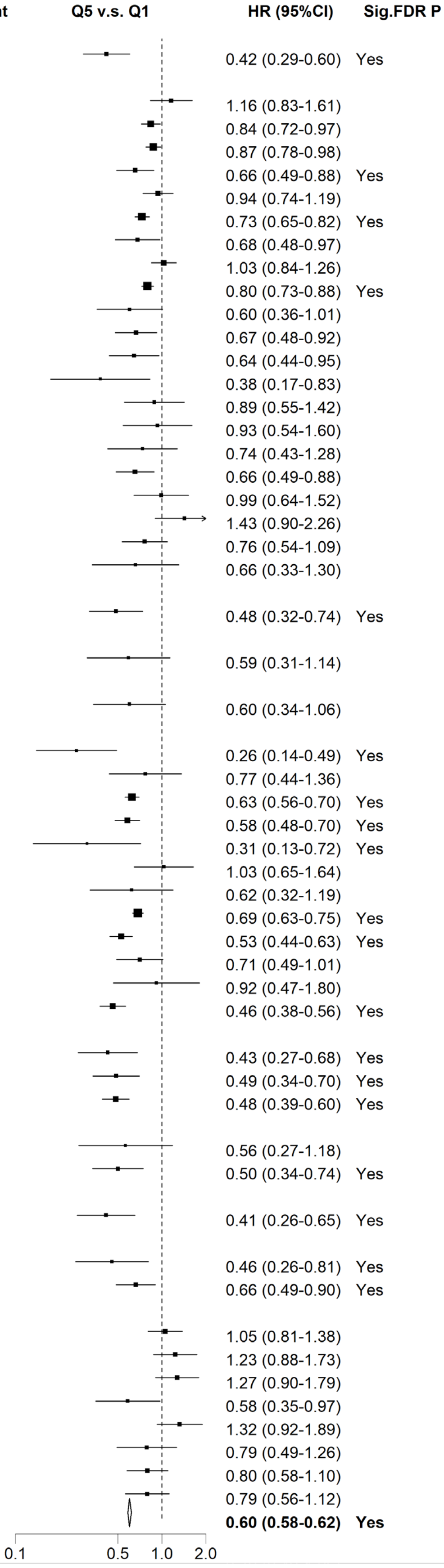

PA: physical activity. HR: hazard ratio.

The x-axis is on a log scale. HRs were stratified by age at risk (5-year groups), sex and ten study areas, and were adjusted for education, drinking status and smoking status.

Figure S30: Kaplan-Meier curves for overall survival

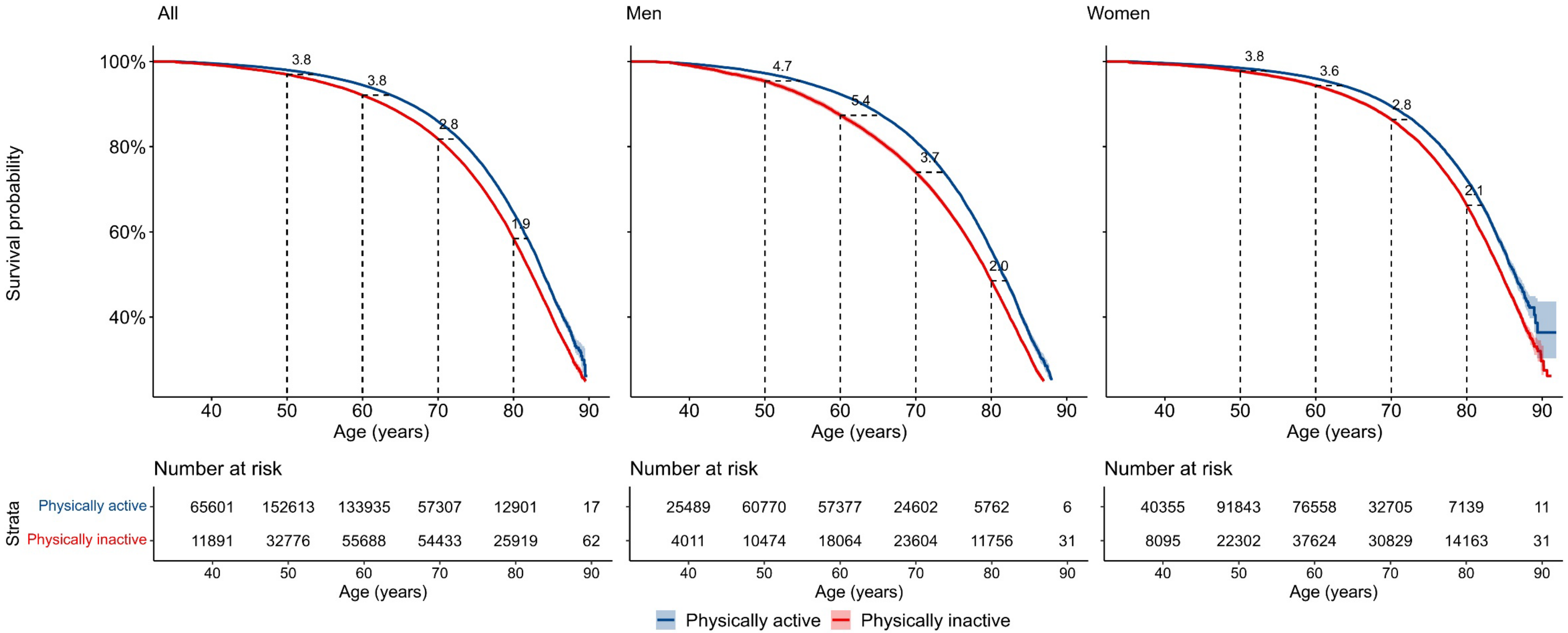

Figure S31: Total number of hospitalisations median days in hospital and from CKB PA-associated diseases

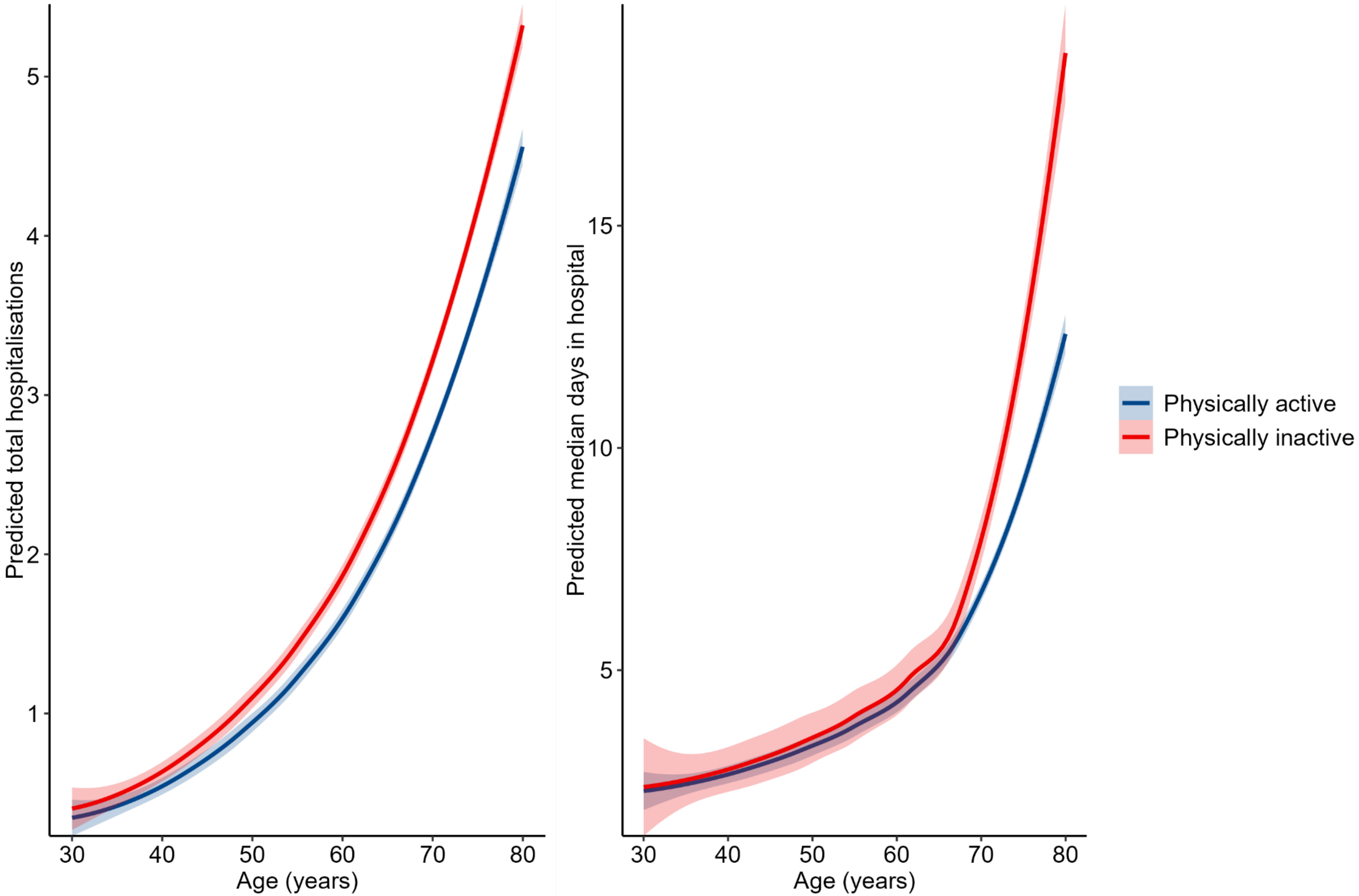

The total number of hospitalisations and median days spent in hospital for CKB PA-associated diseases were estimated using negative binomial regression and Gamma regression, respectively, adjusting for age, sex and 10 study areas.

Figure S32: Pearson correlation coefficients for accelerometer-estimated and self-reported PAs in the CKB third resurvey (n=20,190)

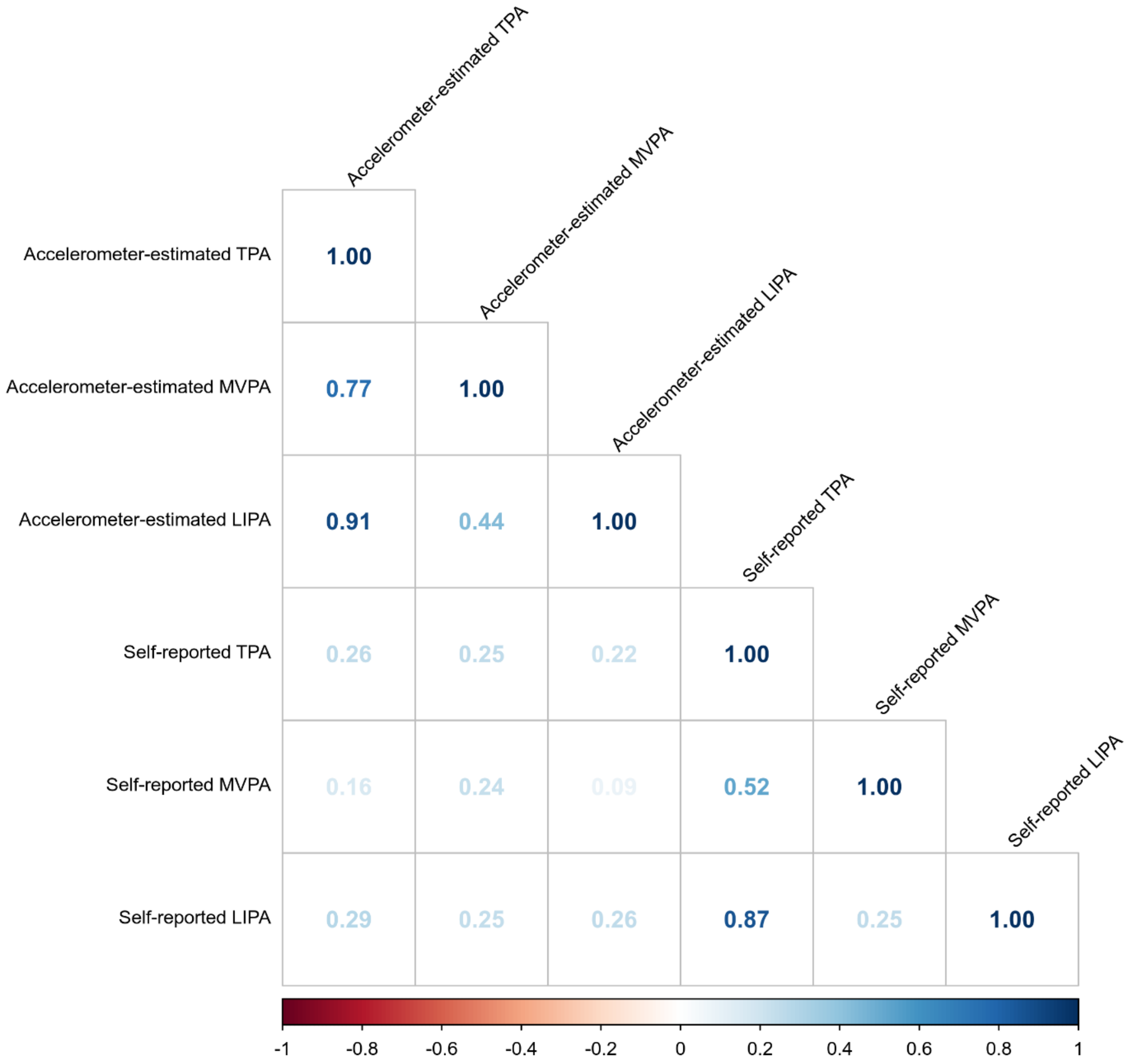

TPA: Total physical activity; MVPA: Moderate-to-vigorous intensity physical activity; LIPA: Low-intensity physical activity.
